# Supplementary material for: Underestimated Aspect of Mucopolysaccharidosis Pathogenesis: Global Changes in Cellular Processes Revealed by Transcriptomic Studies
Source: Int J Mol Sci. 2020 Feb 11;21(4):1204. doi: 10.3390/ijms21041204 (PMC7072725; doi:10.3390/ijms21041204)
Supplement: Supplementary file 1 [file ijms-21-01204-s001.pdf]

Table S1. Number of reads in RNA-seq analysis. MPS types are indicated by Arabic numbers.

Sample	f1*	f2*	Sample	f1*	f2*
**HDFa_I**	58718762	58718762	**MPS3D_I**	49250436	49250436
**HDFa_II**	47756883	47756883	**MPS3D_II**	54197694	54197694
**HDFa_III**	54168450	54168450	**MPS3D_III**	52067506	52067506
**HDFa_IV**	41614258	41614258	**MPS3D_IV**	56947314	56947314
**MPS1_I**	45793517	45793517	**MPS4A_I**	55486056	55486056
**MPS1_II**	52769993	52769993	**MPS4A_II**	53613161	53613161
**MPS1_III**	57129332	57129332	**MPS4A_III**	52384107	52384107
**MPS1_IV**	49847130	49847130	**MPS4A_IV**	51577667	51577667
**MPS2_I**	57537368	57537368	**MPS4B_I**	53972180	53972180
**MPS2_II**	40457828	40457828	**MPS4B_II**	45196042	45196042
**MPS2_III**	48051766	48051766	**MPS4B_III**	49530863	49530863
**MPS2_IV**	52908488	52908488	**MPS4B_IV**	50709120	50709120
**MPS3A_I**	52050375	52050375	**MPS6_I**	58345020	58345020
**MPS3A_II**	61670273	61670273	**MPS6_II**	60052676	60052676
**MPS3A_III**	43882338	43882338	**MPS6_III**	50965223	50965223
**MPS3A_IV**	50595389	50595389	**MPS6_IV**	44087469	44087469
**MPS3B_I**	54088730	54088730	**MPS7_I**	44555814	44555814
**MPS3B_II**	43987340	43987340	**MPS7_II**	47253718	47253718
**MPS3B_III**	60018865	60018865	**MPS7_III**	55918102	55918102
**MPS3B_IV**	44837985	44837985	**MPS7_IV**	46516804	46516804
**MPS3C_I**	59767727	59767727	**MPS9_I**	60501009	60501009
**MPS3C_II**	43732902	43732902	**MPS9_II**	43414644	43414644
**MPS3C_III**	53819135	53819135	**MPS9_III**	49440837	49440837
**MPS3C_IV**	47165191	47165191	**MPS9_IV**	45087234	45087234

*f1. f2 – number of reads from one and the other end

Figure S1. Number of up (red)- and down (blue)-regulated transcripts with division into various cellular processes (not included in Figure 2) in different MPS types relative to control cells (HDFa)

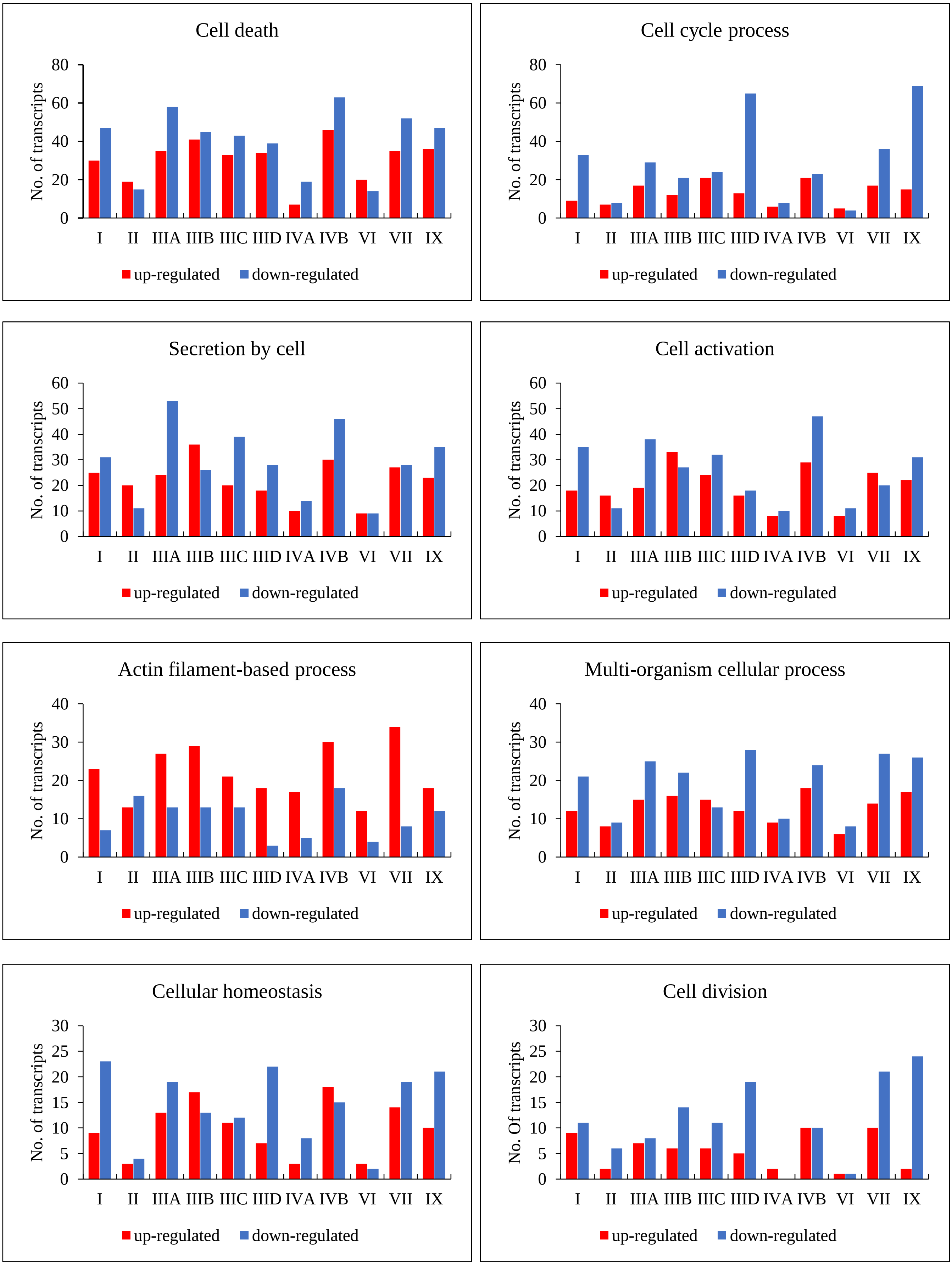

Figure S1 (continued).

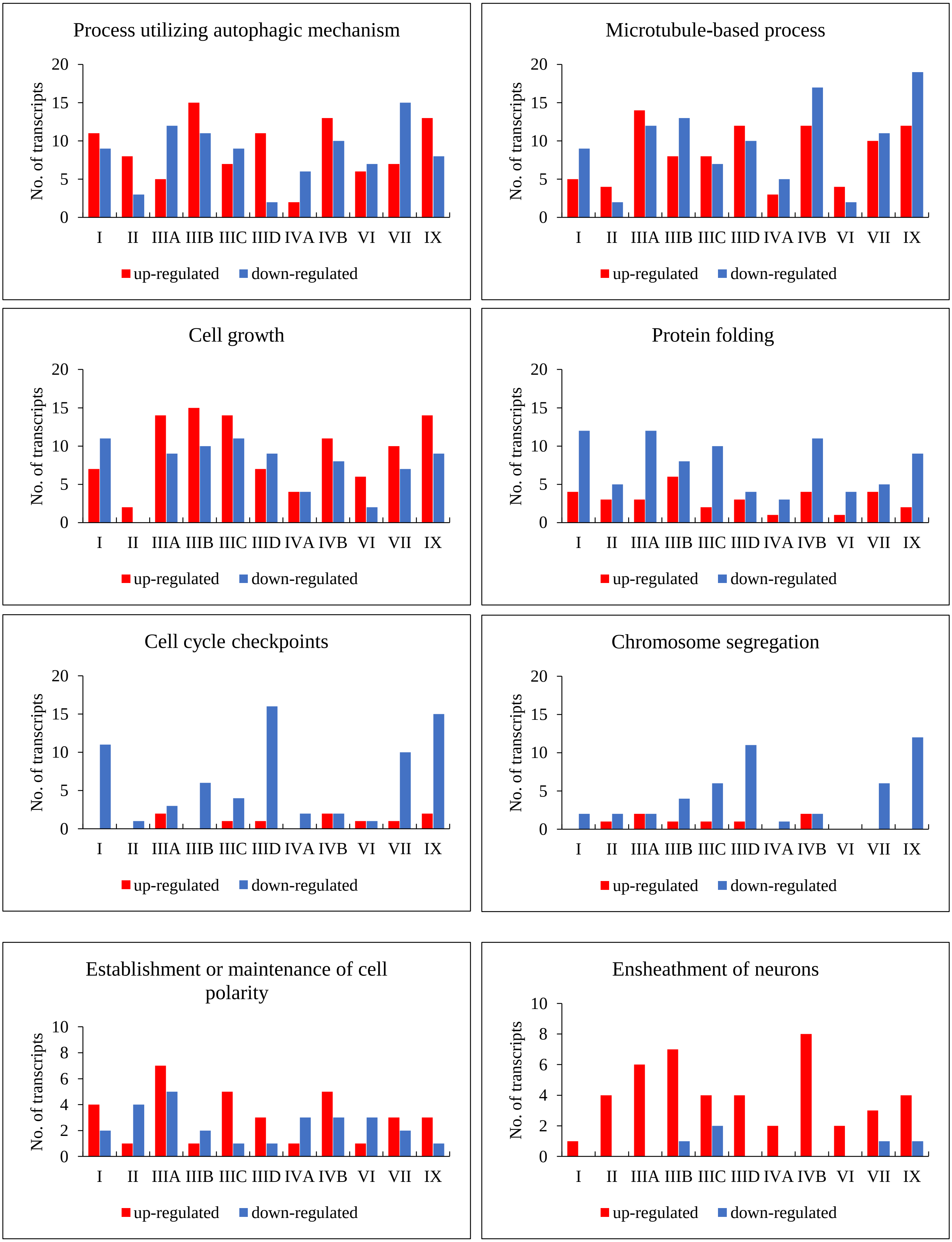

Figure S1 (continued).

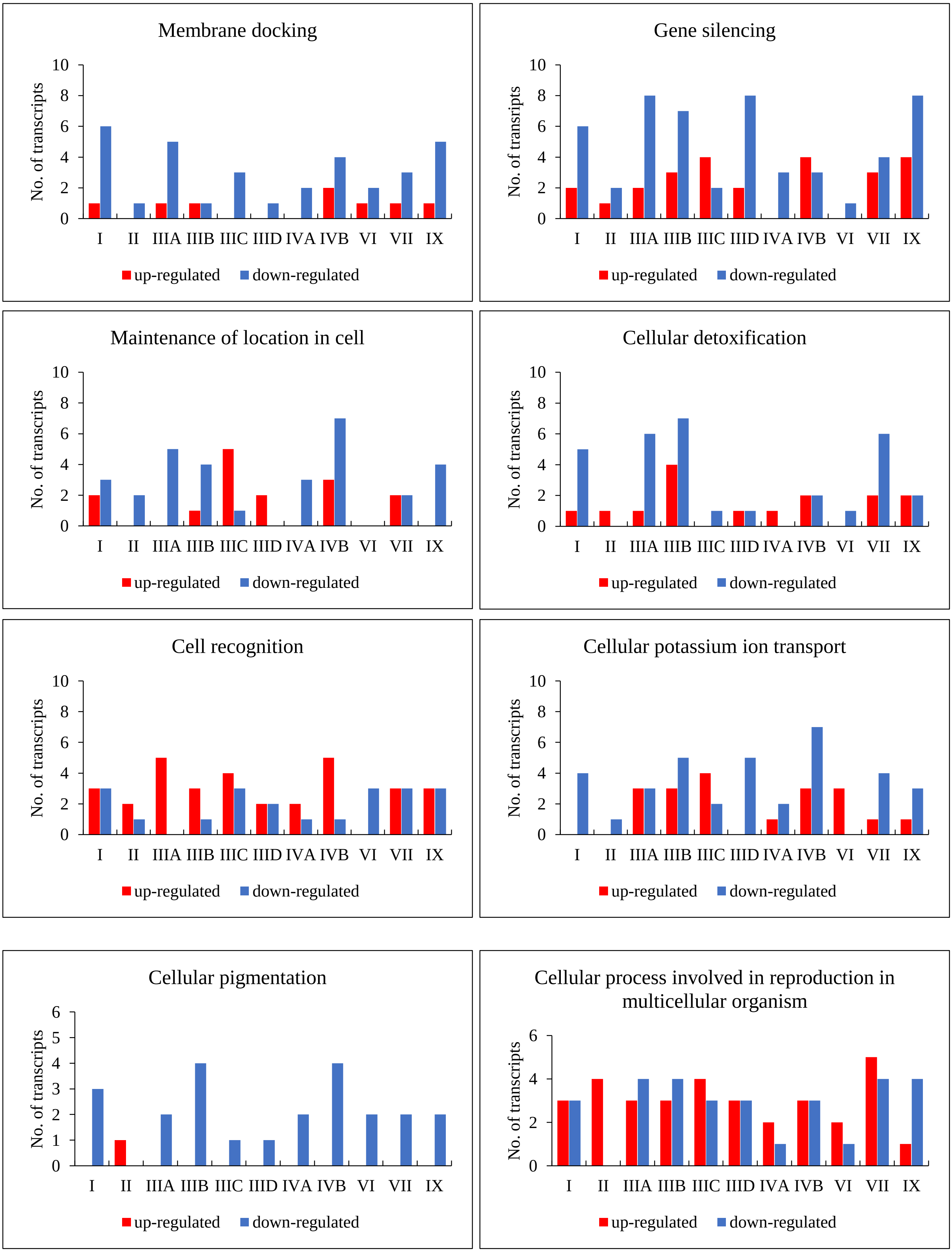

Figure S1 (continued).

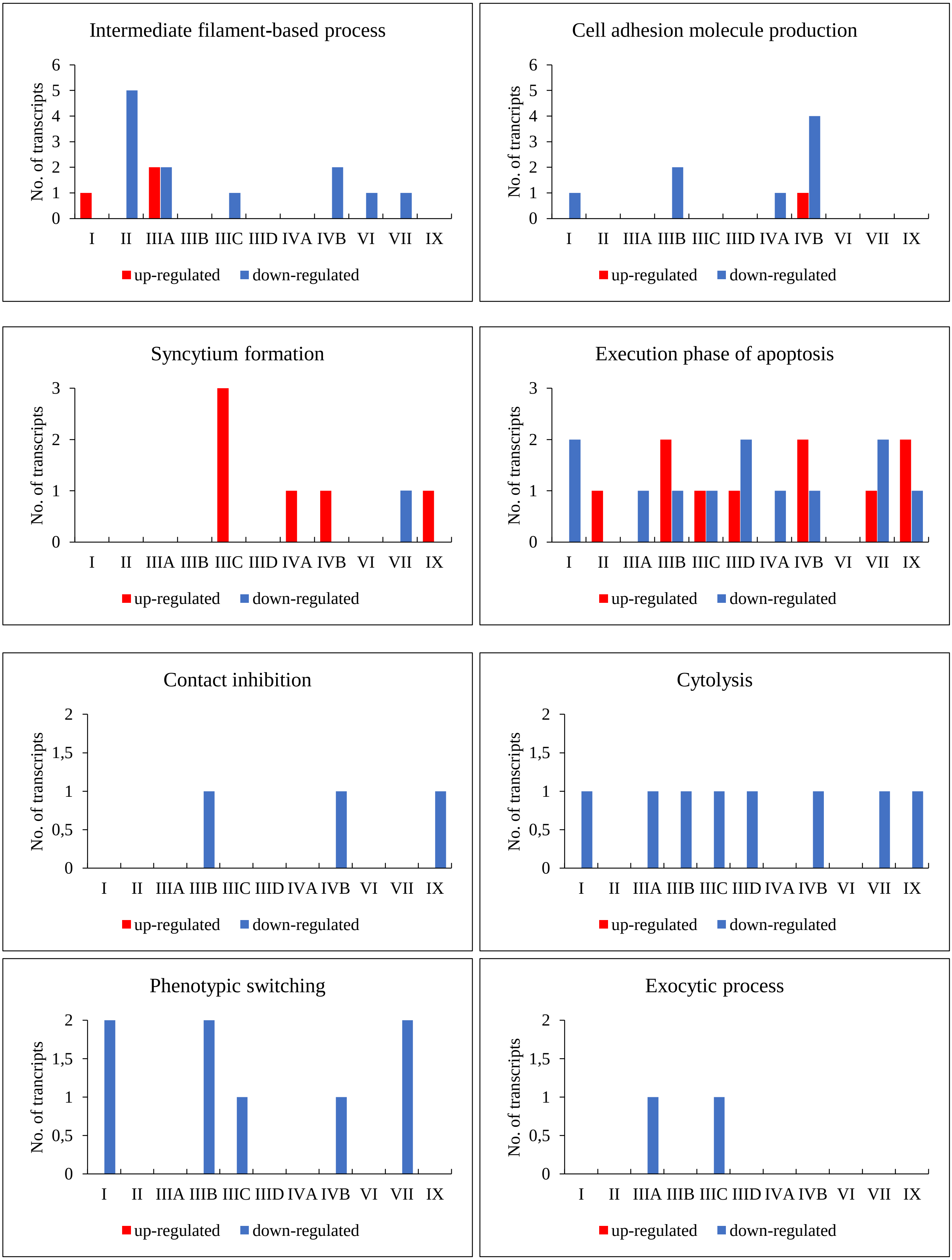

Figure S2. Heat maps indicating transcripts of genes which products are involved in various cellular processes and which expression is significantly changed in different MPS types relative to control HDFa line (S2-1 to S2-49 for particular processes, with FDR<0.1 in panels A, FDR<10^{-6} in panels B, if relevant, and number of the changed transcripts in panels B or C). Because of the complexity of panels, and indication of numbers of biological repetitions (Roman numbers), names of MPS types are indicated atypically by Arabic numbers.

Fig. S2-1

A

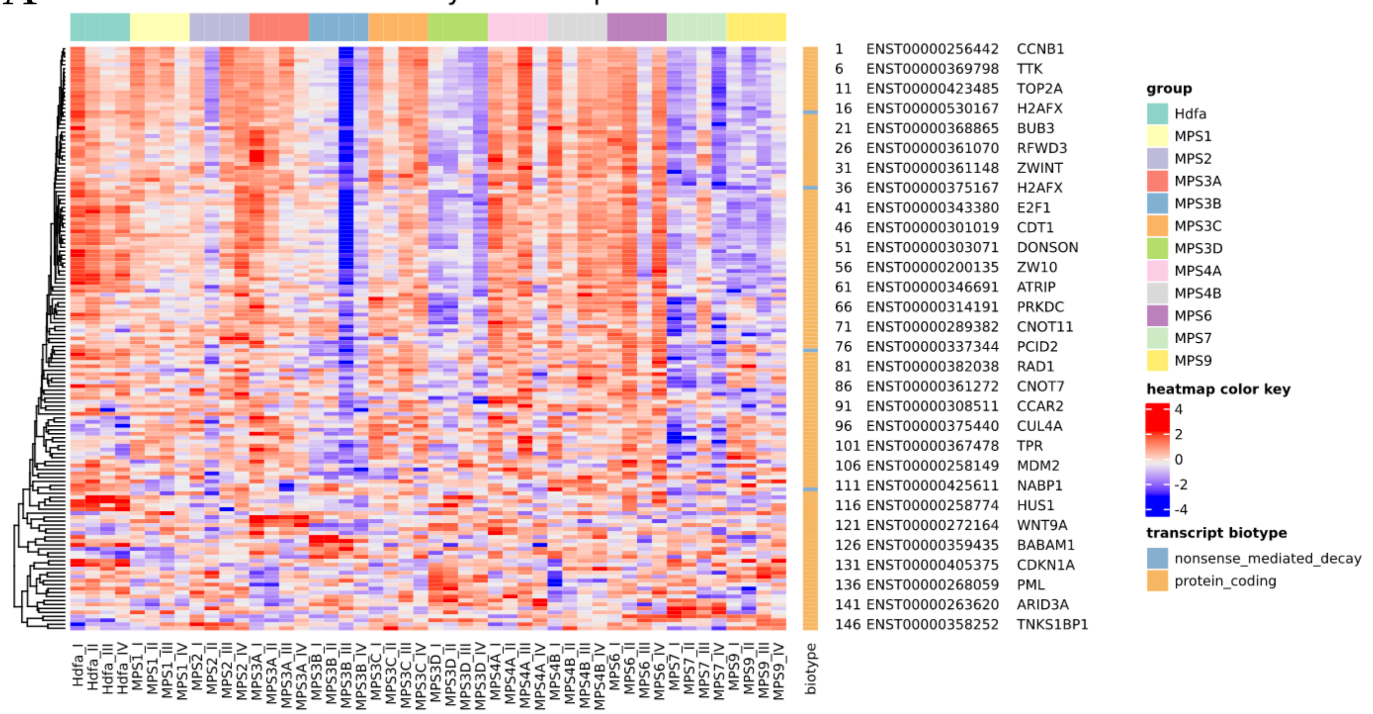

B

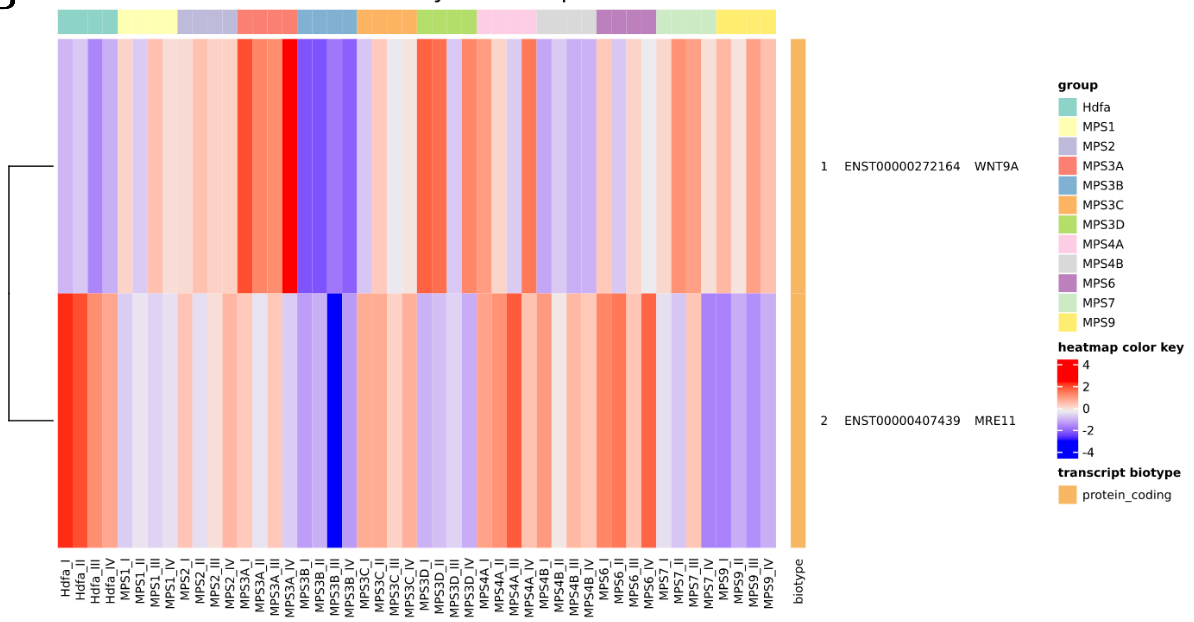

C

Transcripts in **Cell Cycle Checkpoint** process	Significant changes in particular MPS type vs HDFa line										
	I	II	IIIA	IIIB	IIIC	IIID	IVA	IVB	VI	VII	IX
Up-regulated (FDR<0.1)	0	0	2	0	1	1	0	2	1	1	2
Up-regulated (FDR<0.000001)	0	0	1	0	0	0	0	0	0	0	1
Down-regulated (FDR<0.1)	11	1	3	6	4	16	2	2	1	10	15
Down-regulated (FDR<0.000001)	0	0	0	1	0	0	0	0	0	0	1

Fig. S2-2

A

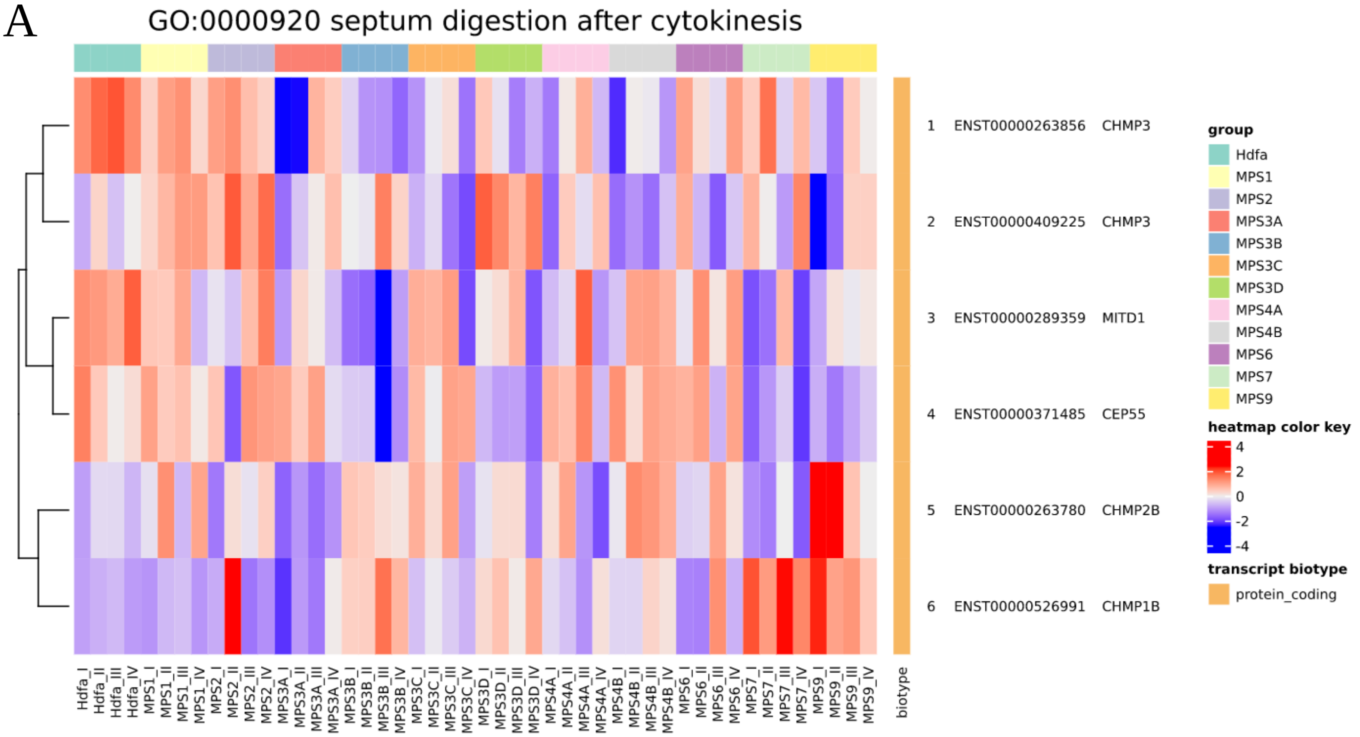

B

Transcripts in **Septum digestion after cytokinesis** process	Significant changes in particular MPS type vs HDFa line										
	I	II	IIIA	IIIB	IIIC	IIID	IVA	IVB	VI	VII	IX
Up-regulated (FDR<0.1)	0	0	0	0	0	0	0	0	0	0	0
Up-regulated (FDR<0.000001)	0	0	0	0	0	0	0	0	0	0	0
Down-regulated (FDR<0.1)	0	0	0	2	0	0	0	0	0	0	0
Down-regulated (FDR<0.000001)	0	0	0	0	0	0	0	0	0	0	0

Fig. S2-3

A

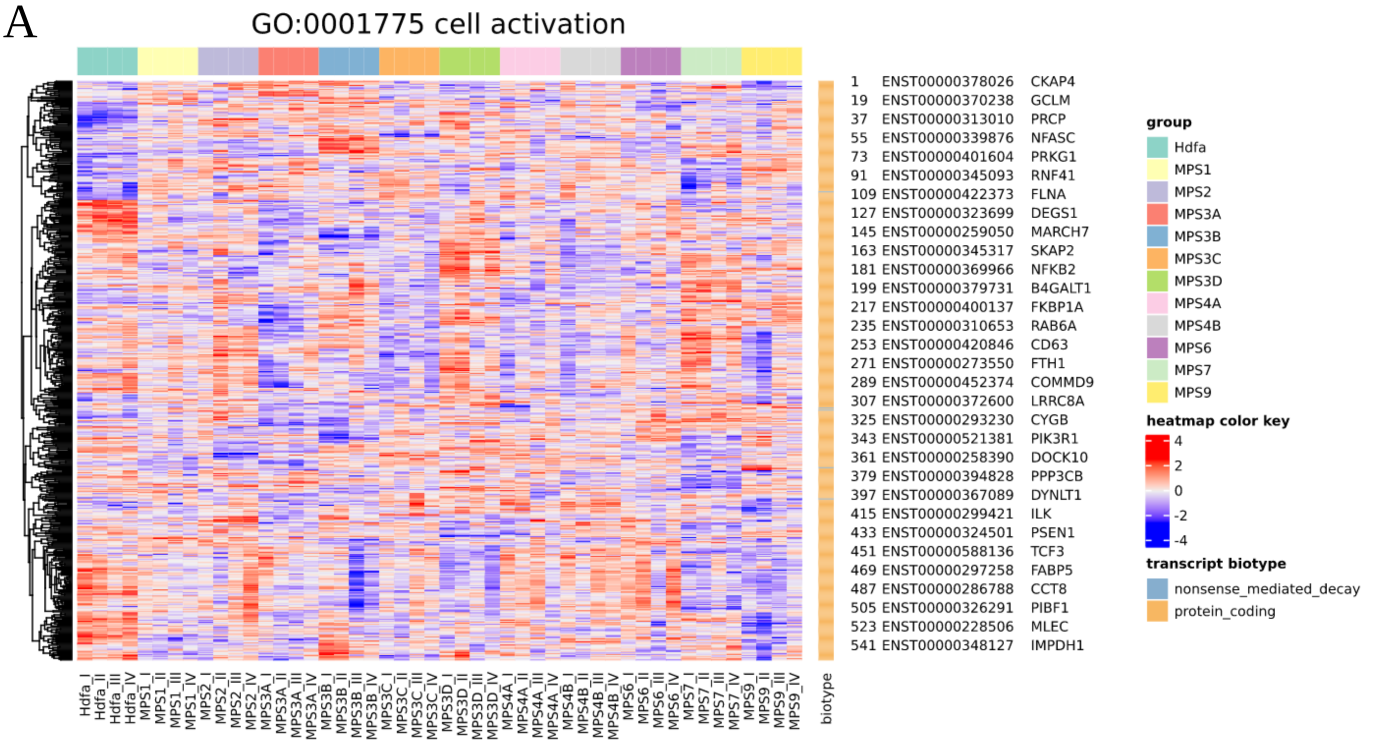

B

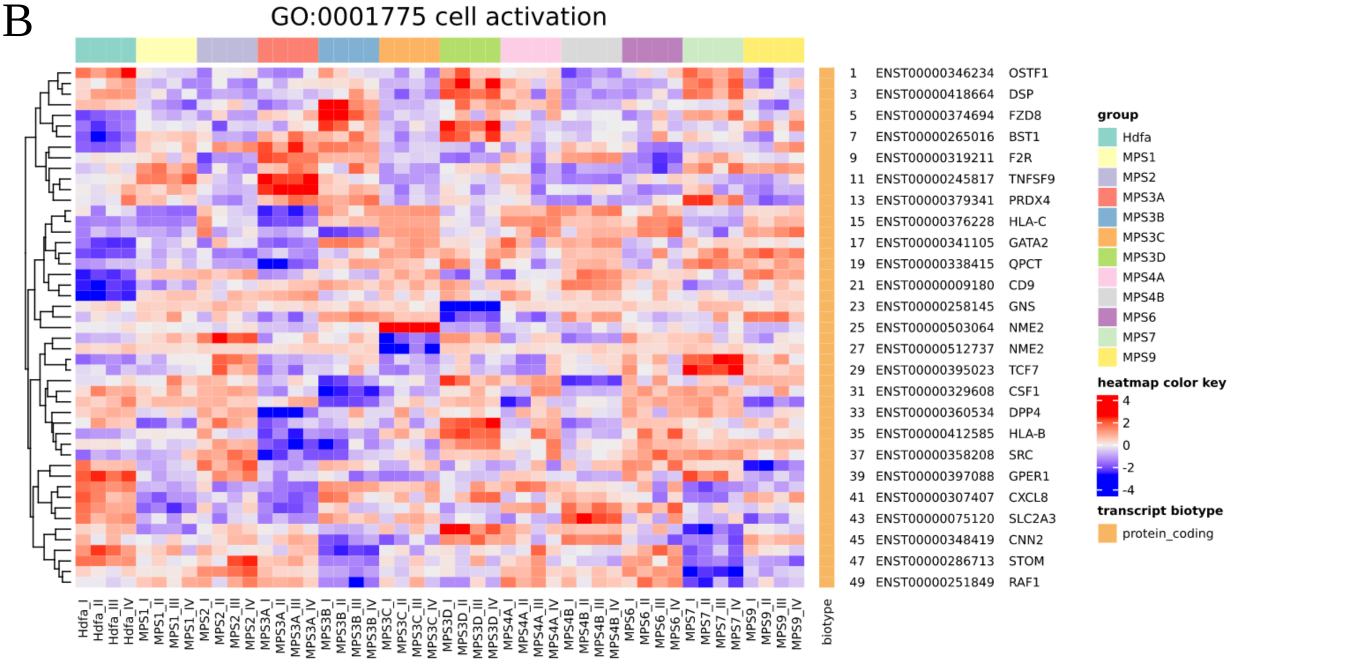

C

Transcripts in **Cell activation** process	Significant changes in particular MPS type vs HDFa line										
	I	II	IIIA	IIIB	IIIC	IIID	IVA	IVB	VI	VII	IX
Up-regulated (FDR<0.1)	18	16	19	33	24	16	8	29	8	25	22
Up-regulated (FDR<0.000001)	7	5	8	9	9	7	3	10	4	9	9
Down-regulated (FDR<0.1)	35	11	38	27	32	18	10	47	11	20	31
Down-regulated (FDR<0.000001)	6	1	6	6	4	5	2	8	0	5	6

Fig. S2-4

A

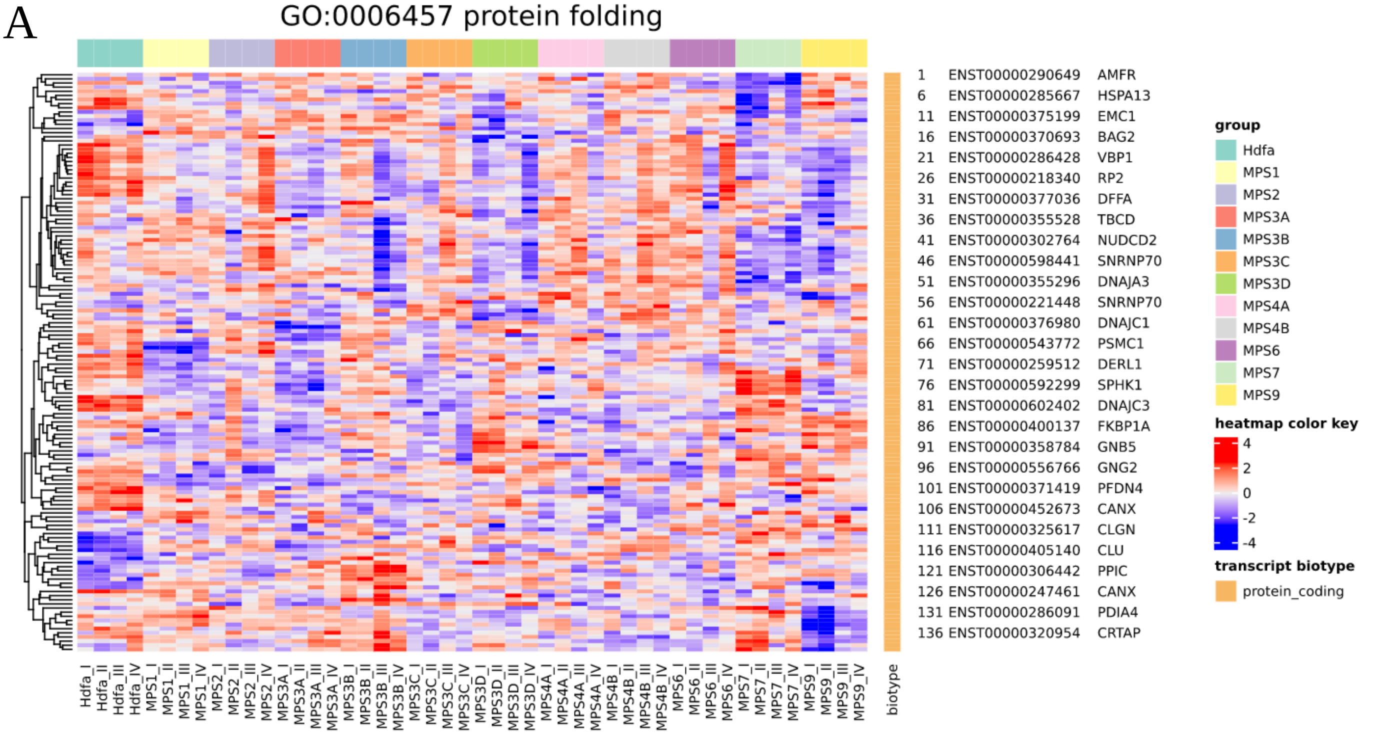

B

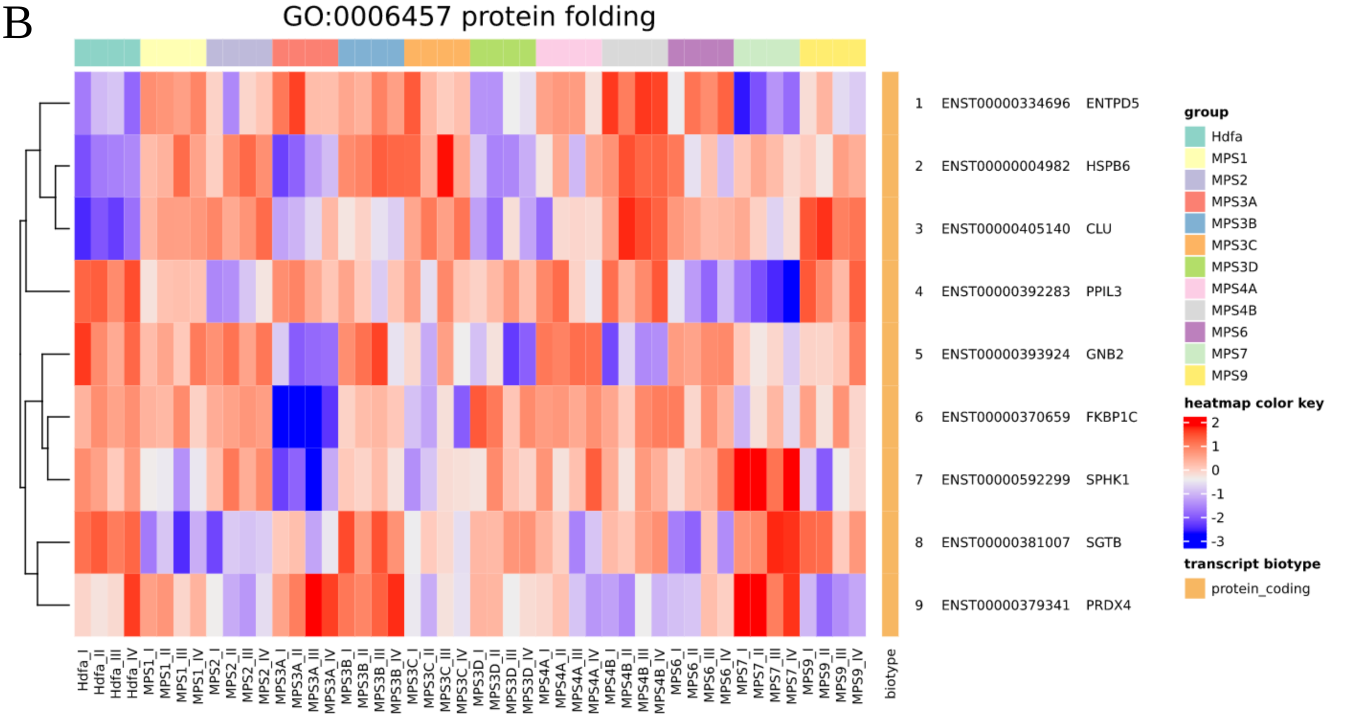

C

Transcripts in **Protein folding** process	Significant changes in particular MPS type vs HDFa line										
	I	II	IIIA	IIIB	IIIC	IIID	IVA	IVB	VI	VII	IX
Up-regulated (FDR<0.1)	4	3	3	6	2	3	1	4	1	4	2
Up-regulated (FDR<0.000001)	2	2	0	3	1	0	0	3	1	2	2
Down-regulated (FDR<0.1)	12	5	12	8	10	4	3	11	4	5	9
Down-regulated (FDR<0.000001)	1	1	2	0	1	0	0	1	1	1	0

Fig. S2-5

A

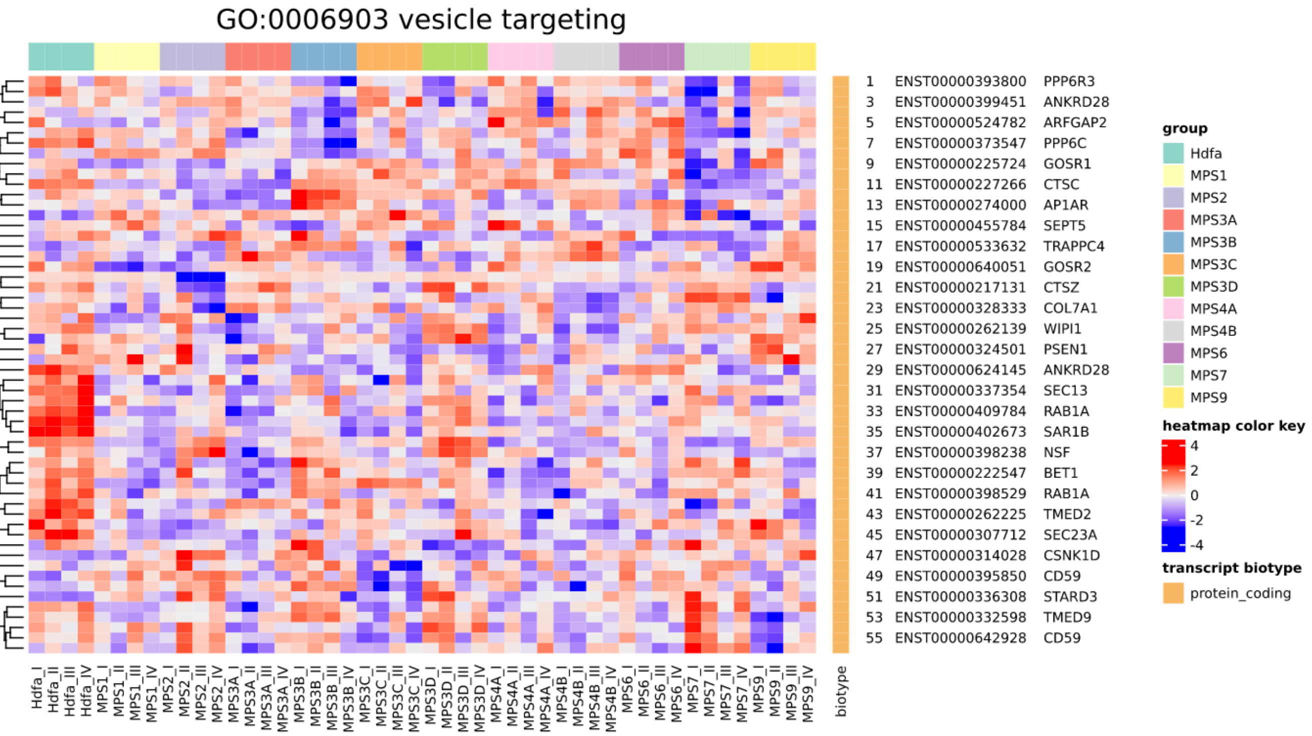

B

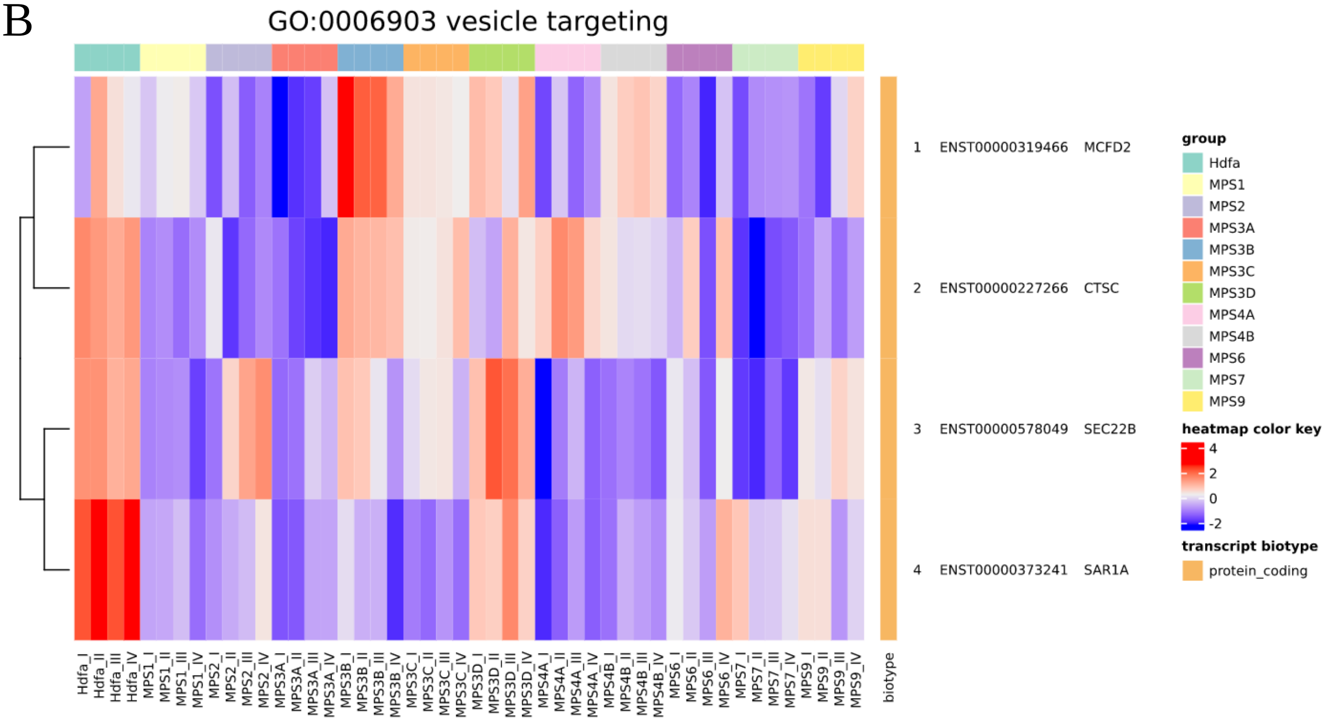

C

Transcripts in **Vesicle targeting** process	Significant changes in particular MPS type vs HDFa line										
	I	II	IIIA	IIIB	IIIC	IIID	IVA	IVB	VI	VII	IX
Up-regulated (FDR<0.1)	0	0	0	2	0	0	0	0	1	0	0
Up-regulated (FDR<0.000001)	0	0	0	0	0	0	0	0	0	0	0
Down-regulated (FDR<0.1)	5	3	7	2	2	1	3	5	3	6	6
Down-regulated (FDR<0.000001)	3	1	2	1	1	0	1	3	0	3	3

Fig. S2-6

A

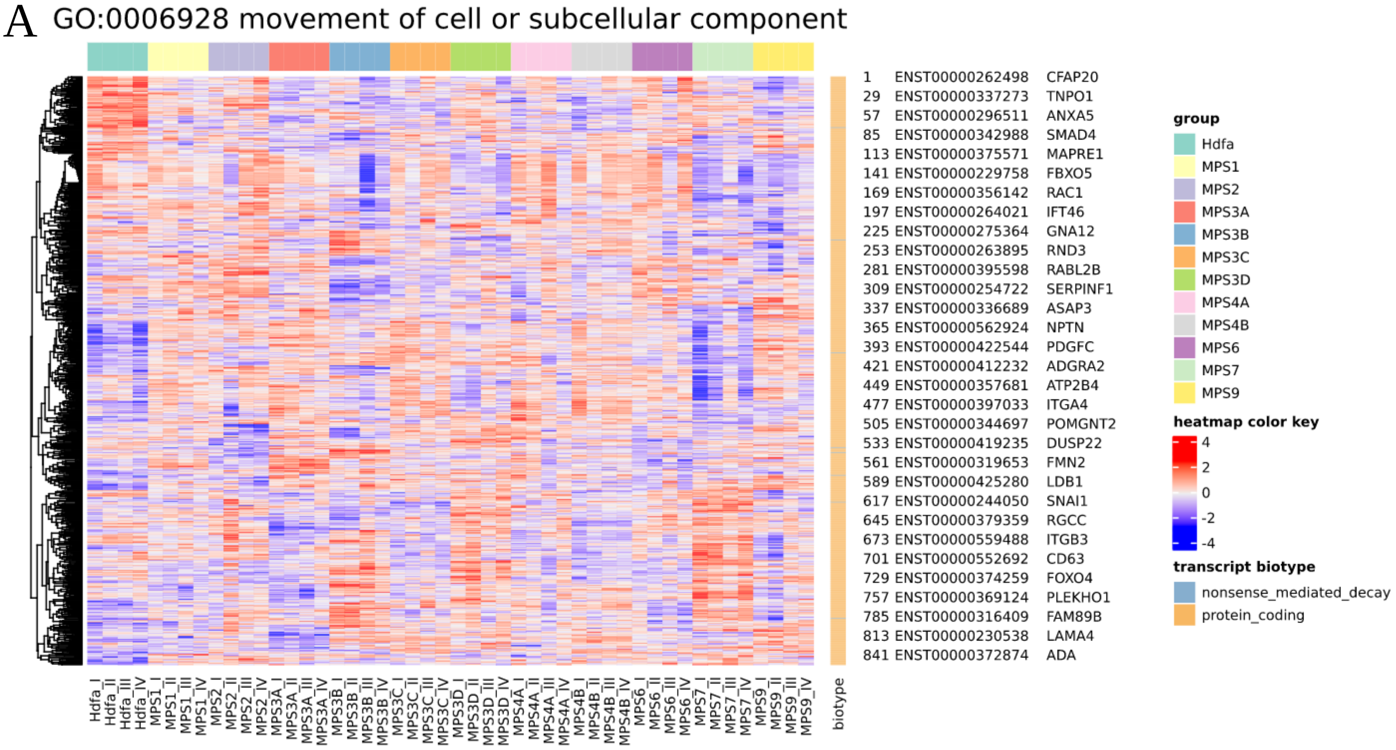

B

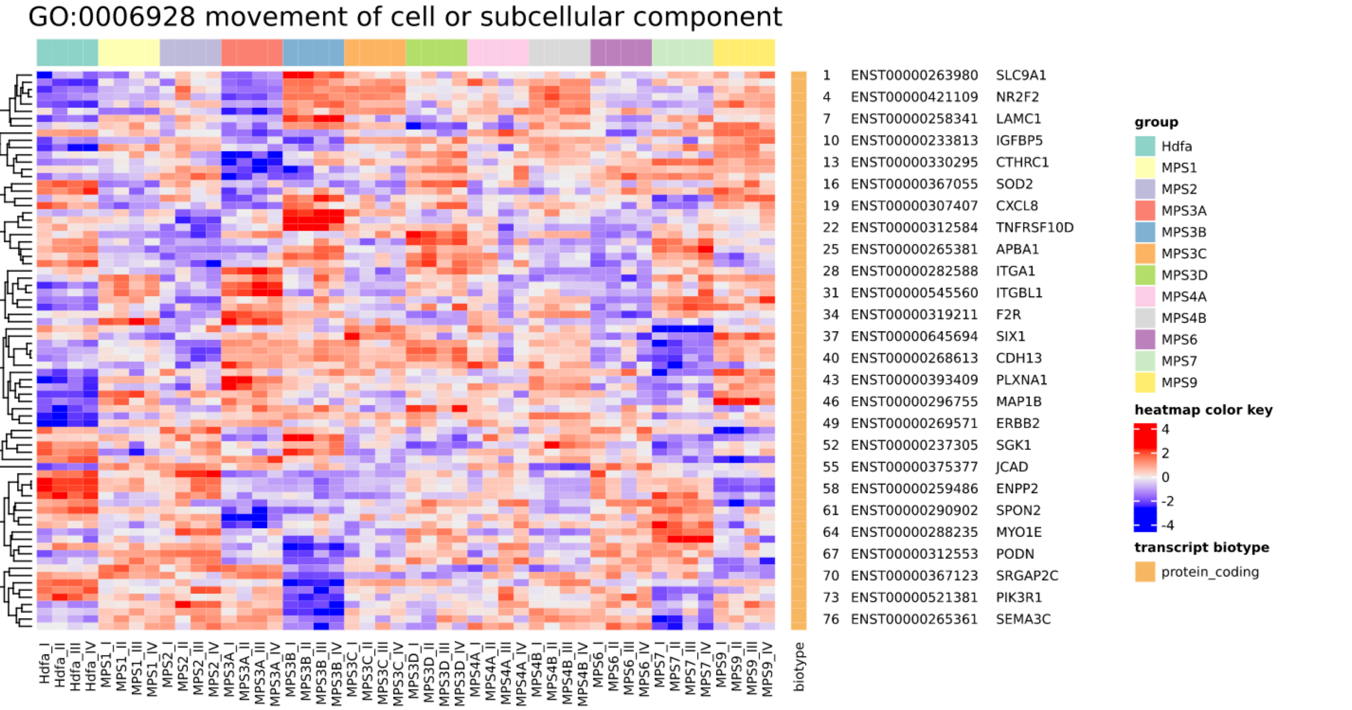

C

Transcripts in **Movement of cel or subcellular component** process	Significant changes in particular MPS type vs HDFa line										
	I	II	IIIA	IIIB	IIIC	IIID	IVA	IVB	VI	VII	IX
Up-regulated (FDR<0.1)	46	22	52	51	46	37	12	50	19	54	51
Up-regulated (FDR<0.000001)	16	8	19	18	11	16	5	17	8	13	19
Down-regulated (FDR<0.1)	35	9	43	49	46	21	20	57	17	40	41
Down-regulated (FDR<0.000001)	10	4	12	12	12	9	4	10	5	13	10

Fig. S2-7

A

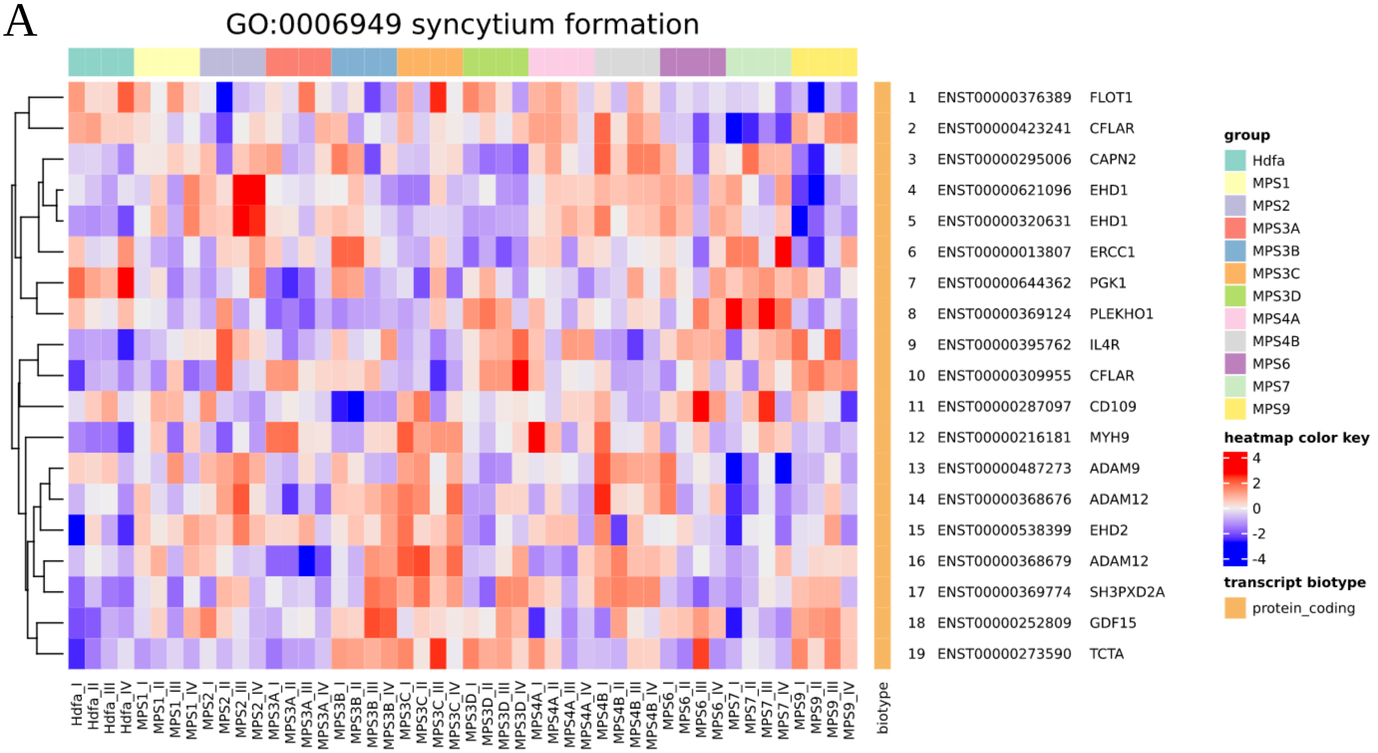

B

Transcripts in **Syncytium formation** process	Significant changes in particular MPS type vs HDFa line										
	I	II	IIIA	IIIB	IIIC	IIID	IVA	IVB	VI	VII	IX
Up-regulated (FDR<0.1)	0	0	0	0	3	0	1	1	0	0	0
Up-regulated (FDR<0.000001)	0	0	0	0	0	0	0	0	0	0	0
Down-regulated (FDR<0.1)	0	0	0	0	0	0	0	0	0	1	0
Down-regulated (FDR<0.000001)	0	0	0	0	0	0	0	0	0	0	0

Fig. S2-8

A

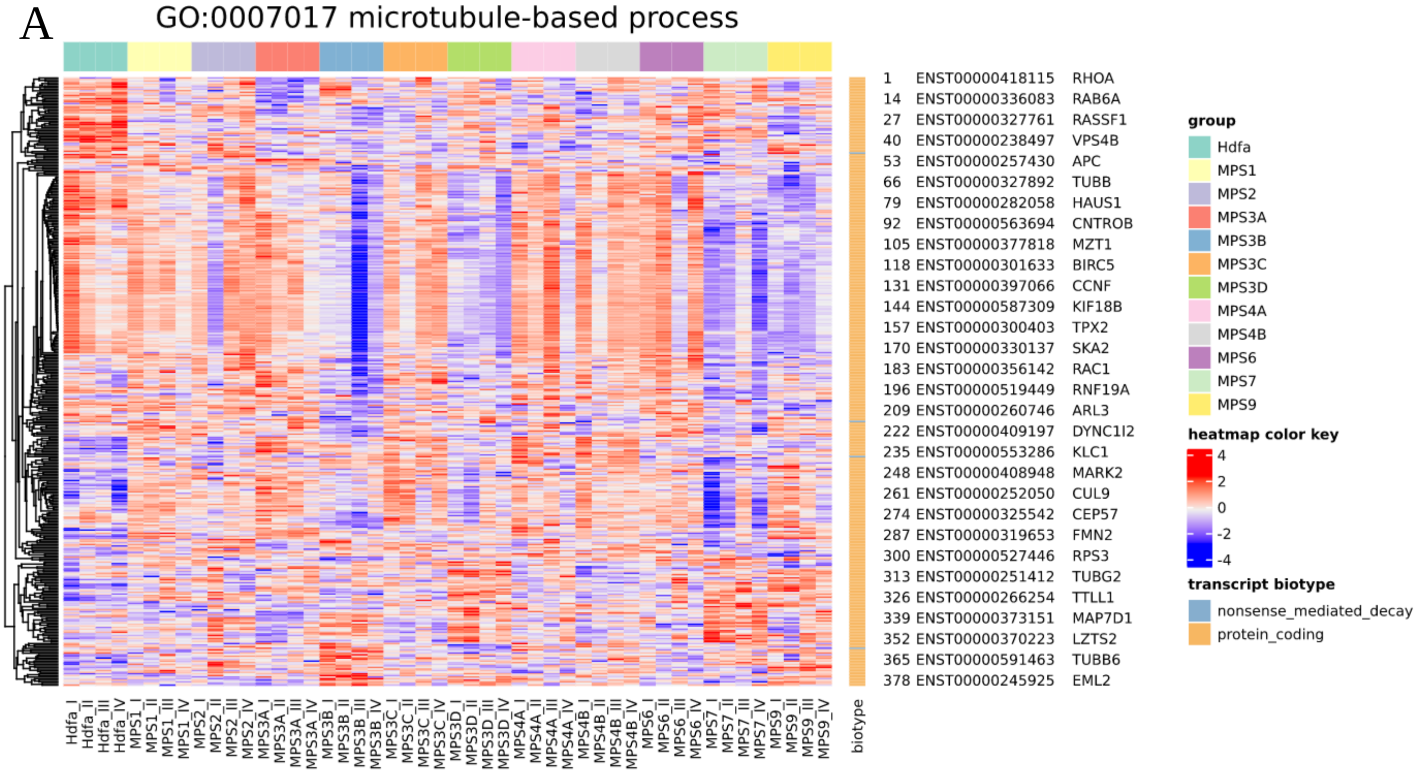

B

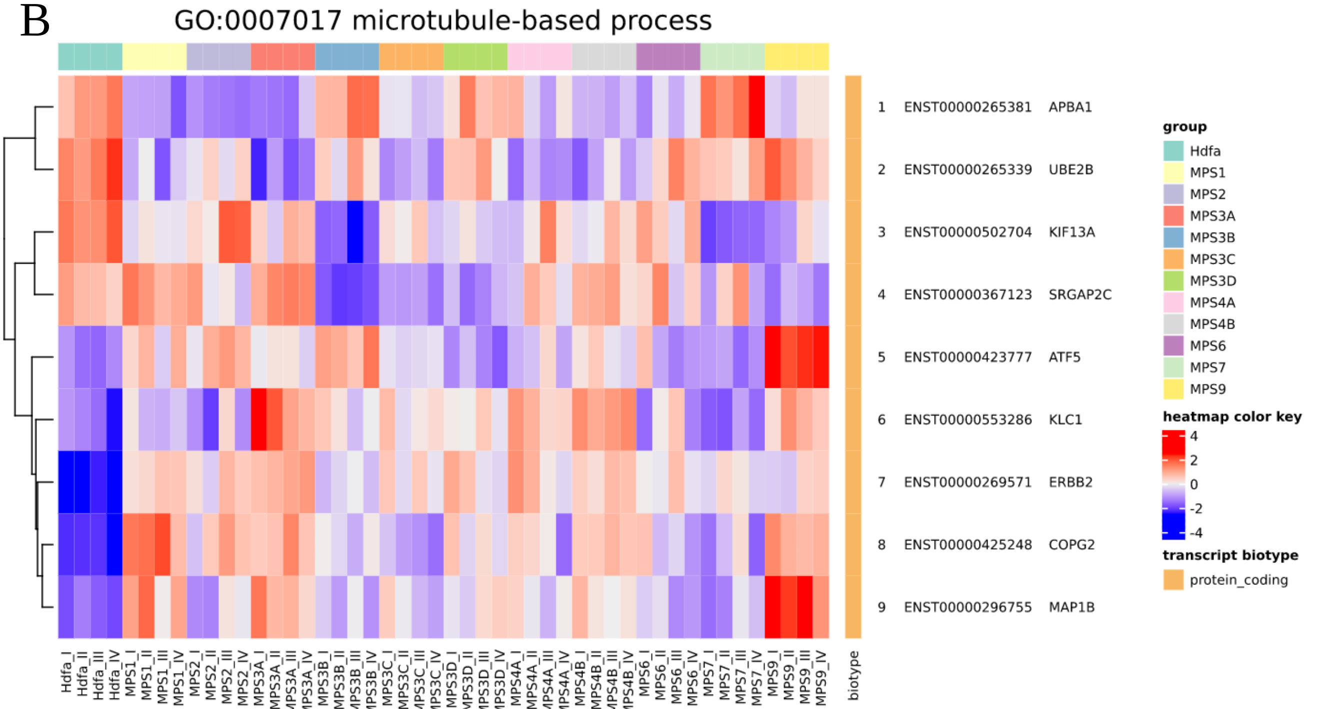

C

Transcripts in **Microtubule-based process** process	Significant changes in particular MPS type vs HDFa line										
	I	II	IIIA	IIIB	IIIC	IIID	IVA	IVB	VI	VII	IX
Up-regulated (FDR<0.1)	5	4	14	8	8	12	3	12	4	10	12
Up-regulated (FDR<0.000001)	1	0	4	2	0	2	1	2	0	0	4
Down-regulated (FDR<0.1)	9	2	12	13	7	10	5	17	2	11	19
Down-regulated (FDR<0.000001)	2	1	2	2	3	2	1	1	0	1	1

Fig. S2-9

A

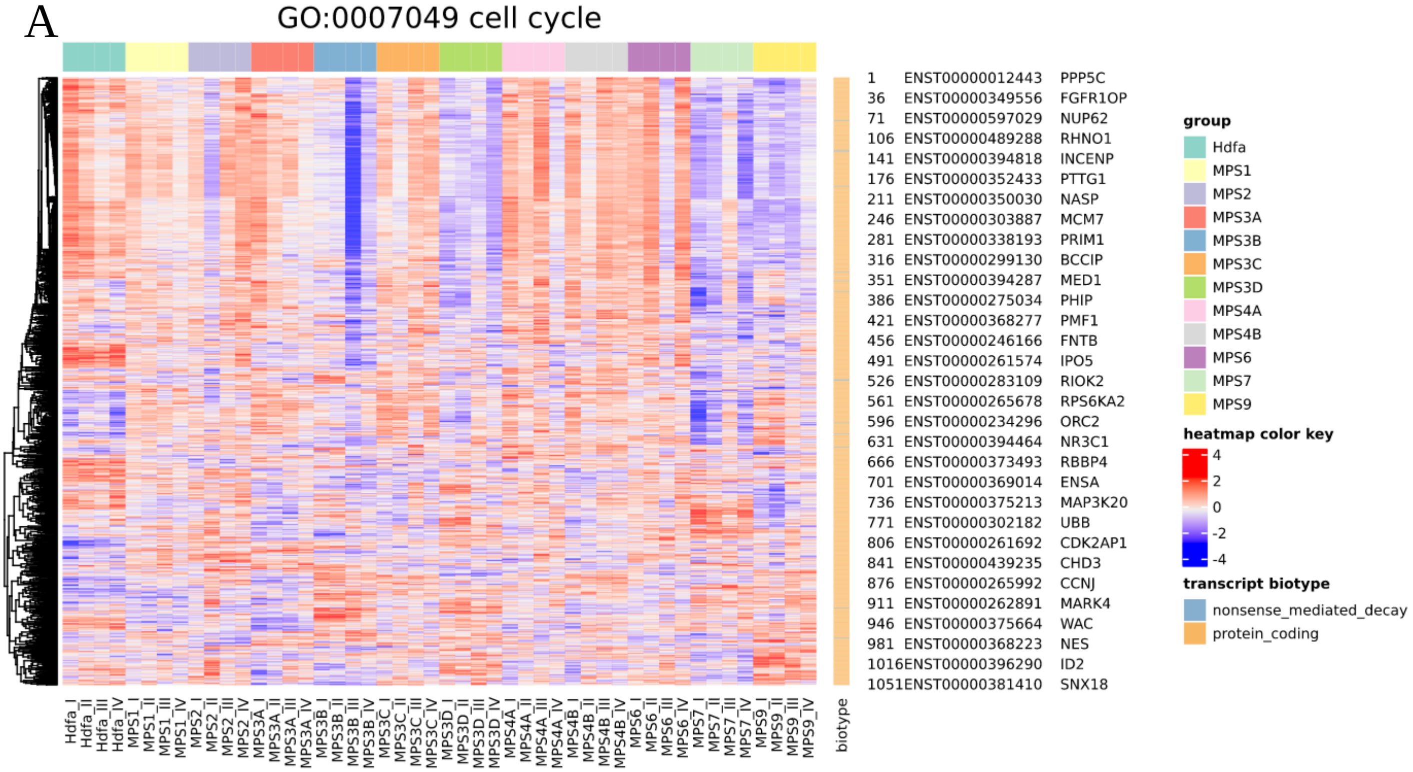

B

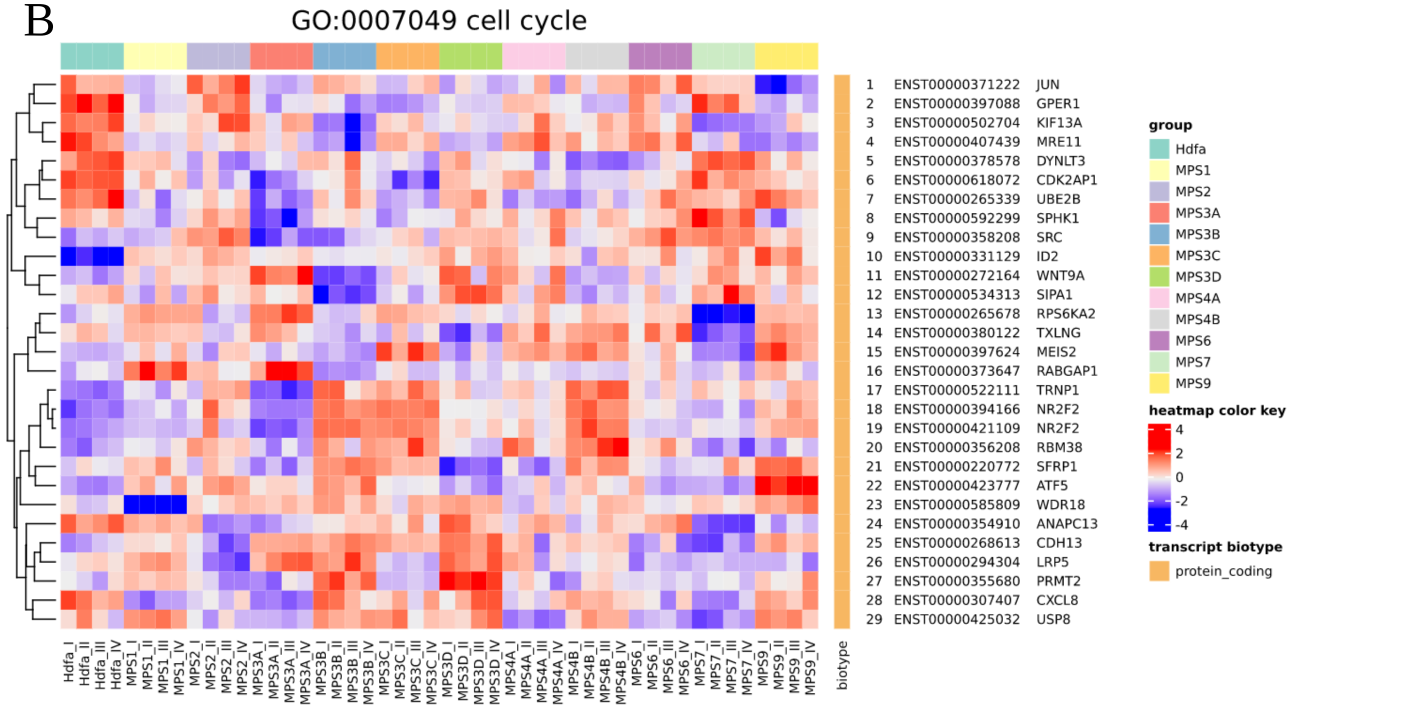

C

Transcripts in **Cell Cycle** process	Significant changes in particular MPS type vs HDFa line										
	I	II	IIIA	IIIB	IIIC	IIID	IVA	IVB	VI	VII	IX
Up-regulated (FDR<0.1)	11	13	21	22	29	20	13	33	12	26	24
Up-regulated (FDR<0.000001)	0	2	3	5	4	4	4	5	3	3	5
Down-regulated (FDR<0.1)	41	10	40	33	30	79	12	32	8	53	83
Down-regulated (FDR<0.000001)	4	0	4	3	3	4	2	3	0	5	4

Fig. S2-10

A

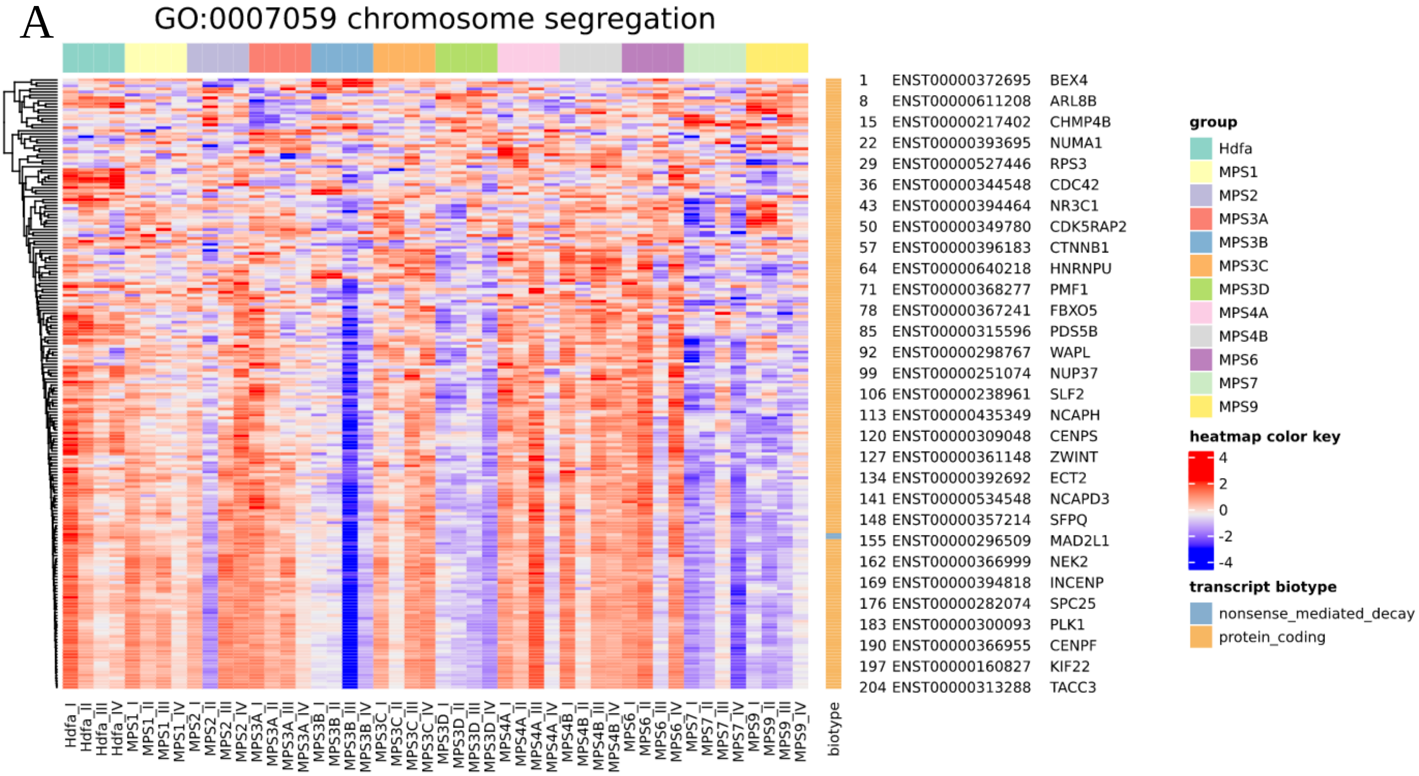

B

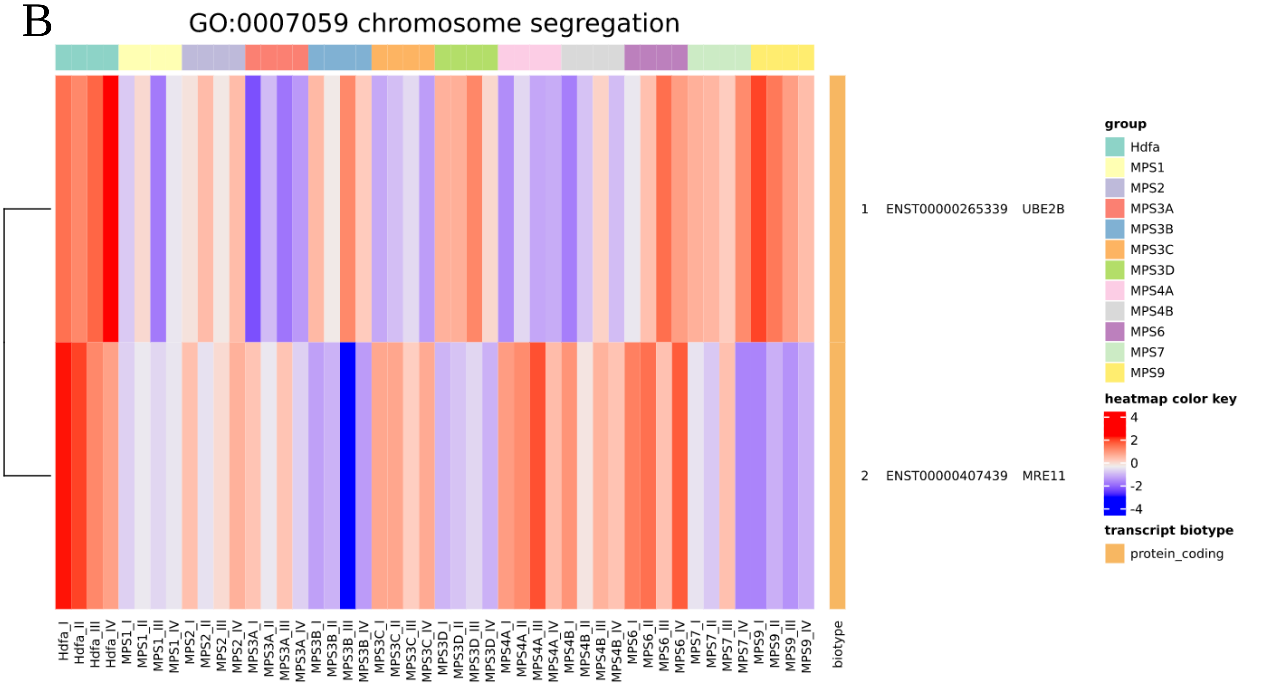

C

Transcripts in **Chromosome segregation** process	Significant changes in particular MPS type vs HDFa line										
	I	II	IIIA	IIIB	IIIC	IIID	IVA	IVB	VI	VII	IX
Up-regulated (FDR<0.1)	0	1	2	1	1	1	0	2	0	0	0
Up-regulated (FDR<0.000001)	0	0	0	0	0	0	0	0	0	0	0
Down-regulated (FDR<0.1)	2	2	2	4	6	11	1	2	0	6	12
Down-regulated (FDR<0.000001)	0	0	1	1	1	0	1	0	0	0	1

Fig. S2-11

A

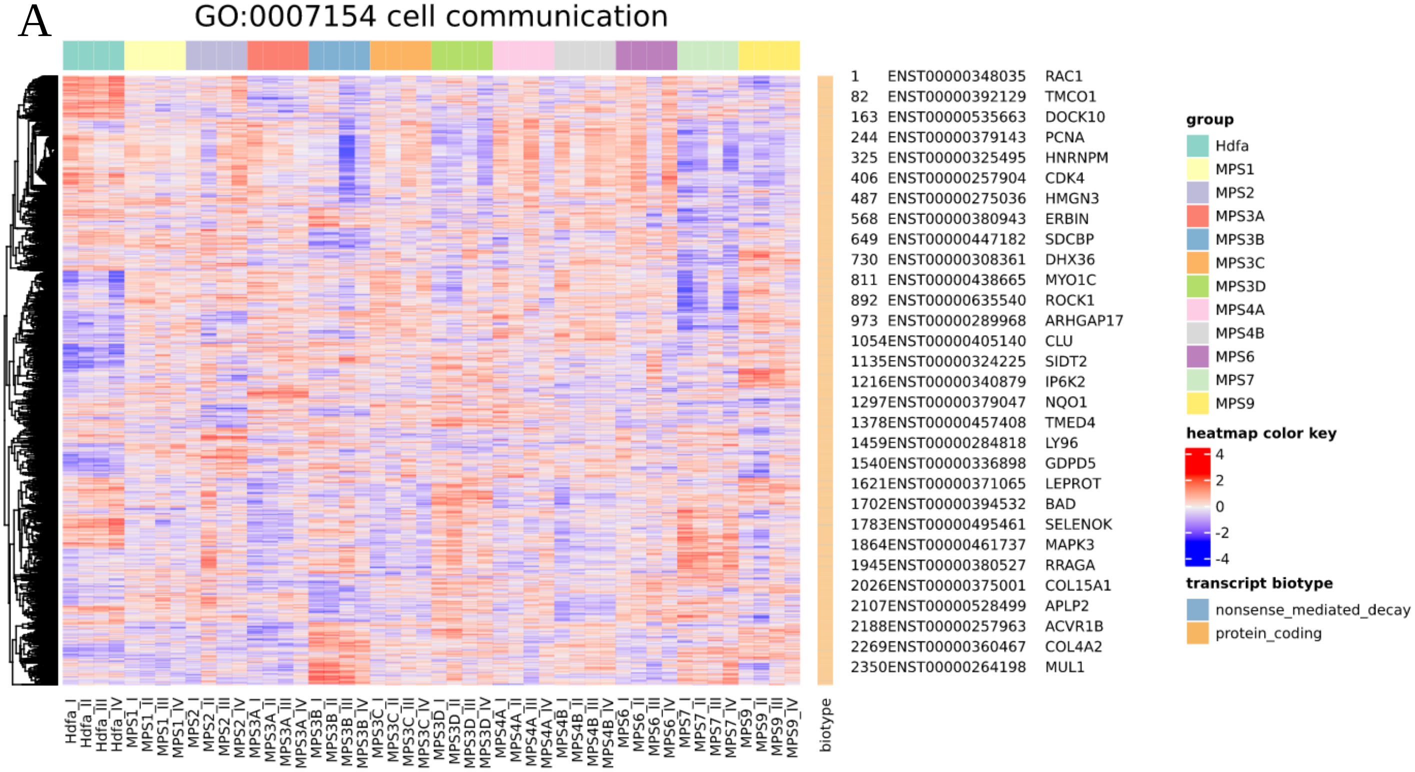

B

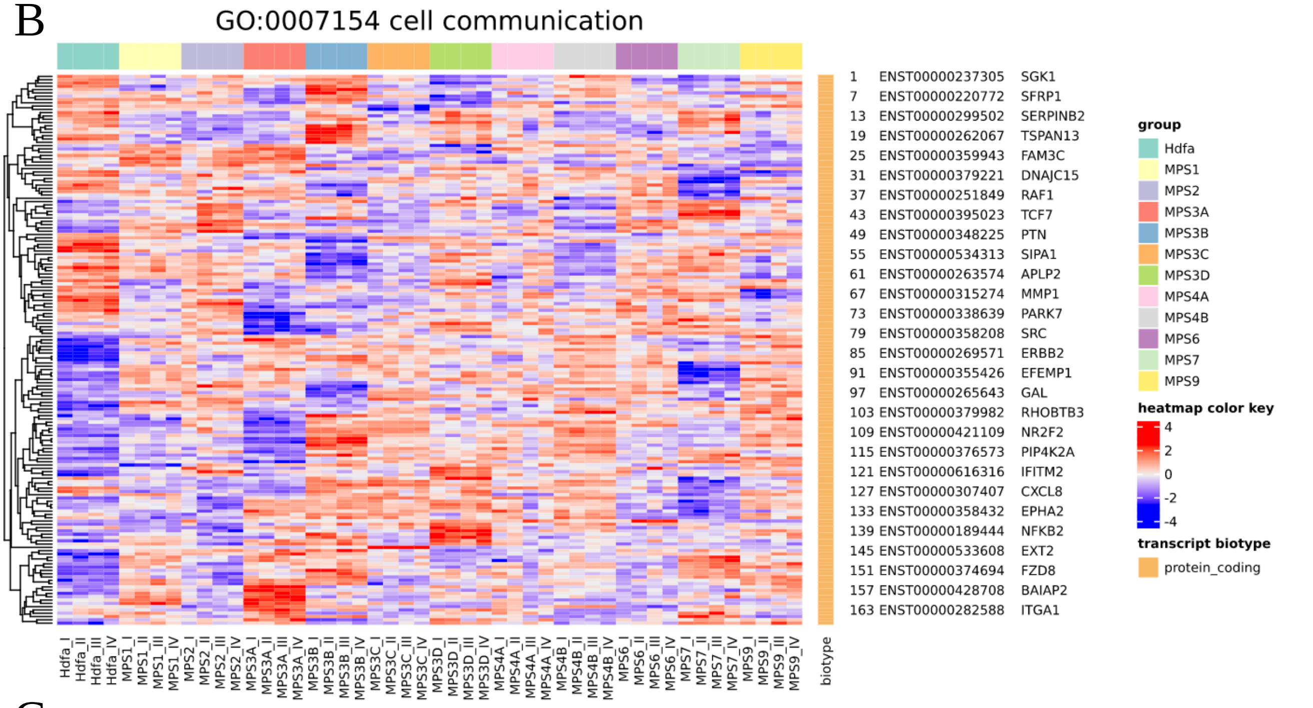

C

Transcripts in **Cell Communication** process	Significant changes in particular MPS type vs HDFa line										
	I	II	IIIA	IIIB	IIIC	IIID	IVA	IVB	VI	VII	IX
Up-regulated (FDR<0.1)	98	59	120	135	102	100	34	139	50	119	127
Up-regulated (FDR<0.000001)	36	20	42	40	33	35	12	44	21	30	42
Down-regulated (FDR<0.1)	113	37	146	111	103	78	42	137	41	115	113
Down-regulated (FDR<0.000001)	20	13	26	23	22	14	9	28	10	26	18

Fig. S2-12

GO:0007163 establishment or maintenance of cell polarity

A

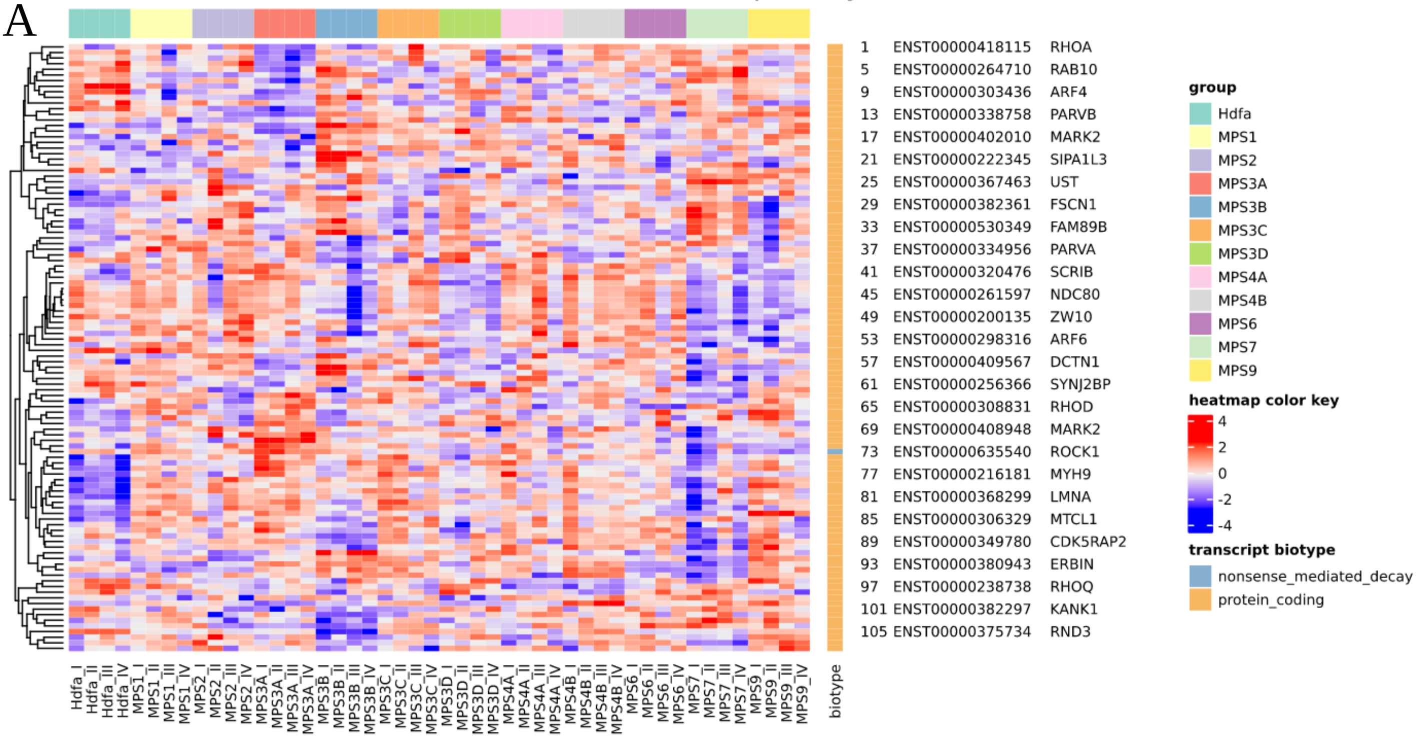

B GO:0007163 establishment or maintenance of cell polarity

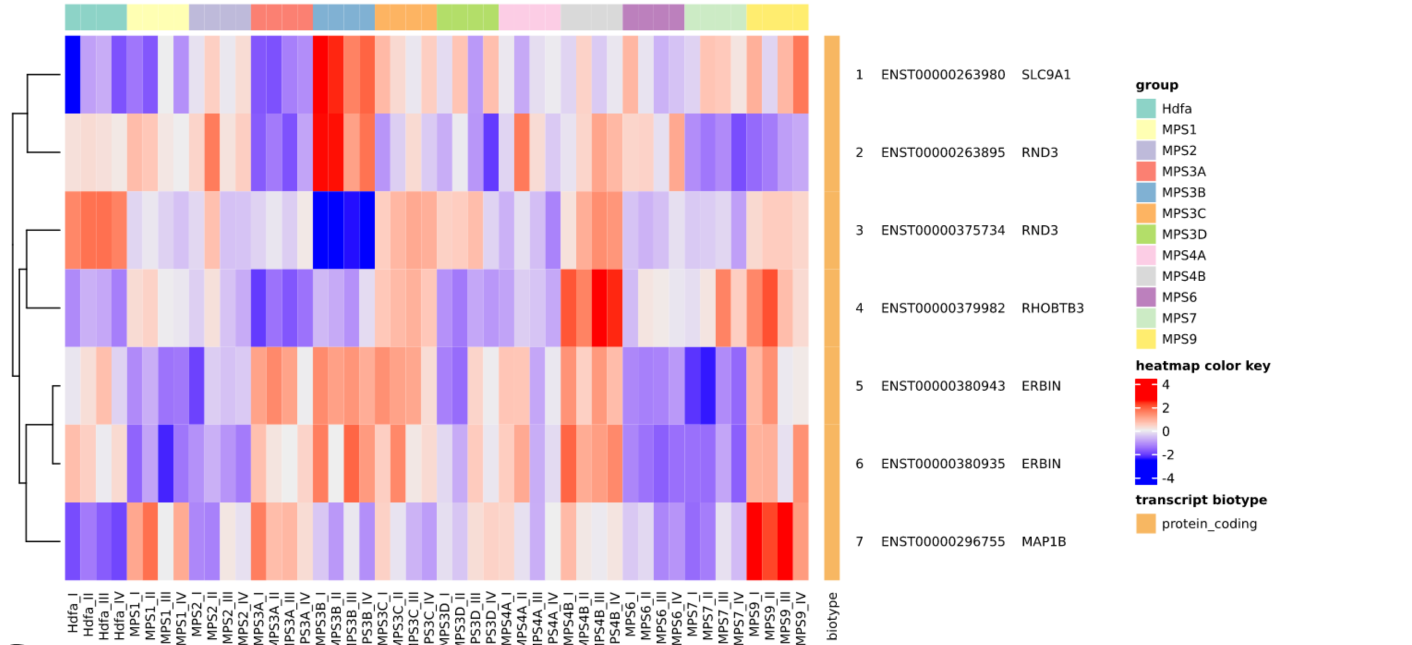

C

Transcripts in **Establishment or maintenance of cel polarity** process	Significant changes in particular MPS type vs HDFa line										
	I	II	IIIA	IIIB	IIIC	IIID	IVA	IVB	VI	VII	IX
Up-regulated (FDR<0.1)	4	1	7	1	5	3	1	5	1	3	3
Up-regulated (FDR<0.000001)	1	0	1	1	1	1	0	2	0	0	1
Down-regulated (FDR<0.1)	2	4	5	2	1	1	3	3	3	2	1
Down-regulated (FDR<0.000001)	1	1	1	1	0	0	1	0	2	2	1

Fig. S2-13

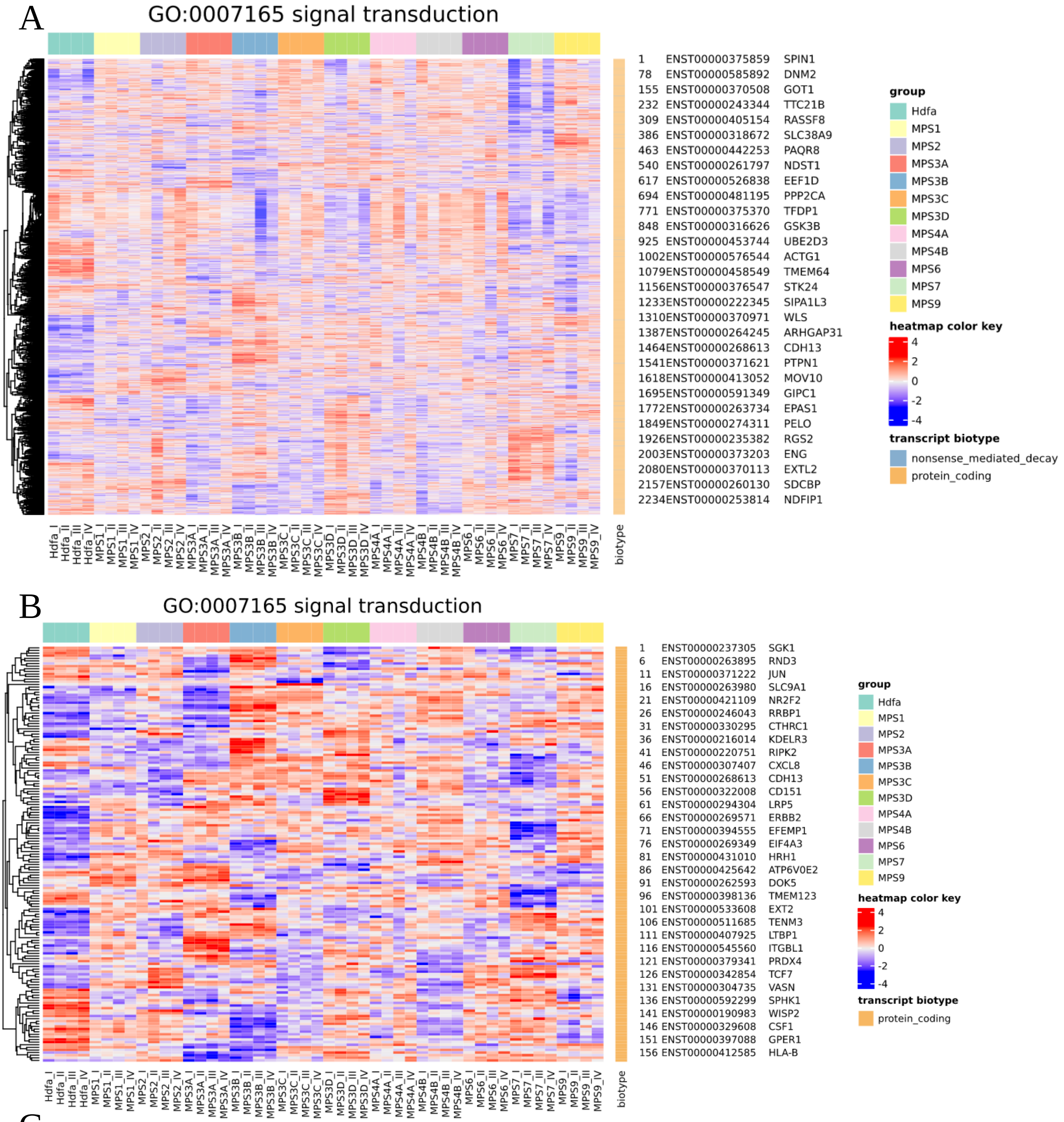

C

Transcripts in **Signal transduction** process	Significant changes in particular MPS type vs HDFa line										
	I	II	IIIA	IIIB	IIIC	IIID	IVA	IVB	VI	VII	IX
Up-regulated (FDR<0.1)	92	58	110	132	98	95	34	134	47	116	125
Up-regulated (FDR<0.000001)	33	20	38	37	32	32	12	42	19	29	41
Down-regulated (FDR<0.1)	112	33	141	108	101	74	43	133	43	112	110
Down-regulated (FDR<0.000001)	20	10	27	23	21	15	9	26	10	25	18

Fig. S2-14

A

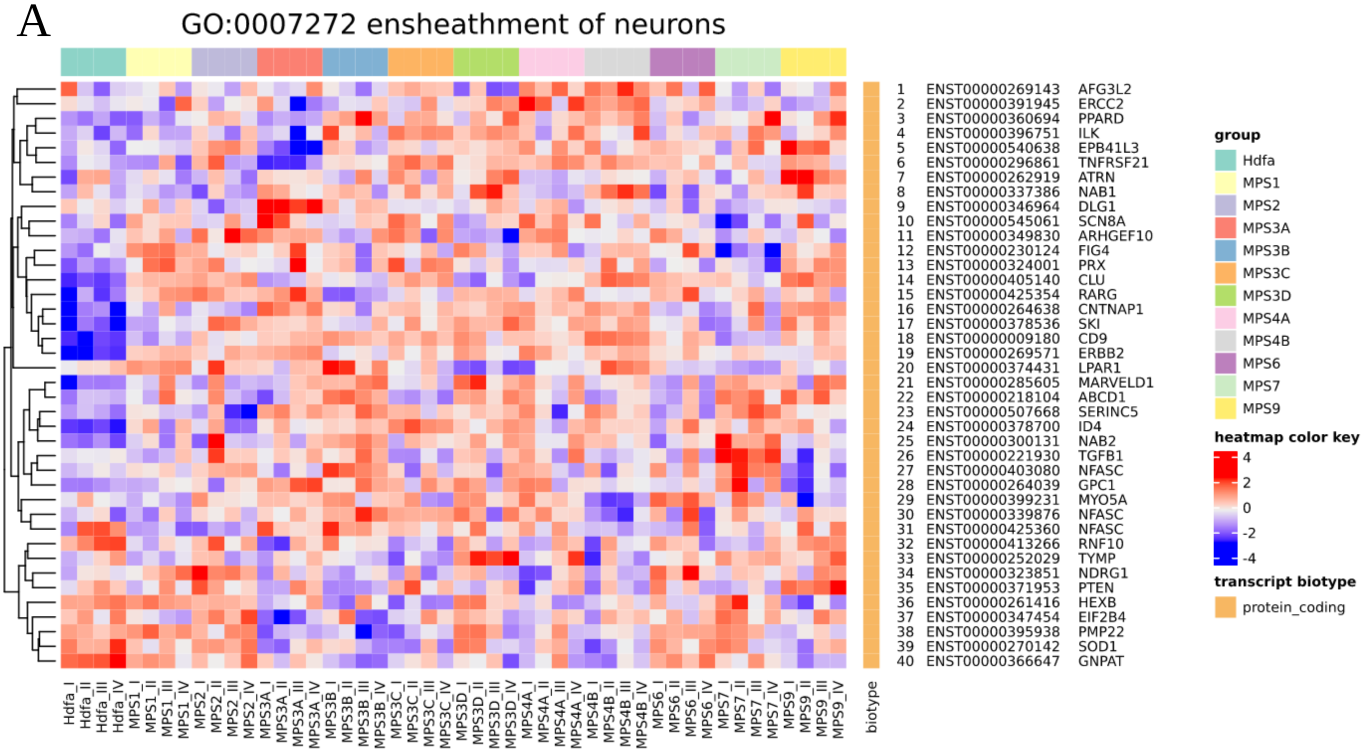

B

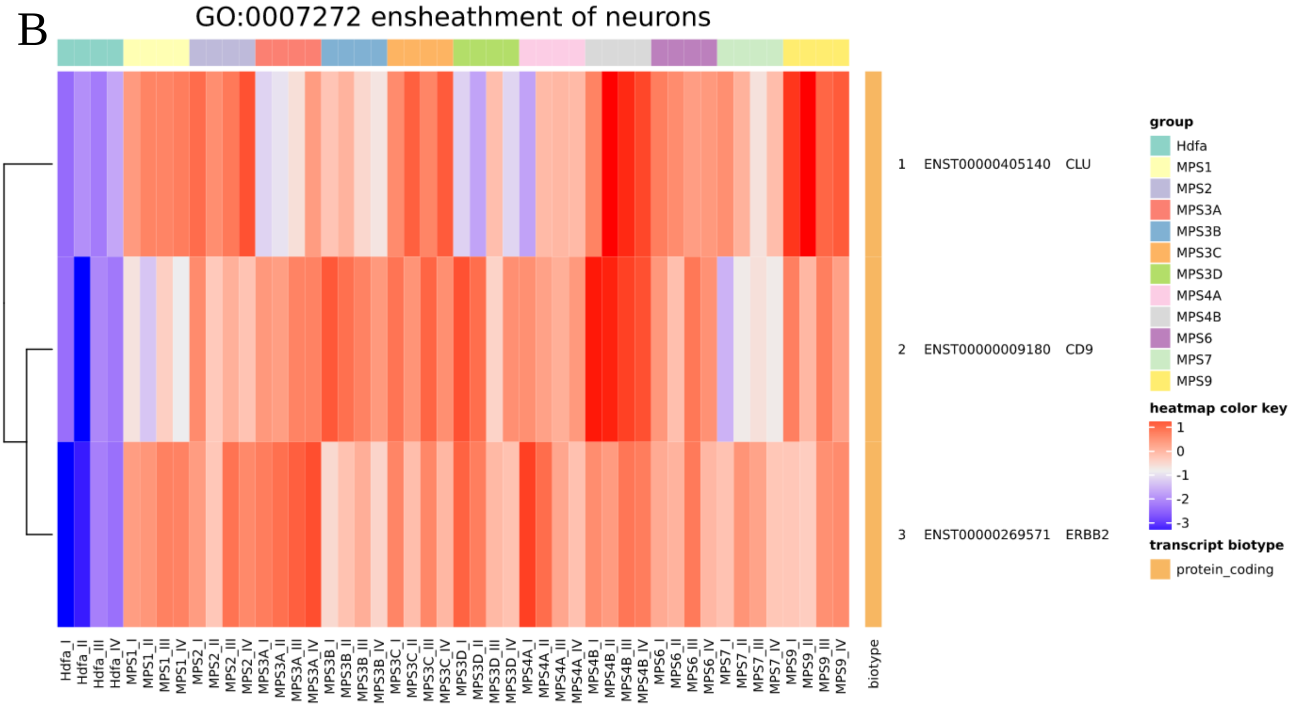

C

Transcripts in **Ensheathment of neurons** process	Significant changes in particular MPS type vs HDFa line										
	I	II	IIIA	IIIB	IIIC	IIID	IVA	IVB	VI	VII	IX
Up-regulated (FDR<0.1)	1	4	6	7	4	4	2	8	2	3	4
Up-regulated (FDR<0.000001)	1	2	1	2	2	1	1	2	2	1	2
Down-regulated (FDR<0.1)	0	0	0	1	2	0	0	0	0	1	1
Down-regulated (FDR<0.000001)	0	0	0	0	0	0	0	0	0	0	0

Fig. S2-15

A

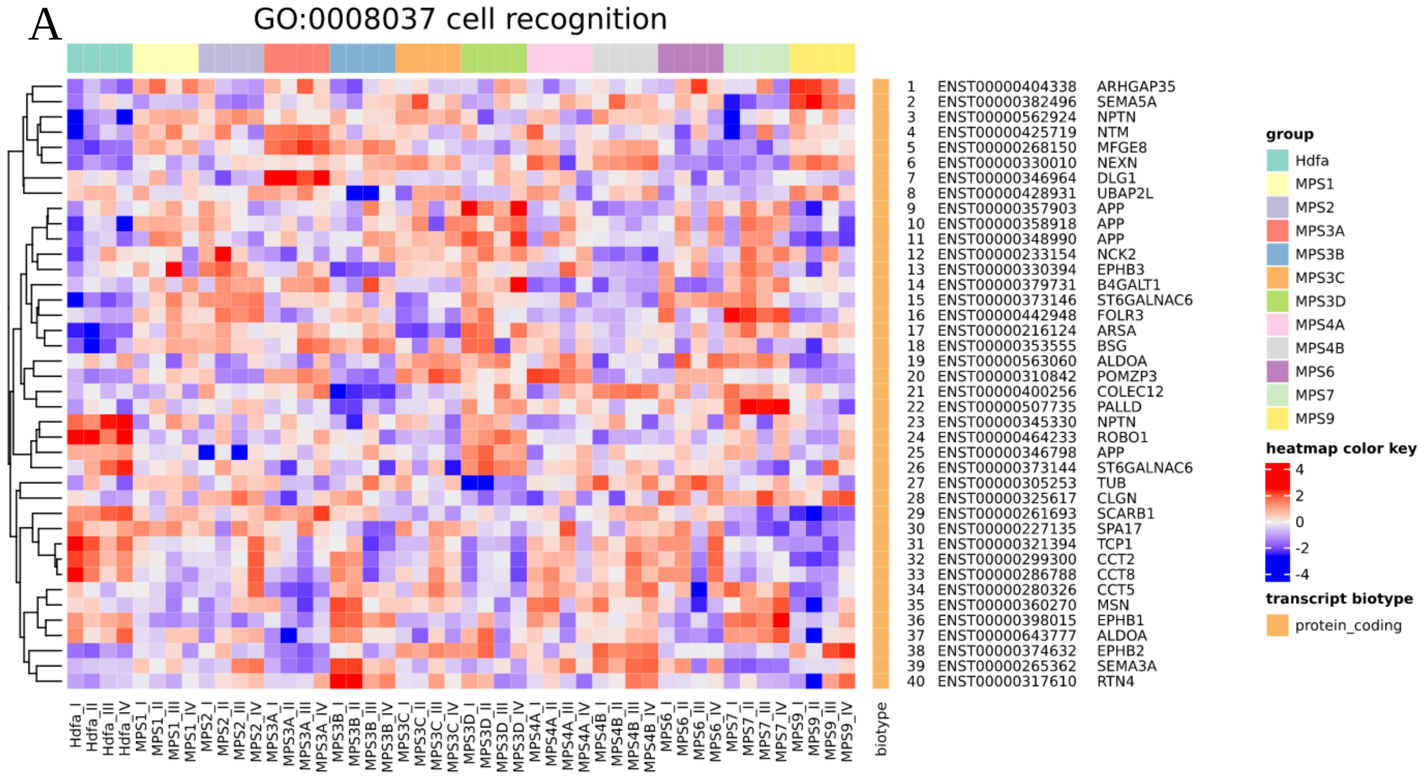

B

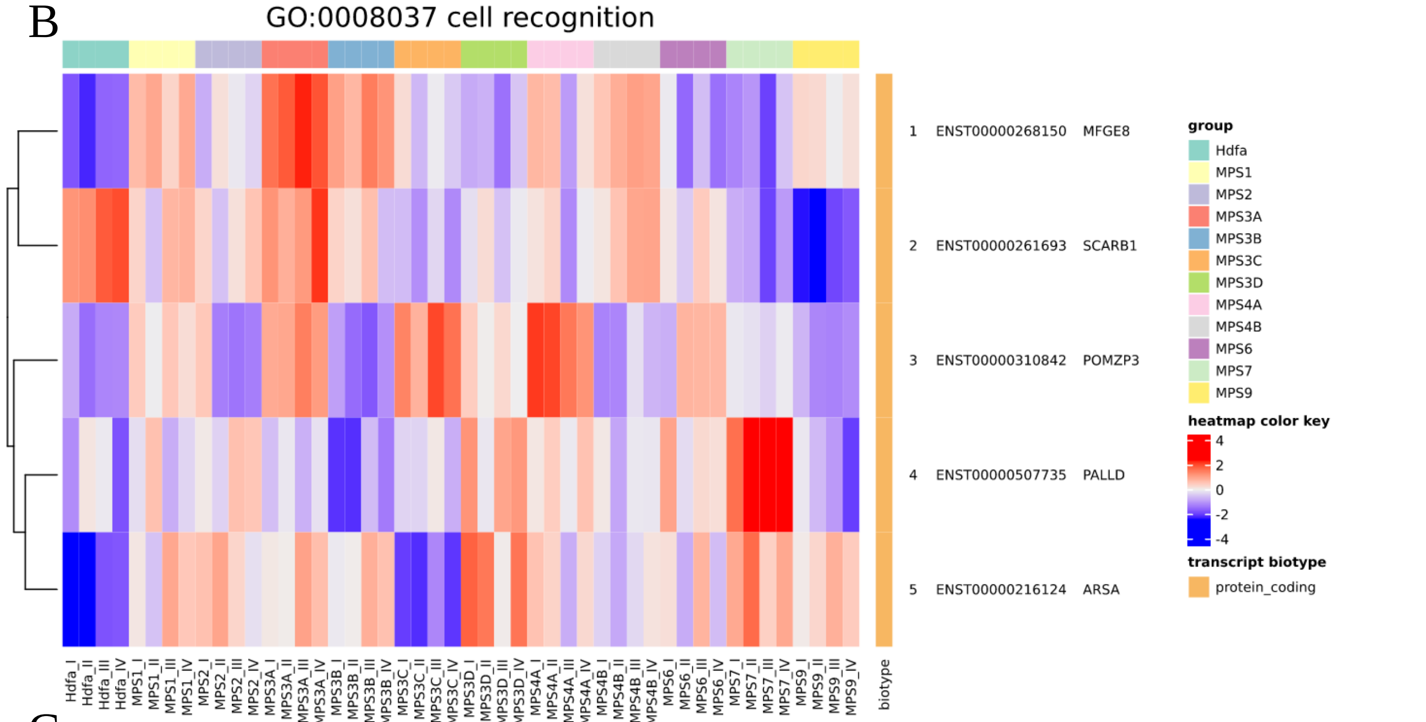

C

Transcripts in **Cell recogniton** process	Significant changes in particular MPS type vs HDFa line										
	I	II	IIIA	IIIB	IIIC	IIID	IVA	IVB	VI	VII	IX
Up-regulated (FDR<0.1)	3	2	5	3	4	2	2	5	0	3	3
Up-regulated (FDR<0.000001)	3	2	3	2	2	2	2	2	0	2	2
Down-regulated (FDR<0.1)	3	1	0	1	3	2	1	1	3	3	3
Down-regulated (FDR<0.000001)	0	0	0	0	1	1	0	0	1	1	1

Fig. S2-16

A

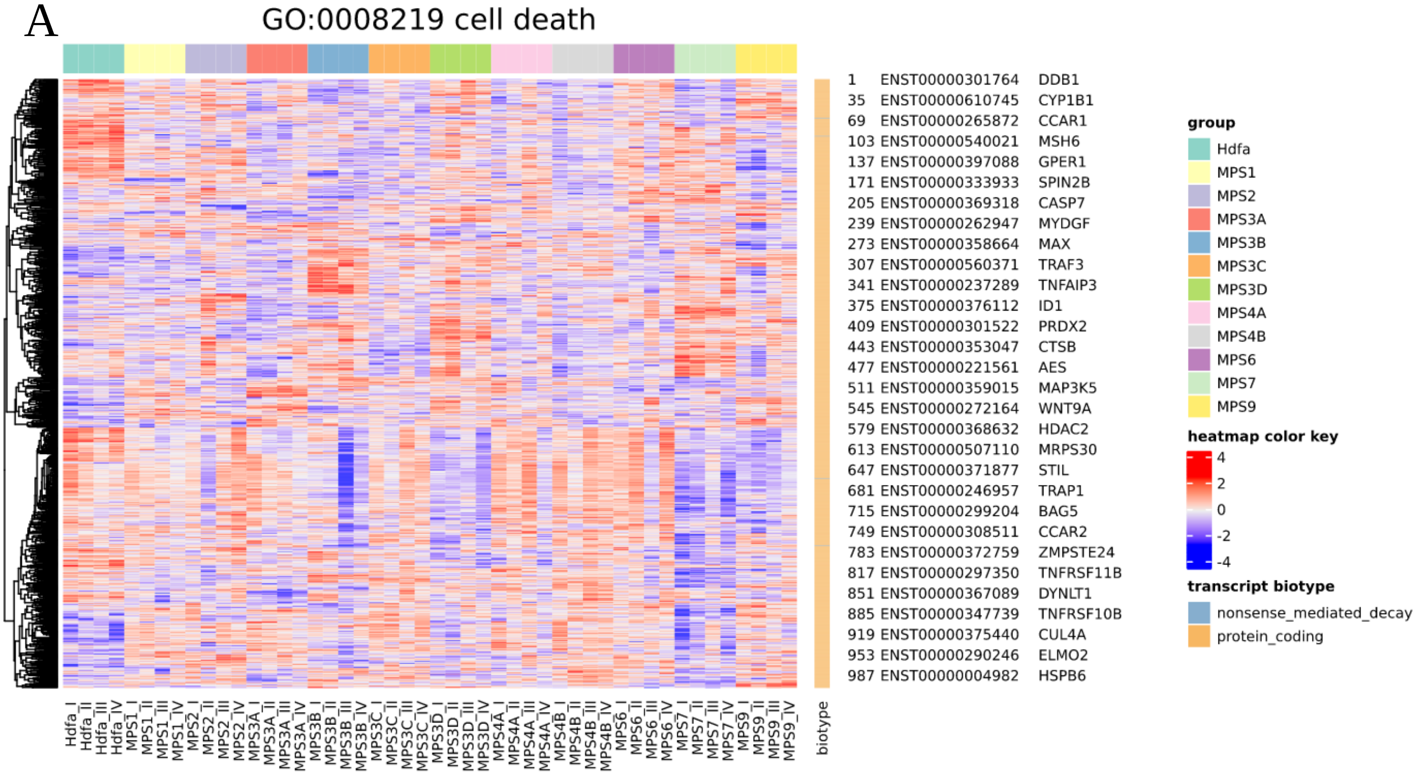

B

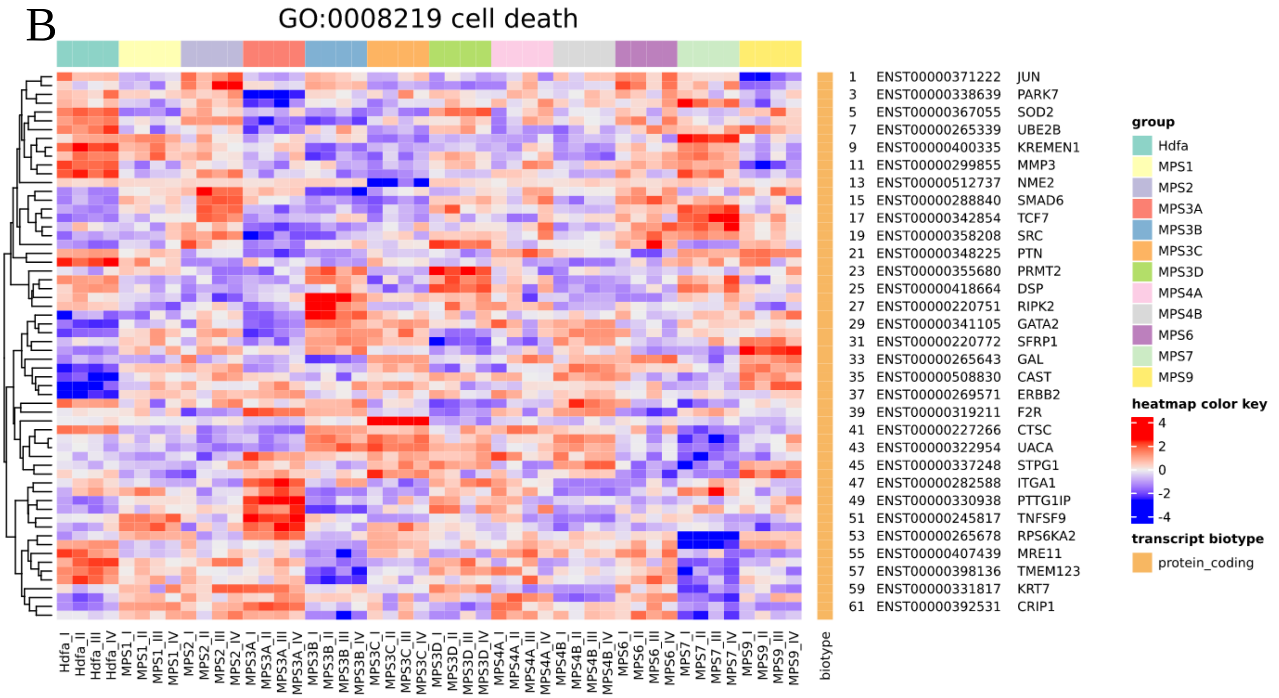

C

Transcripts in **Cell death** process	Significant changes in particular MPS type vs HDFa line										
	I	II	IIIA	IIIB	IIIC	IIID	IVA	IVB	VI	VII	IX
Up-regulated (FDR<0.1)	30	19	35	41	33	34	7	46	20	35	36
Up-regulated (FDR<0.000001)	7	6	12	10	7	6	3	9	6	8	9
Down-regulated (FDR<0.1)	47	15	58	45	43	39	19	63	14	52	47
Down-regulated (FDR<0.000001)	8	4	11	11	11	6	3	15	3	9	5

Fig. S2-17

A

GO:0016043 cellular component organization

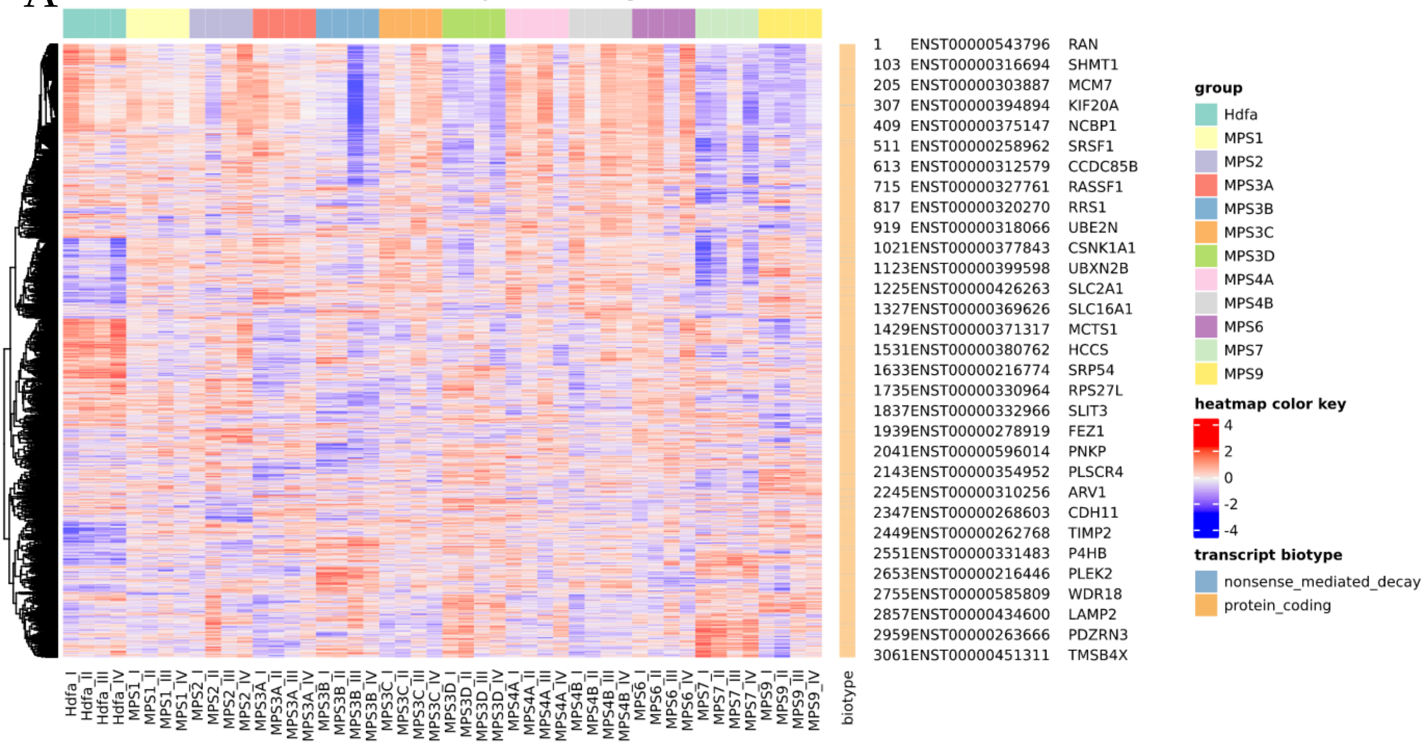

B

GO:0016043 cellular component organization

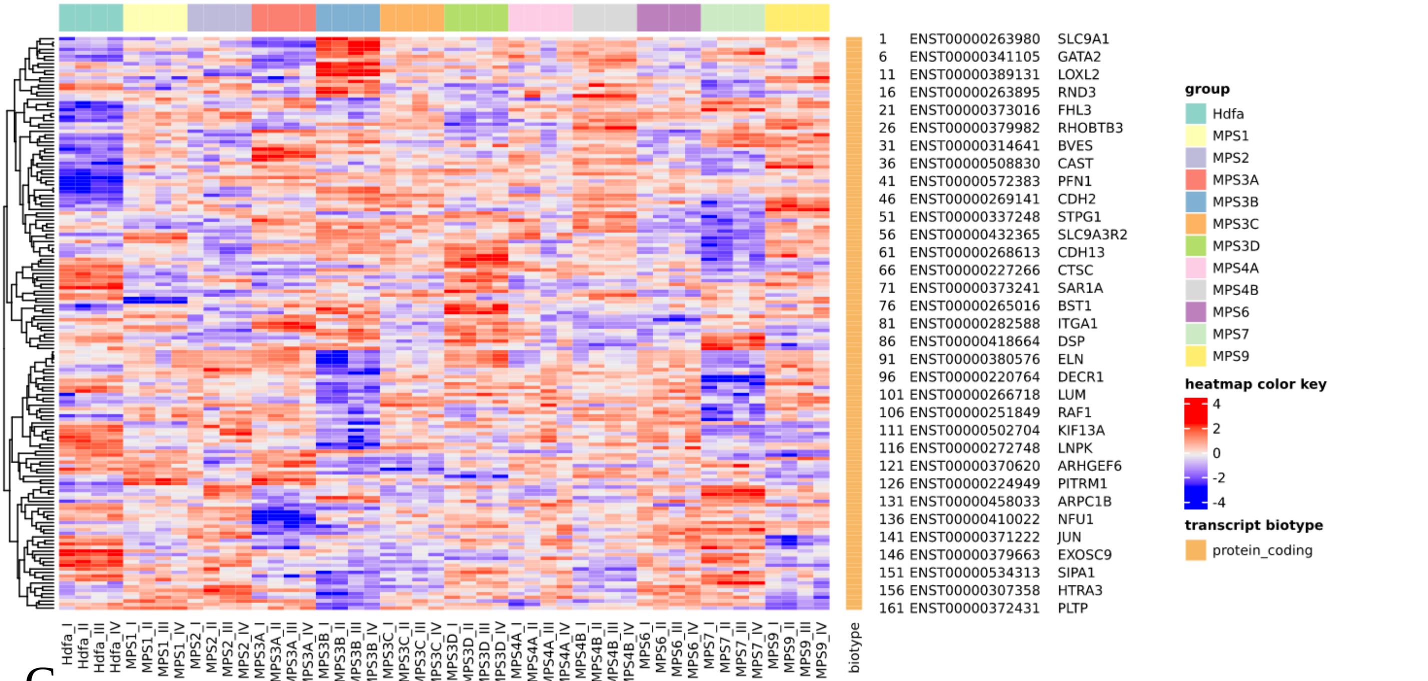

C

Transcripts in **Cellular component organisation** process	Significant changes in particular MPS type vs HDFa line										
	I	II	IIIA	IIIB	IIIC	IIID	IVA	IVB	VI	VII	IX
Up-regulated (FDR<0.1)	101	64	126	134	122	99	50	154	62	121	126
Up-regulated (FDR<0.000001)	28	19	36	35	27	26	13	40	26	29	37
Down-regulated (FDR<0.1)	123	41	161	132	111	120	48	138	40	136	172
Down-regulated (FDR<0.000001)	25	12	27	28	21	15	8	28	7	28	26

Fig. S2-18

A

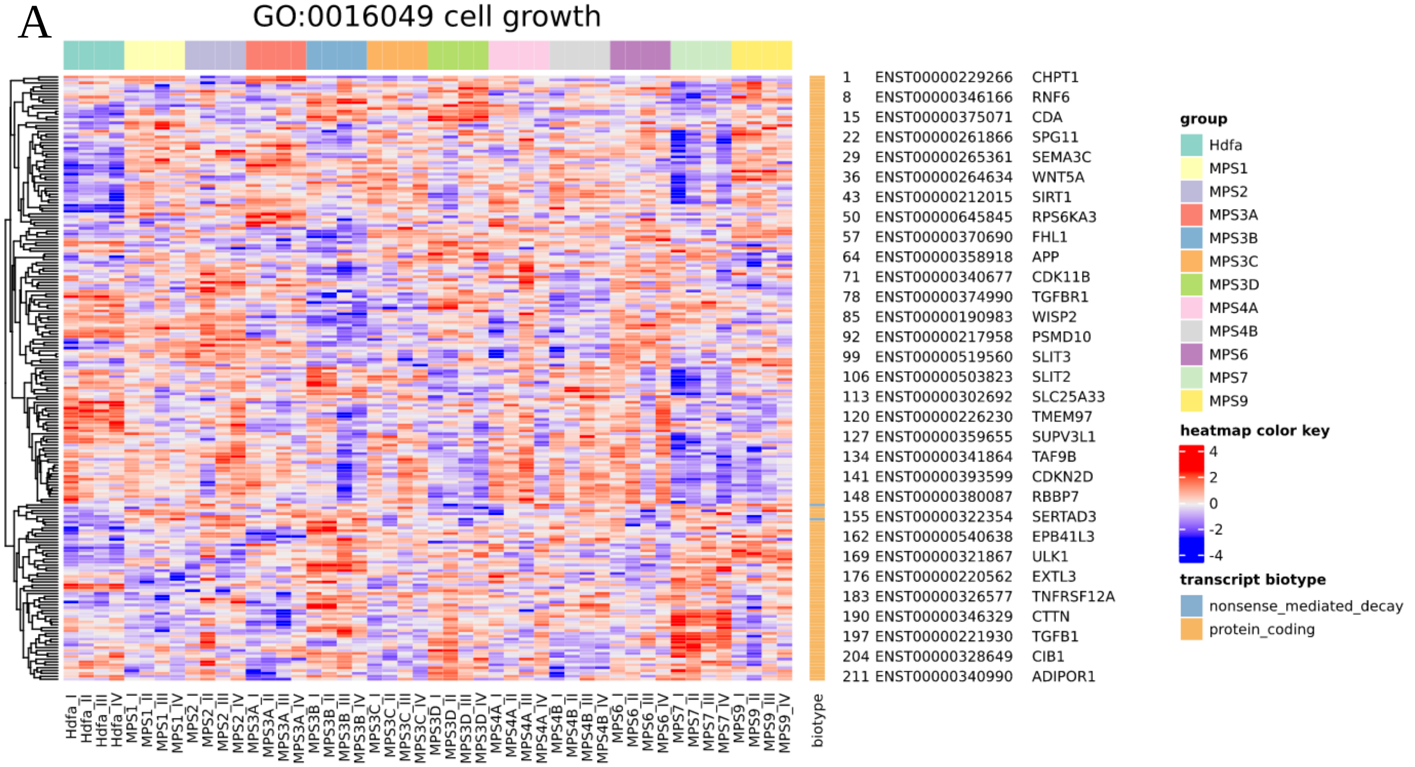

B

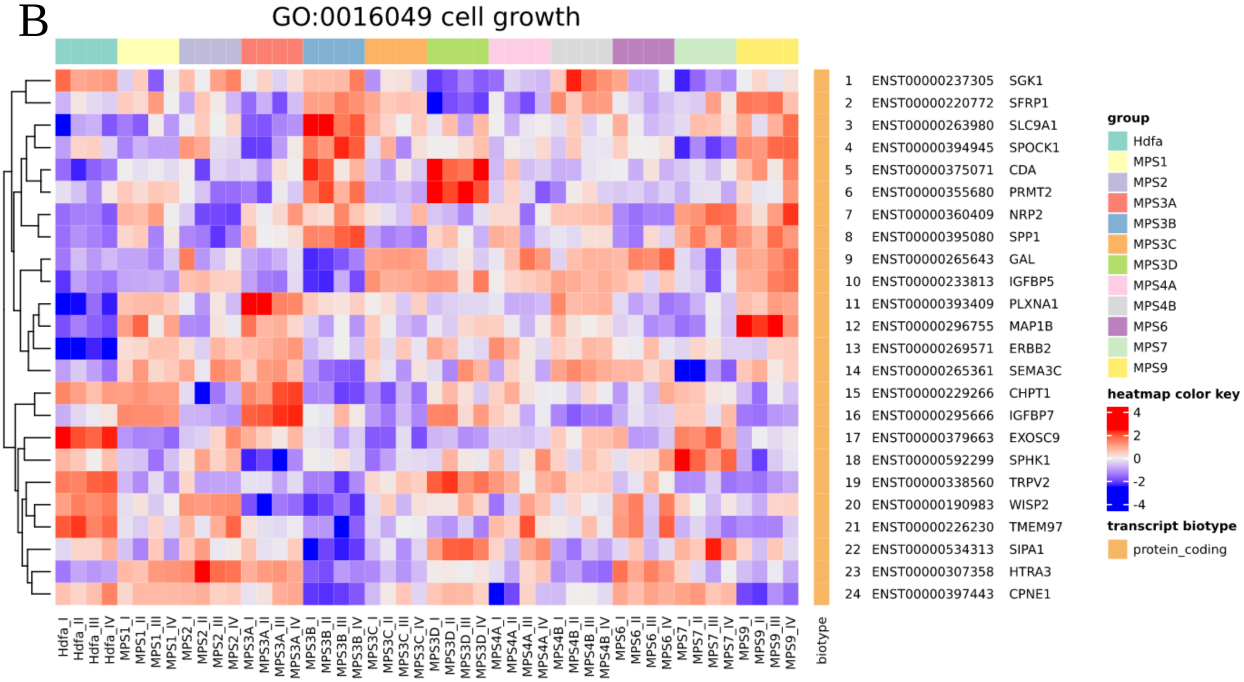

C

Transcripts in **Cell growth** process	Significant changes in particular MPS type vs HDFa line										
	I	II	IIIA	IIIB	IIIC	IIID	IVA	IVB	VI	VII	IX
Up-regulated (FDR<0.1)	7	2	14	15	14	7	4	11	6	10	14
Up-regulated (FDR<0.000001)	3	1	6	4	3	4	1	5	2	2	6
Down-regulated (FDR<0.1)	11	0	9	10	11	9	4	8	2	7	9
Down-regulated (FDR<0.000001)	4	0	4	6	4	4	2	4	0	3	4

Fig. S2-19

A

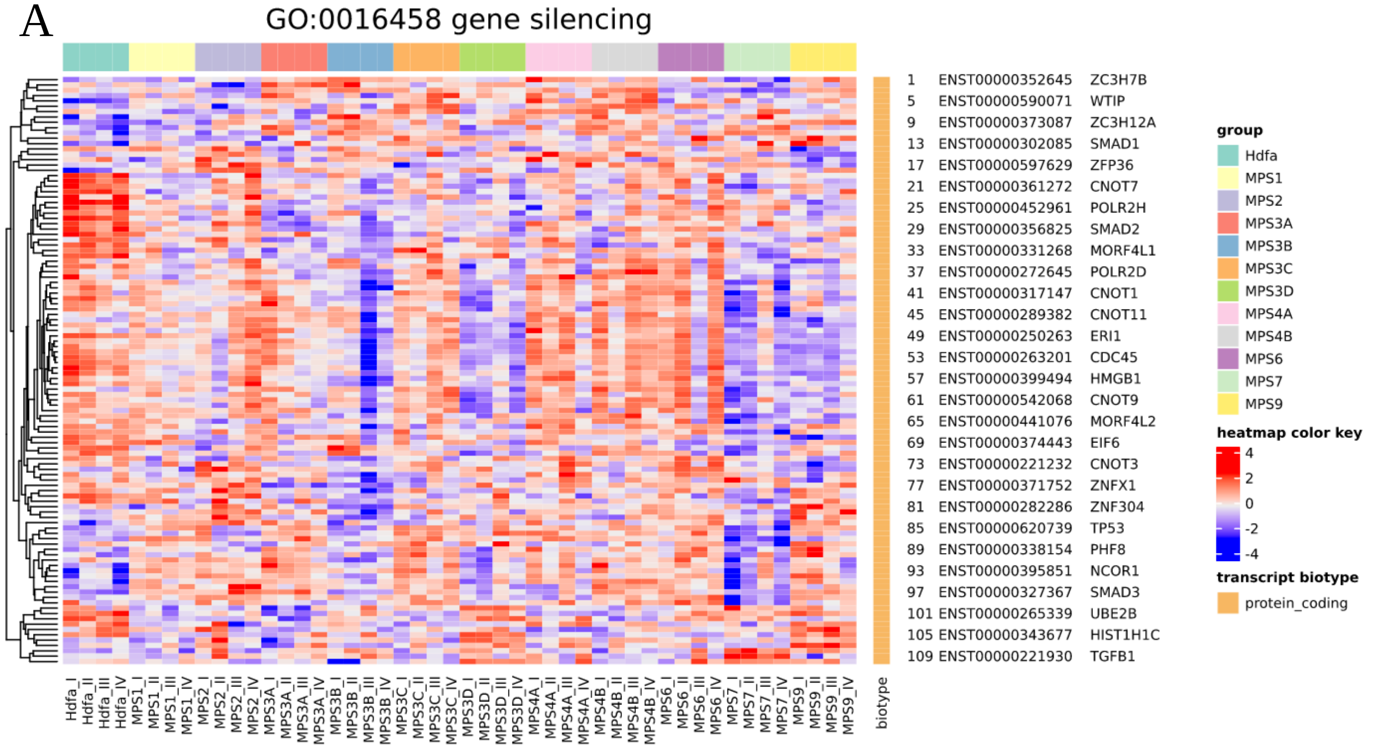

B

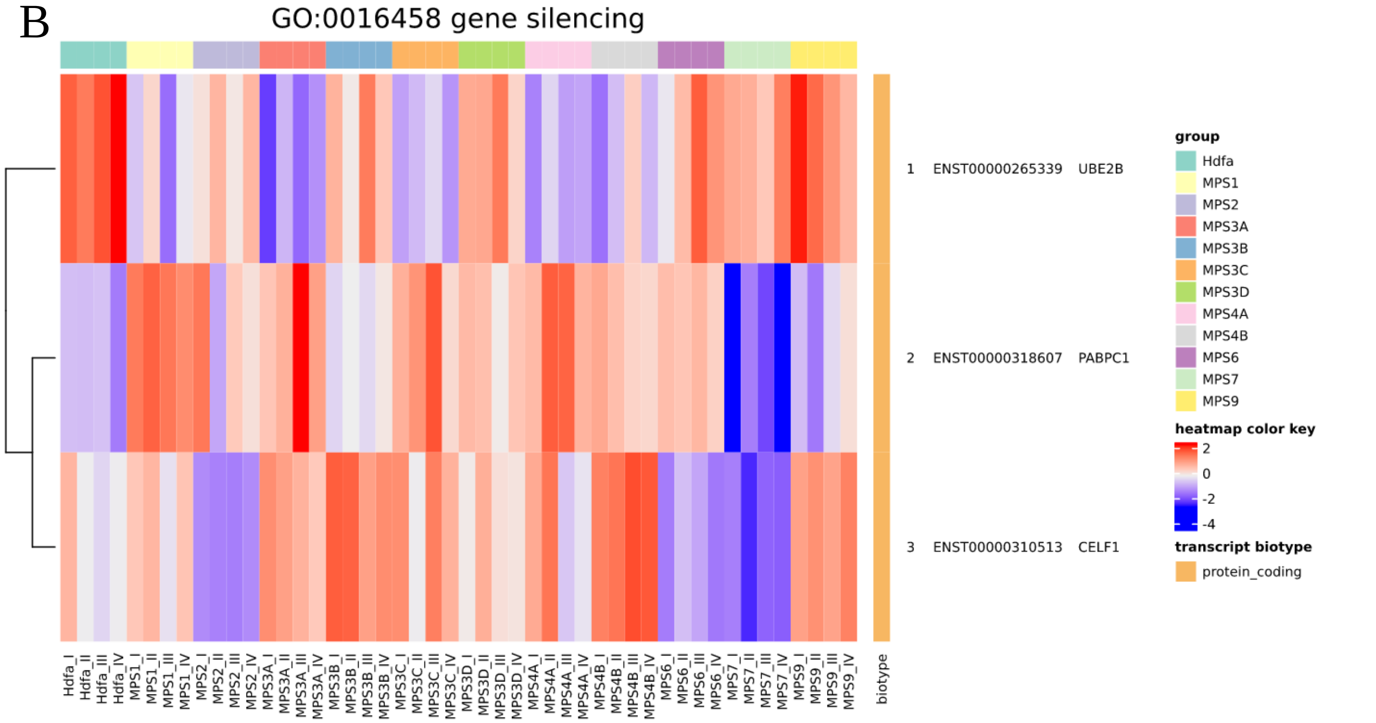

C

Transcripts in **Gene silencing** process	Significant changes in particular MPS type vs HDFa line										
	I	II	IIIA	IIIB	IIIC	IIID	IVA	IVB	VI	VII	IX
Up-regulated (FDR<0.1)	2	1	2	3	4	2	0	4	0	3	4
Up-regulated (FDR<0.000001)	1	0	0	0	0	0	0	0	0	0	0
Down-regulated (FDR<0.1)	6	2	8	7	2	8	3	3	1	4	8
Down-regulated (FDR<0.000001)	0	0	1	0	1	0	1	0	0	1	0

Fig. S2-20

A

GO:0019725 cellular homeostasis

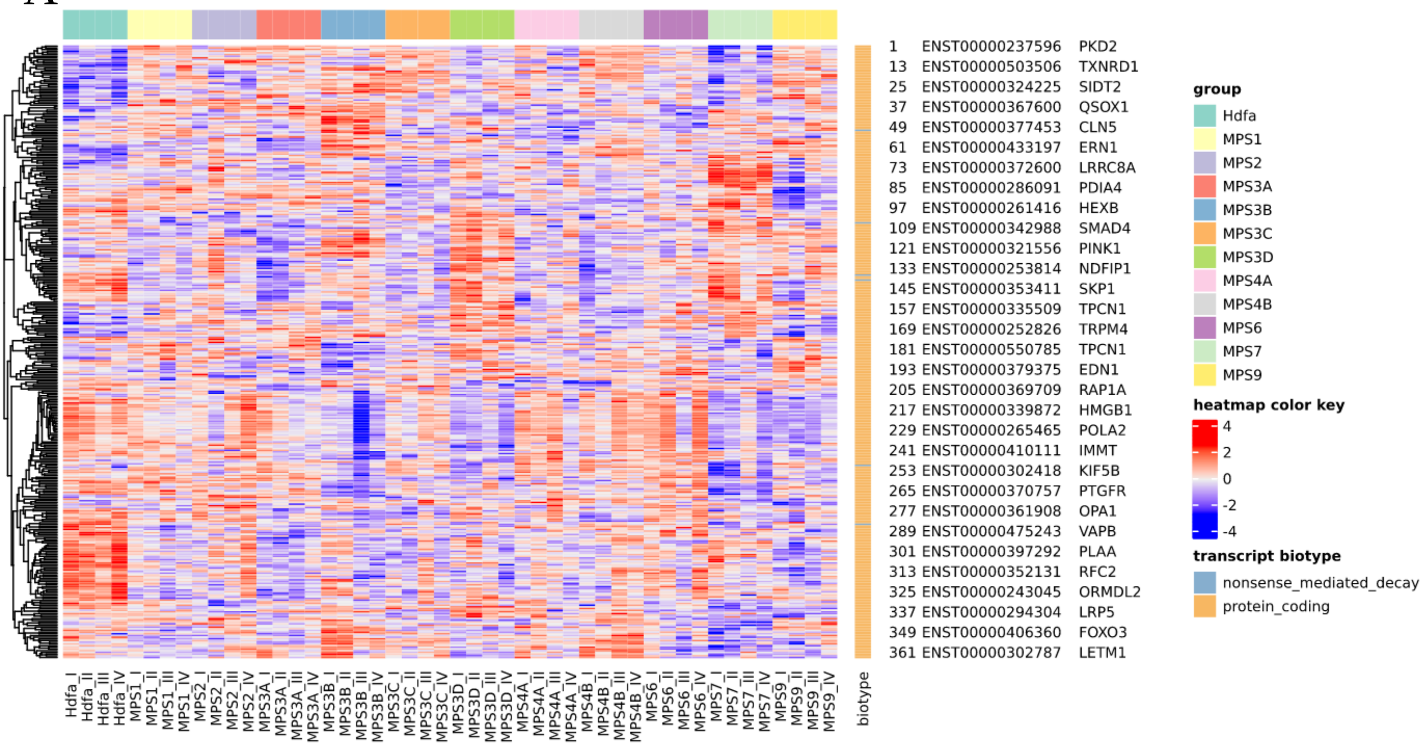

B

GO:0019725 cellular homeostasis

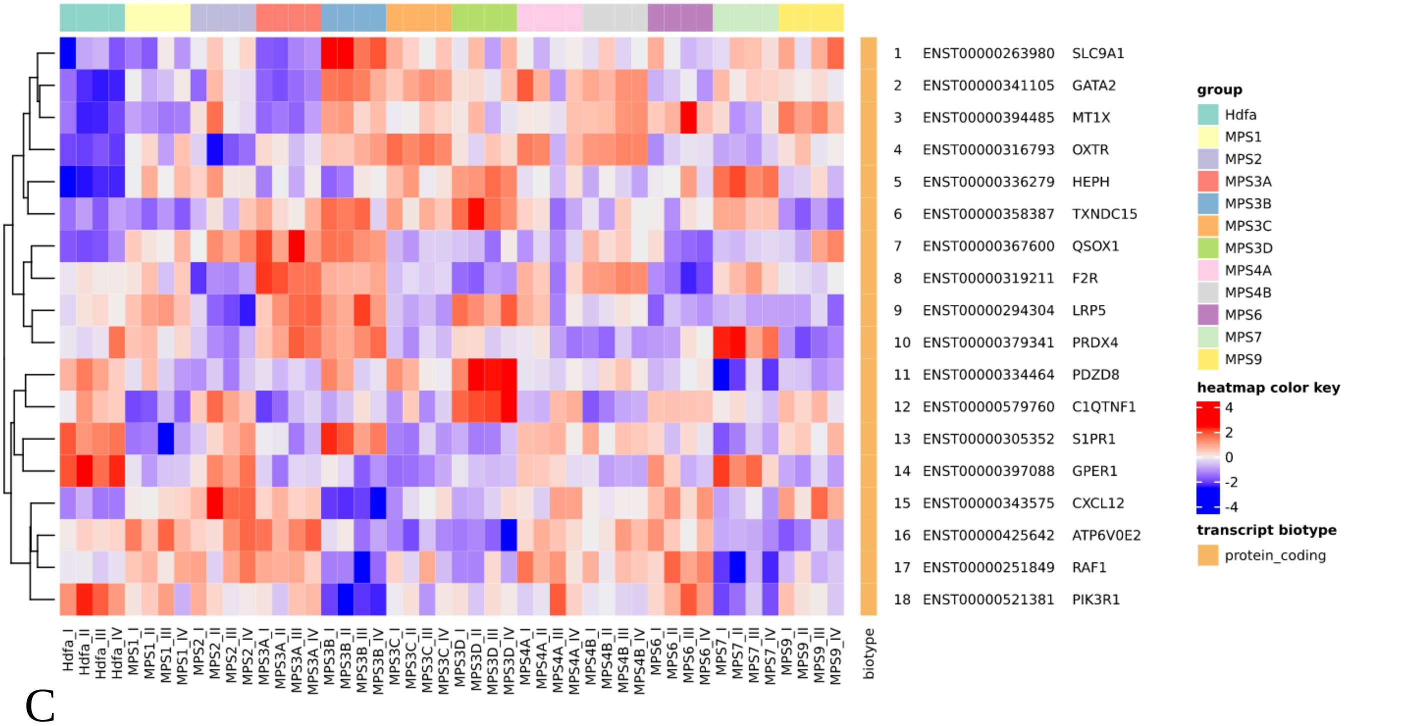

C

Transcripts in **Cellular homeostasis** process	Significant changes in particular MPS type vs HDFa line										
	I	II	IIIA	IIIB	IIIC	IIID	IVA	IVB	VI	VII	IX
Up-regulated (FDR<0.1)	9	3	13	17	11	7	3	18	3	14	10
Up-regulated (FDR<0.000001)	4	1	6	6	5	4	2	6	1	4	4
Down-regulated (FDR<0.1)	23	4	19	13	12	22	8	15	2	19	21
Down-regulated (FDR<0.000001)	2	1	1	1	2	2	1	1	1	3	2

Fig. S2-21

A

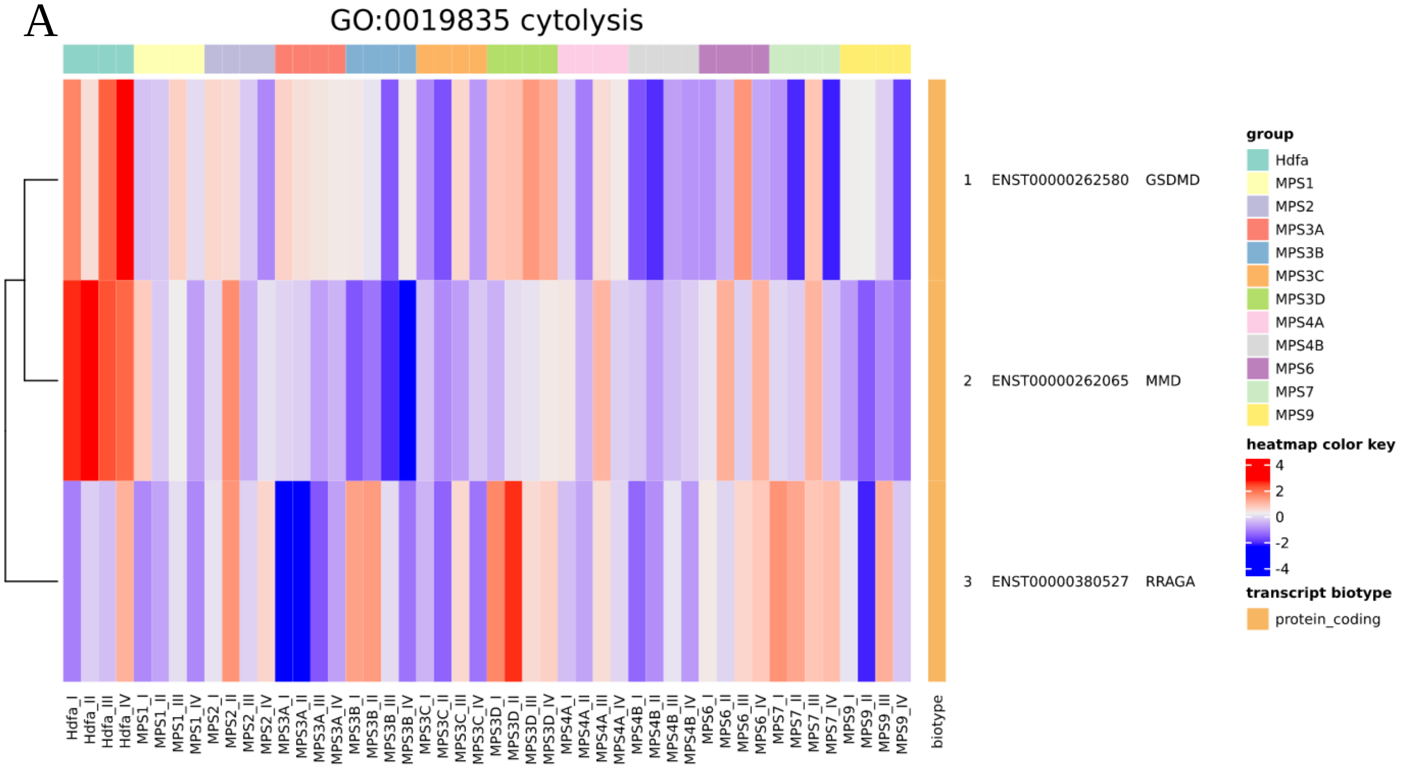

B

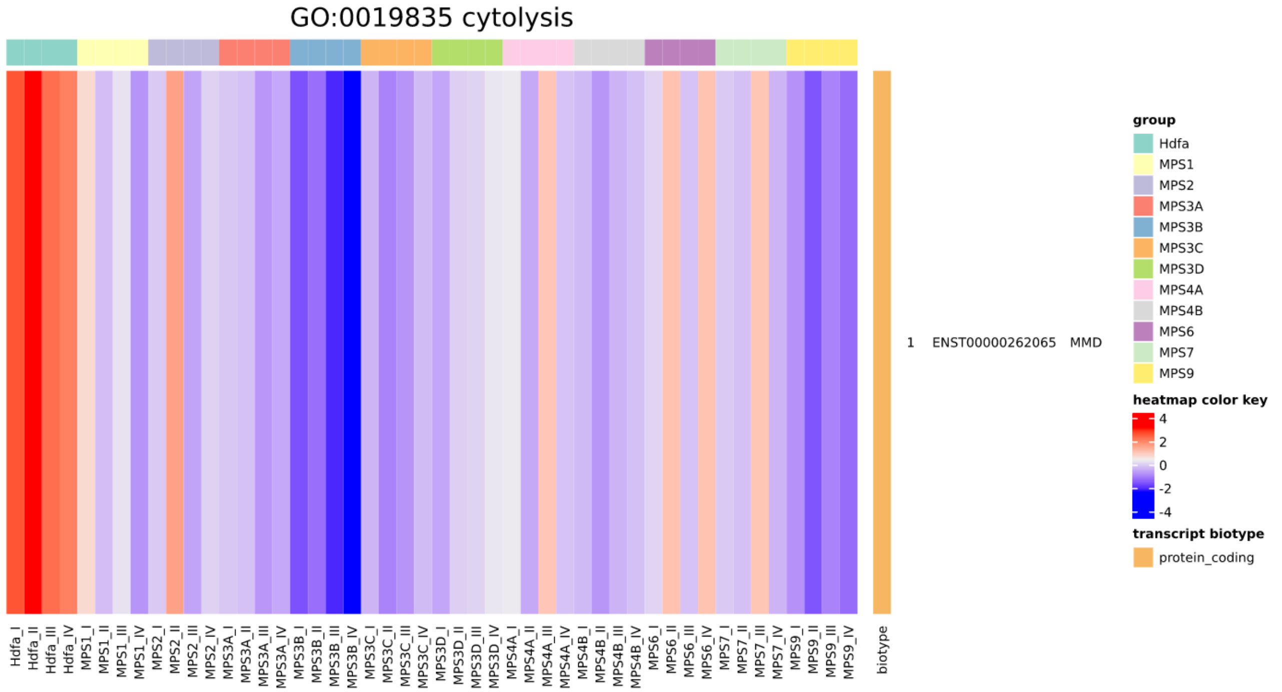

C

Transcripts in **Cytolysis** process	Significant changes in particular MPS type vs HDFa line										
	I	II	IIIA	IIIB	IIIC	IIID	IVA	IVB	VI	VII	IX
Up-regulated (FDR<0.1)	0	0	0	0	0	0	0	0	0	0	0
Up-regulated (FDR<0.000001)	0	0	0	0	0	0	0	0	0	0	0
Down-regulated (FDR<0.1)	1	0	1	1	1	1	0	1	0	1	1
Down-regulated (FDR<0.000001)	1	0	1	1	1	1	0	1	0	1	1

Fig. S2-22

A

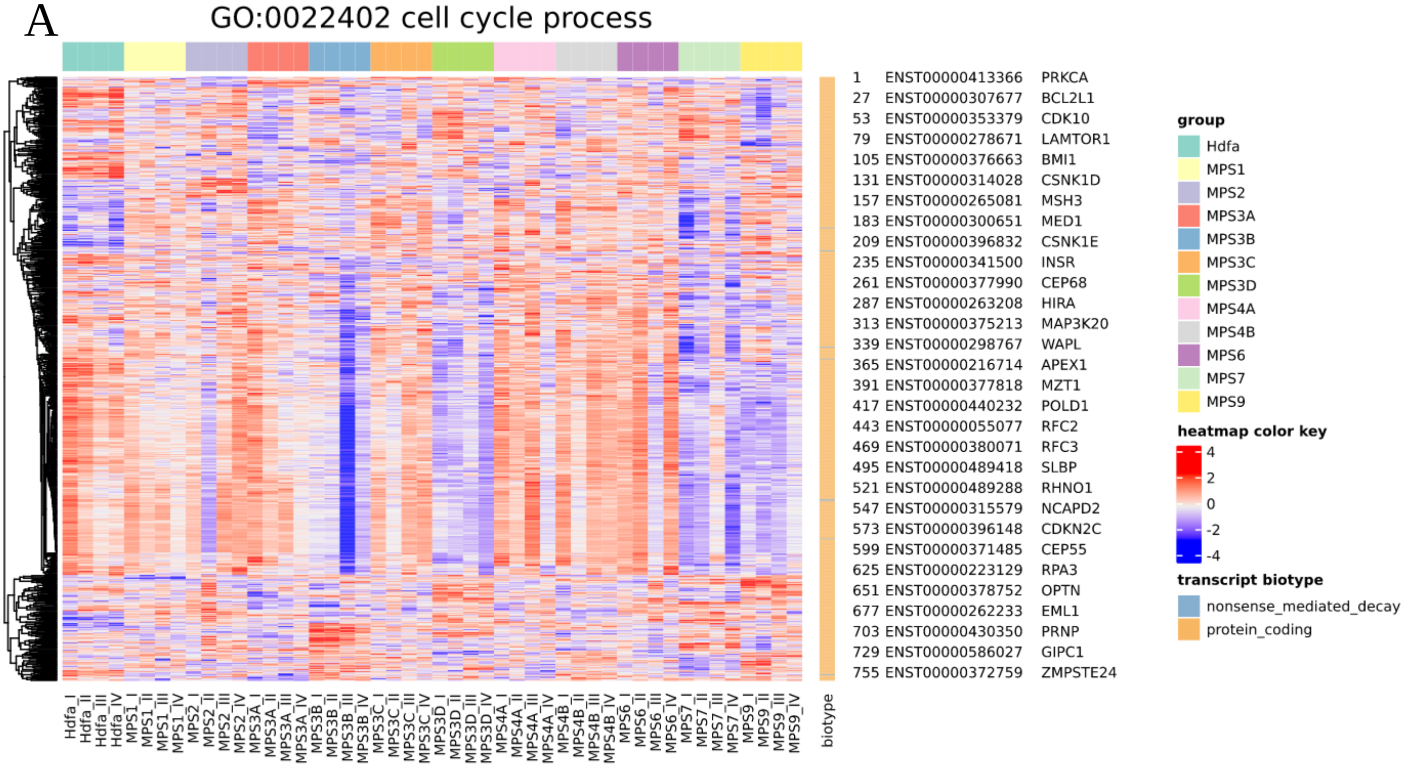

B

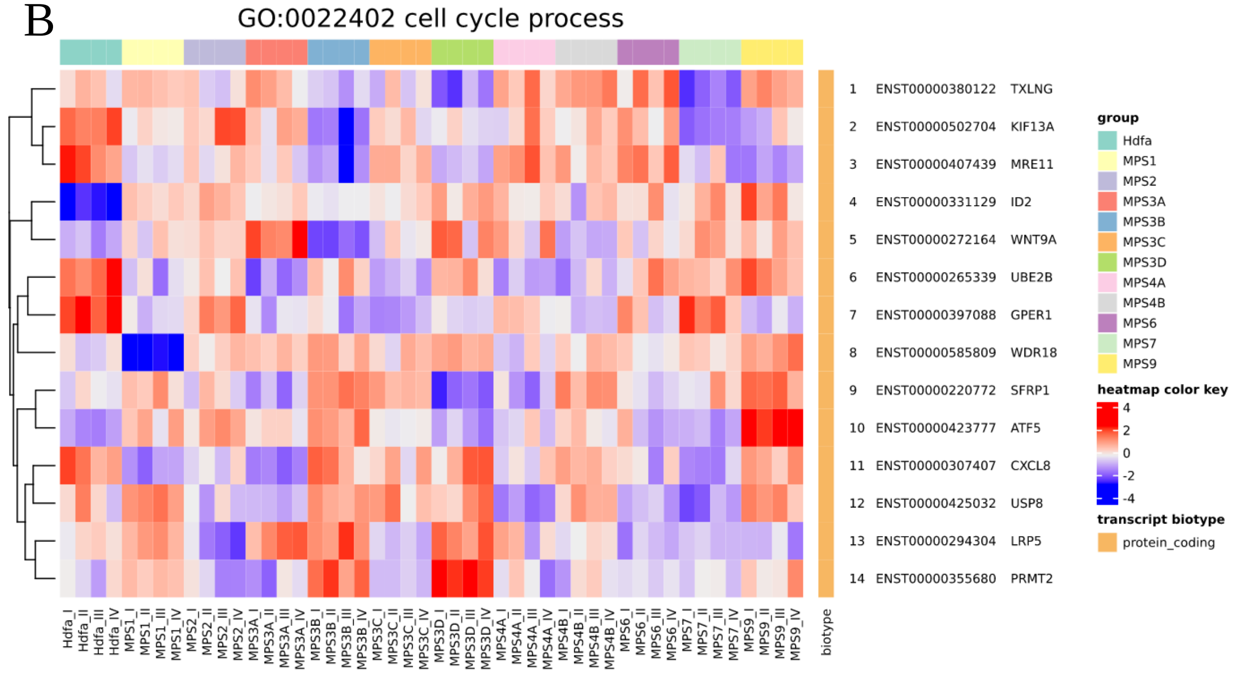

C

Transcripts in **Cell Cycle process**	Significant changes in particular MPS type vs HDFa line										
	I	II	IIIA	IIIB	IIIC	IIID	IVA	IVB	VI	VII	IX
Up-regulated (FDR<0.1)	9	7	17	12	21	13	6	21	5	17	15
Up-regulated (FDR<0.000001)	0	1	2	2	0	2	1	0	1	1	3
Down-regulated (FDR<0.1)	33	8	29	21	24	65	8	23	4	36	69
Down-regulated (FDR<0.000001)	4	0	3	2	2	3	2	1	0	3	2

Fig. S2-23

A

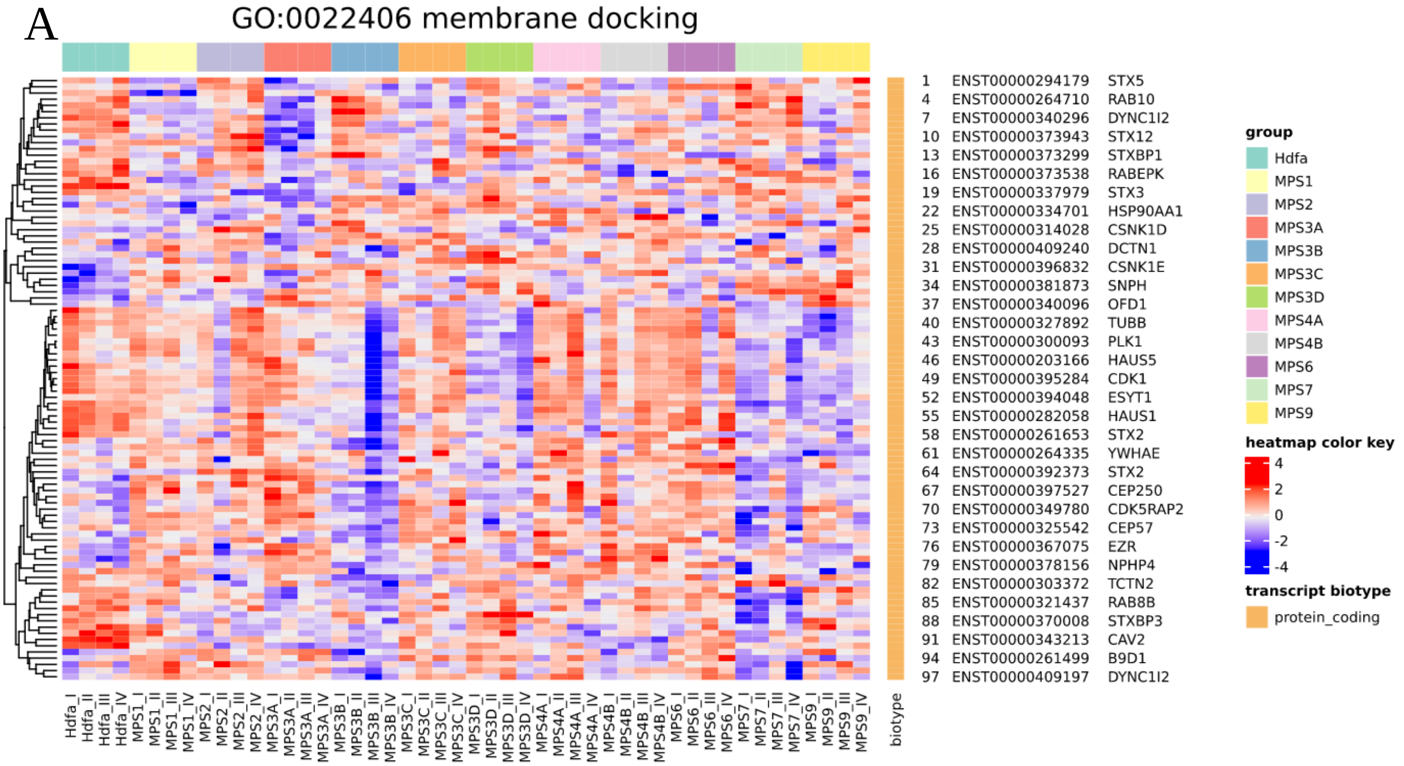

B

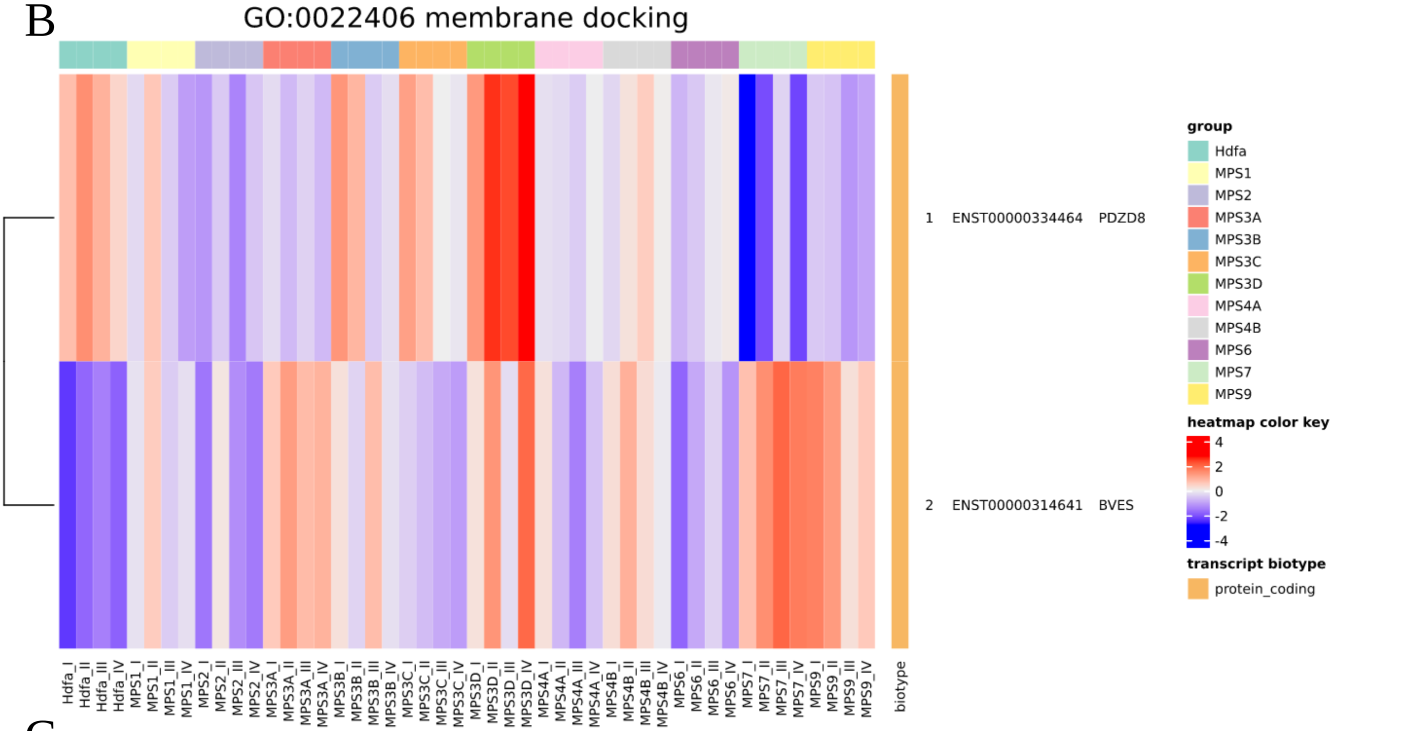

C

Transcripts in **Membrane docking** process	Significant changes in particular MPS type vs HDFa line										
	I	II	IIIA	IIIB	IIIC	IIID	IVA	IVB	VI	VII	IX
Up-regulated (FDR<0.1)	1	0	1	1	0	0	0	2	1	1	1
Up-regulated (FDR<0.000001)	1	0	1	1	0	0	0	1	0	1	1
Down-regulated (FDR<0.1)	6	1	5	1	3	1	2	4	2	3	5
Down-regulated (FDR<0.000001)	0	1	0	0	0	0	0	0	0	0	1

Fig. S2-24

A 022412 cellular process involved in reproduction in multicellular organism

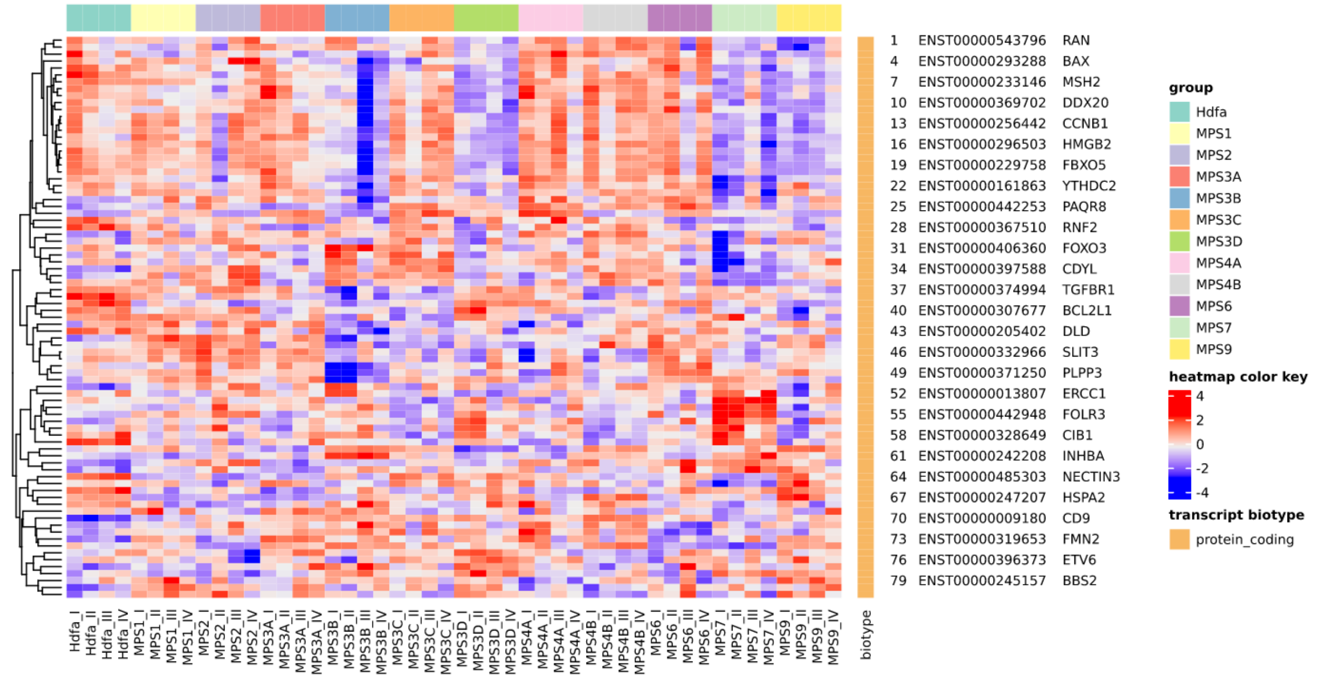

B 022412 cellular process involved in reproduction in multicellular organism

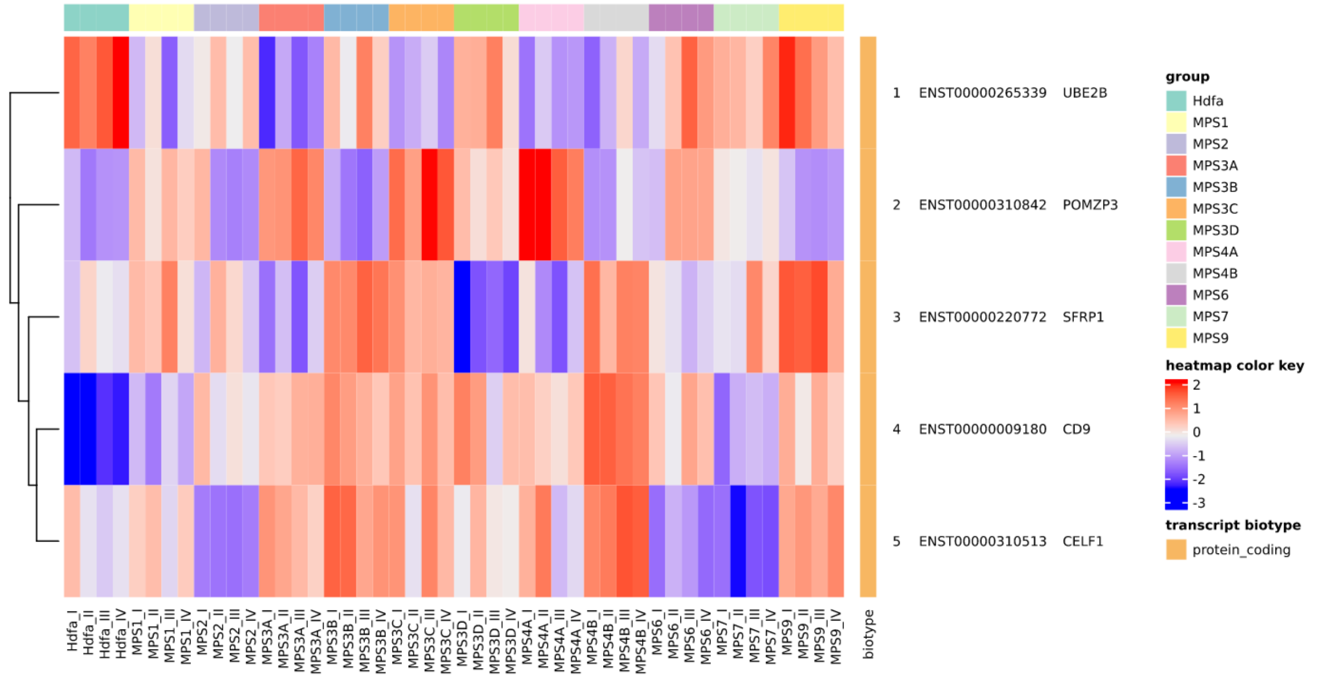

C

Transcripts **involved in reproduction in multicellular organism**	Significant changes in particular MPS type vs HDFa line										
	I	II	IIIA	IIIB	IIIC	IIID	IVA	IVB	VI	VII	IX
Up-regulated (FDR<0.1)	3	4	3	3	4	3	2	3	2	5	1
Up-regulated (FDR<0.000001)	1	1	2	2	2	2	1	1	1	0	1
Down-regulated (FDR<0.1)	3	0	4	4	3	3	1	3	1	4	4
Down-regulated (FDR<0.000001)	0	0	1	0	1	1	1	0	0	1	0

Fig. S2-25

A GO:0030029 actin filament-based process

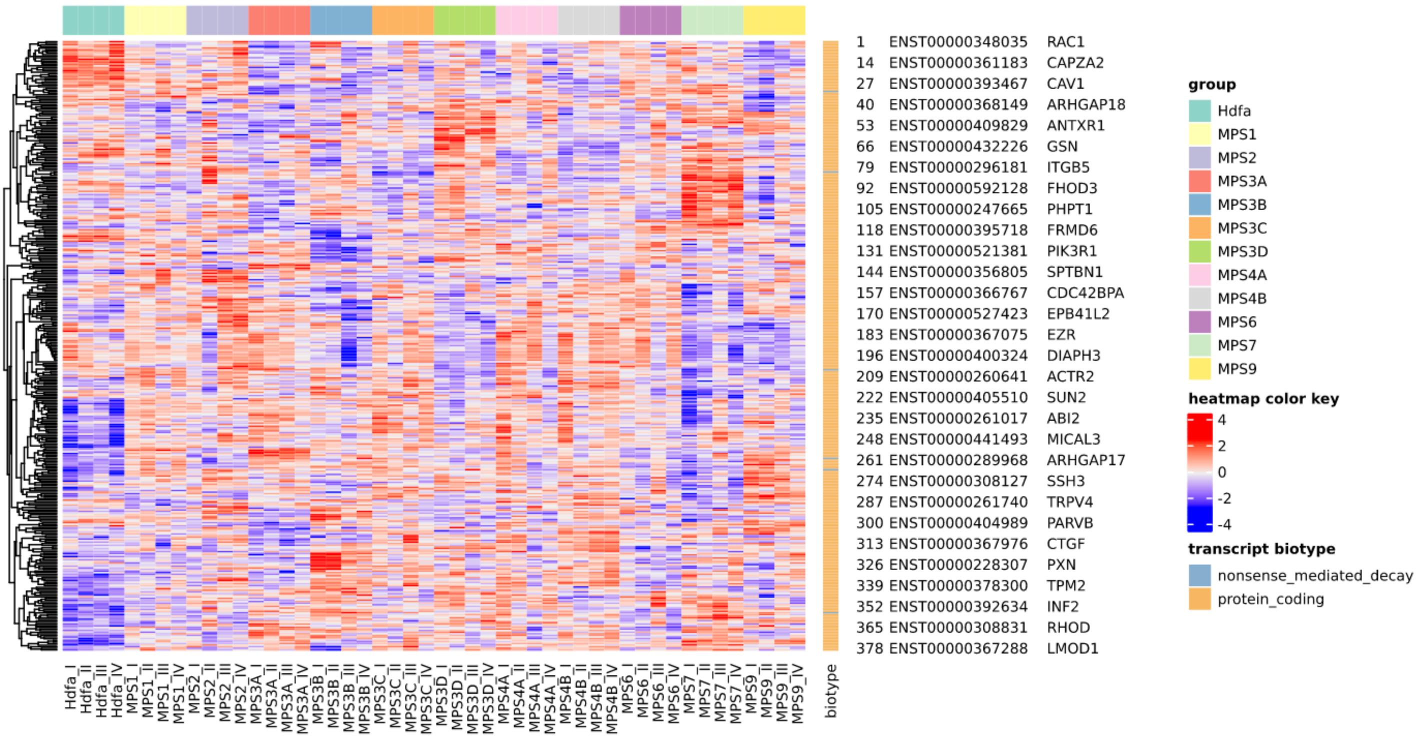

B GO:0030029 actin filament-based process

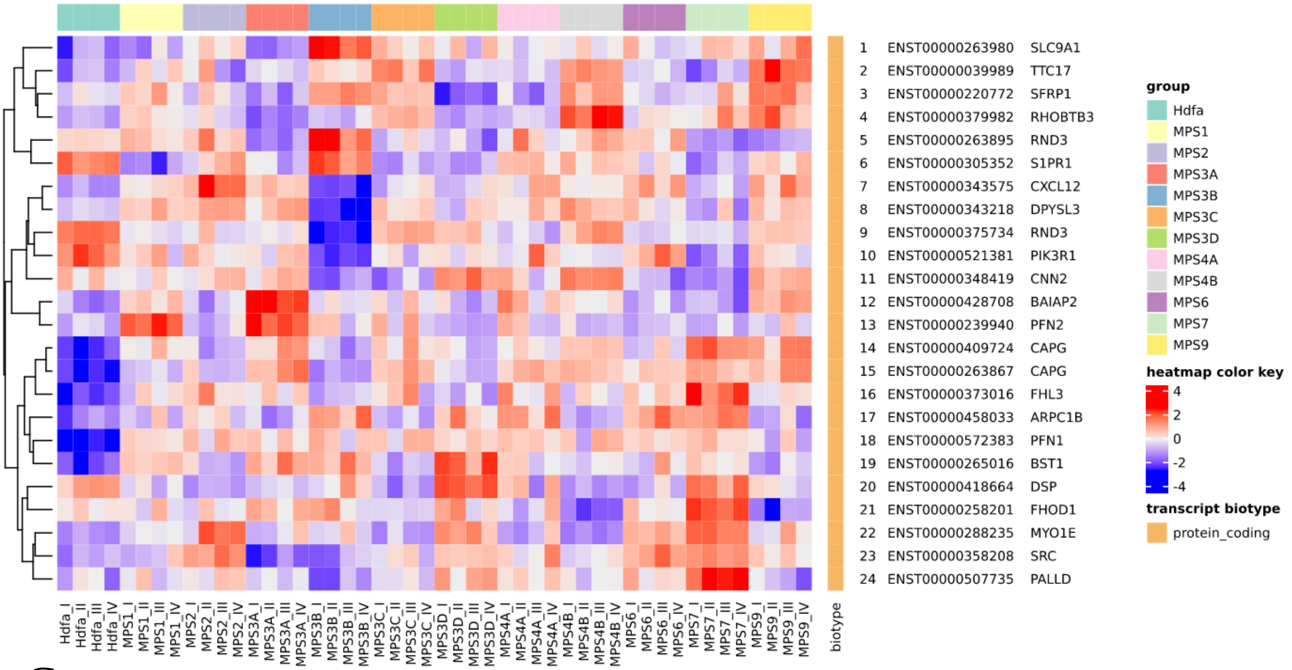

C

Transcripts in **Actin filament - based** process	Significant changes in particular MPS type vs HDFa line										
	I	II	IIIA	IIIB	IIIC	IIID	IVA	IVB	VI	VII	IX
Up-regulated (FDR<0.1)	23	13	27	29	21	18	17	30	12	34	18
Up-regulated (FDR<0.000001)	8	4	8	7	4	4	5	5	7	10	5
Down-regulated (FDR<0.1)	7	16	13	13	13	3	5	18	4	8	12
Down-regulated (FDR<0.000001)	2	1	1	2	2	2	1	2	2	3	1

Fig. S2-26

A

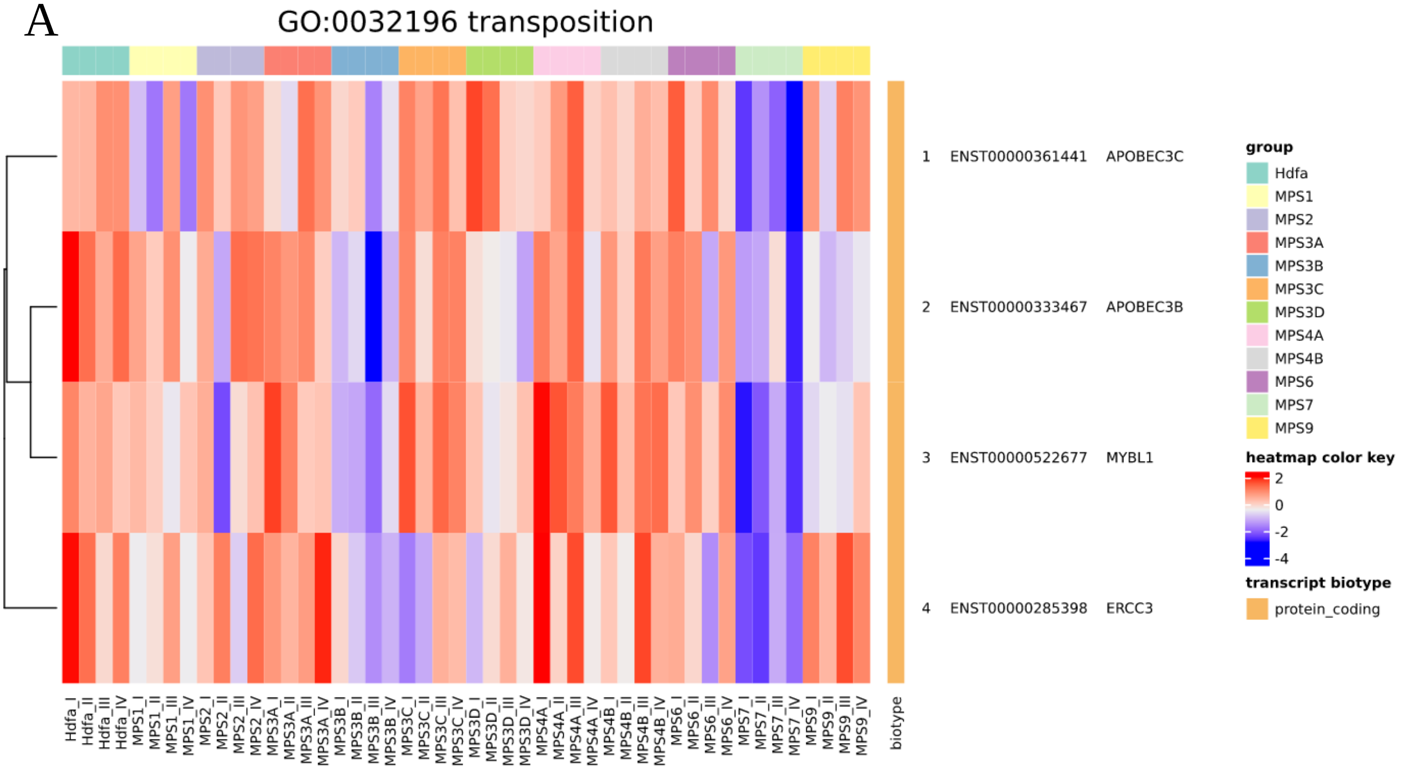

B

Transcripts in **Transposition** process	Significant changes in particular MPS type vs HDFa line										
	I	II	IIIA	IIIB	IIIC	IIID	IVA	IVB	VI	VII	IX
Up-regulated (FDR<0.1)	0	0	0	0	0	0	0	0	0	0	0
Up-regulated (FDR<0.000001)	0	0	0	0	0	0	0	0	0	0	0
Down-regulated (FDR<0.1)	0	0	0	0	0	0	0	0	0	0	0
Down-regulated (FDR<0.000001)	0	0	0	0	0	0	0	0	0	0	0

Fig. S2-27

A

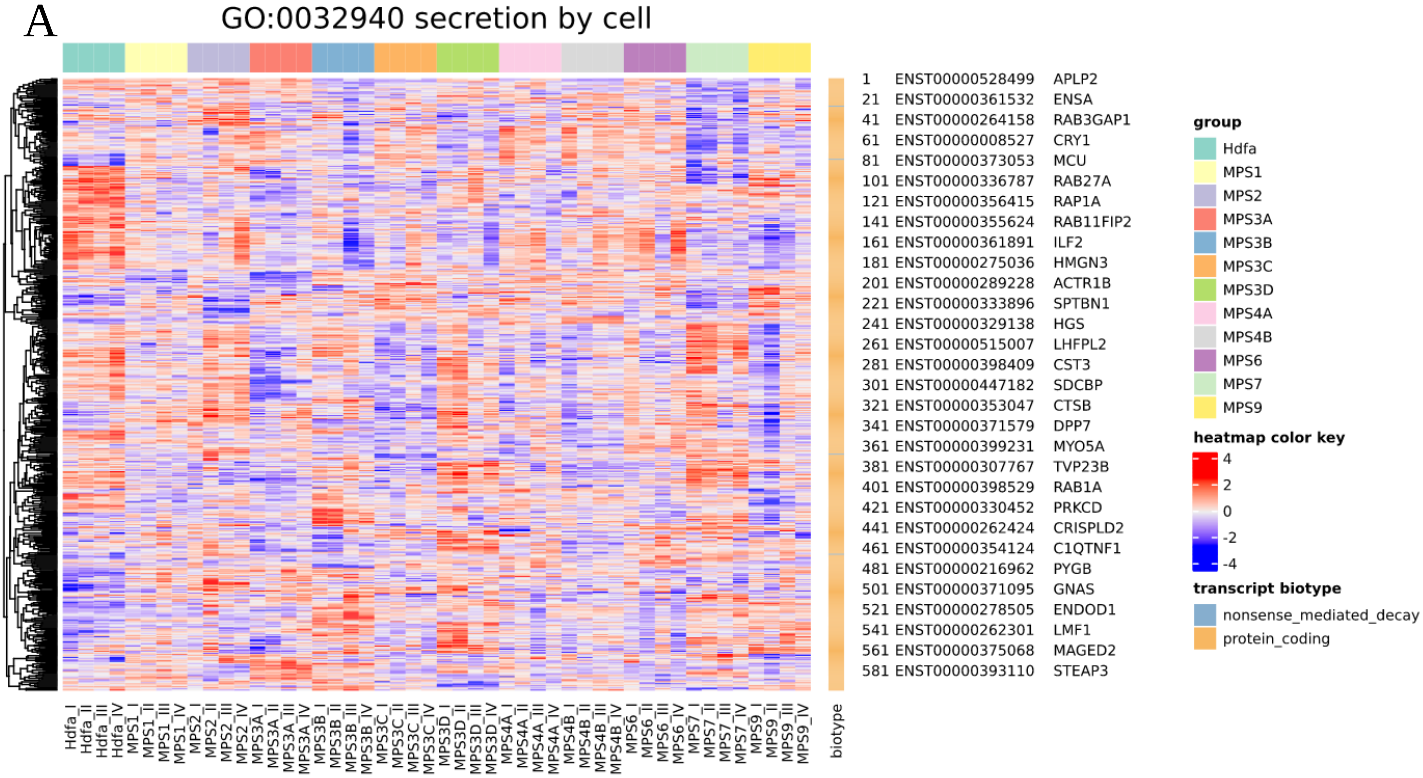

B

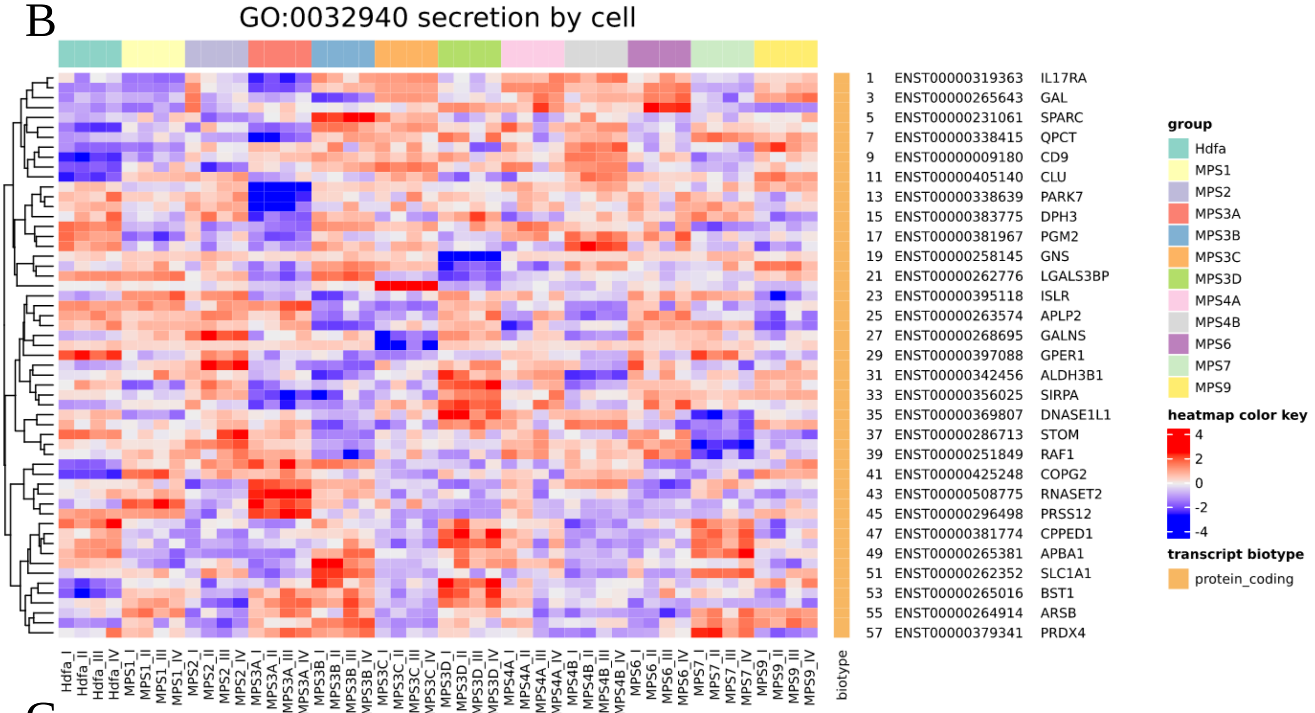

C

Transcripts in **Secretion by cell** process	Significant changes in particular MPS type vs HDFa line										
	I	II	IIIA	IIIB	IIIC	IIID	IVA	IVB	VI	VII	IX
Up-regulated (FDR<0.1)	25	20	24	36	20	18	10	30	9	27	23
Up-regulated (FDR<0.000001)	8	5	12	15	9	9	2	12	3	9	12
Down-regulated (FDR<0.1)	31	11	53	26	39	28	14	46	9	28	35
Down-regulated (FDR<0.000001)	6	2	9	5	7	7	3	13	1	5	6

Fig. S2-28

A

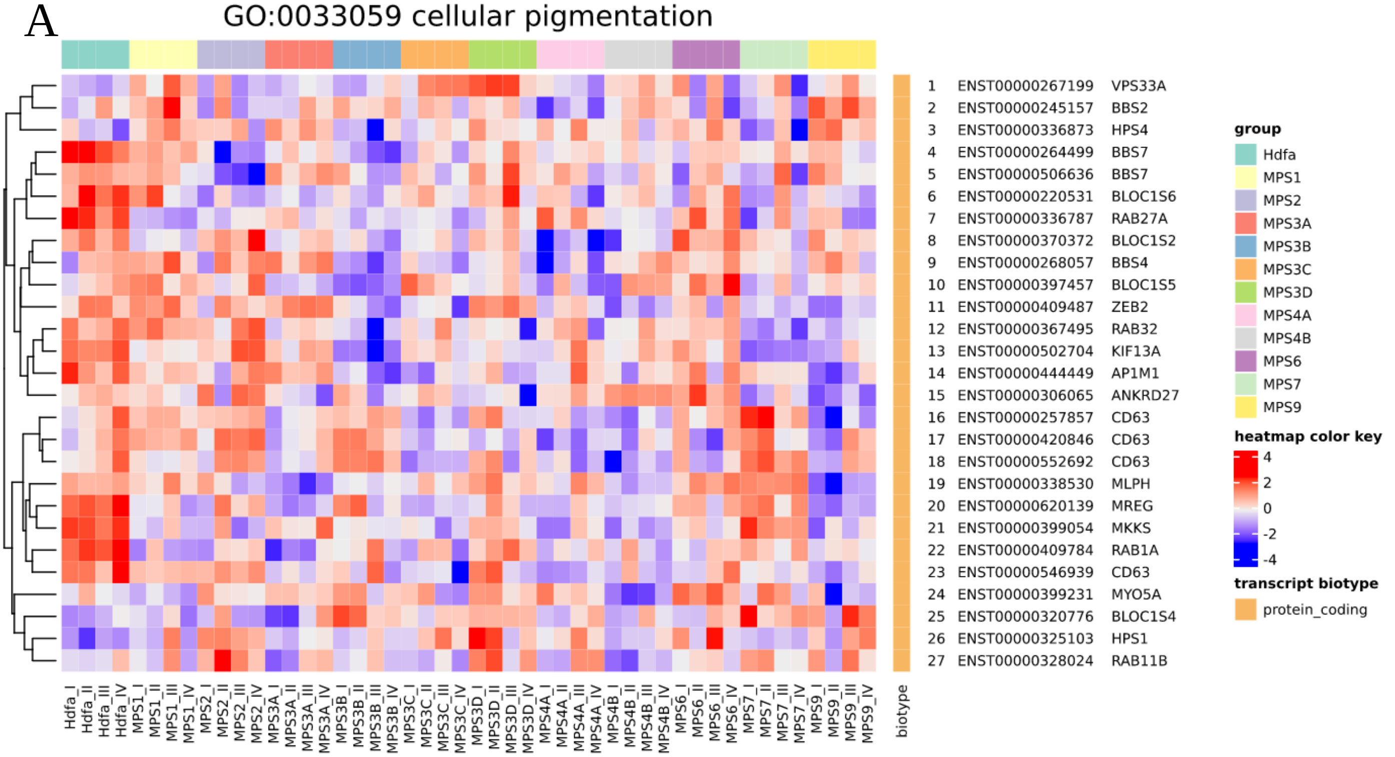

B

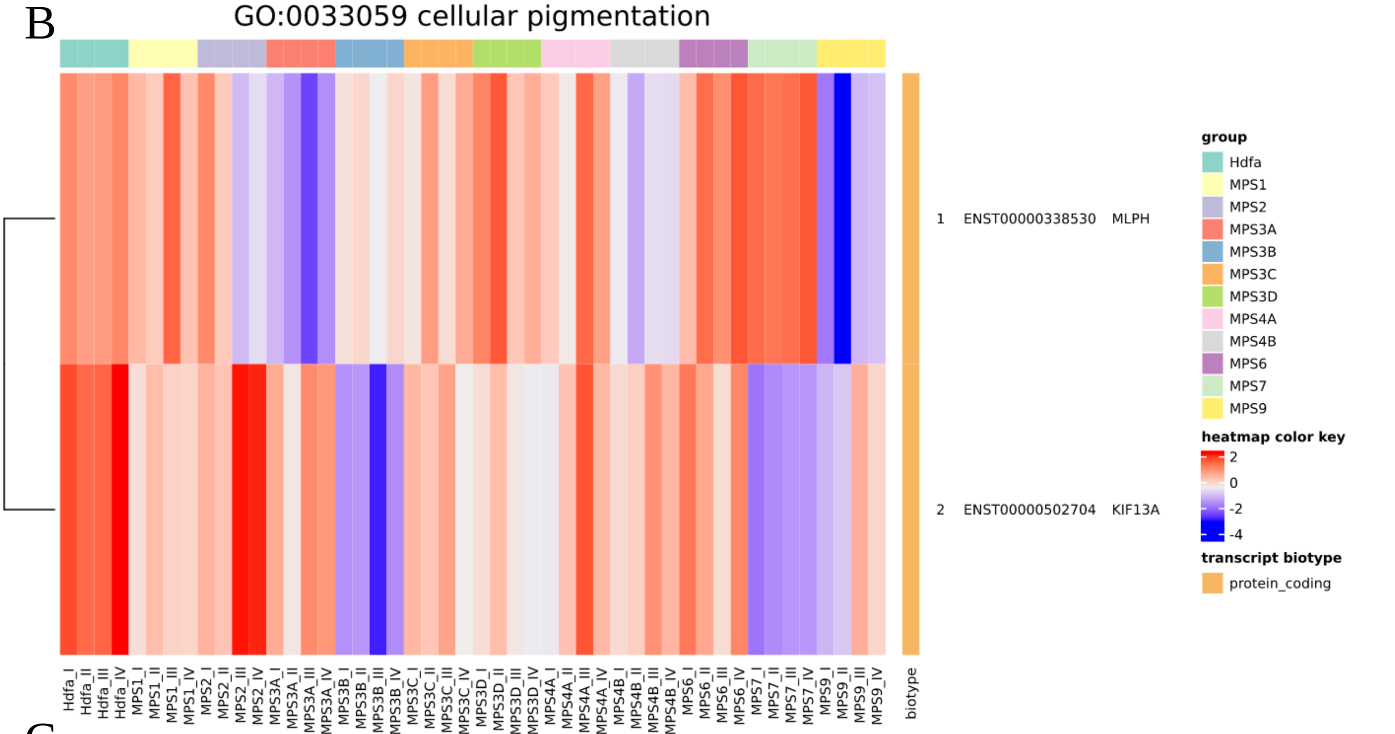

C

Transcripts in **Cellular pigmentation** process	Significant changes in particular MPS type vs HDFa line										
	I	II	IIIA	IIIB	IIIC	IIID	IVA	IVB	VI	VII	IX
Up-regulated (FDR<0.1)	0	1	0	0	0	0	0	0	0	0	0
Up-regulated (FDR<0.000001)	0	0	0	0	0	0	0	0	0	0	0
Down-regulated (FDR<0.1)	3	0	2	4	1	1	2	4	2	2	2
Down-regulated (FDR<0.000001)	1	0	0	2	0	1	0	1	0	1	0

Fig. S2-29

A

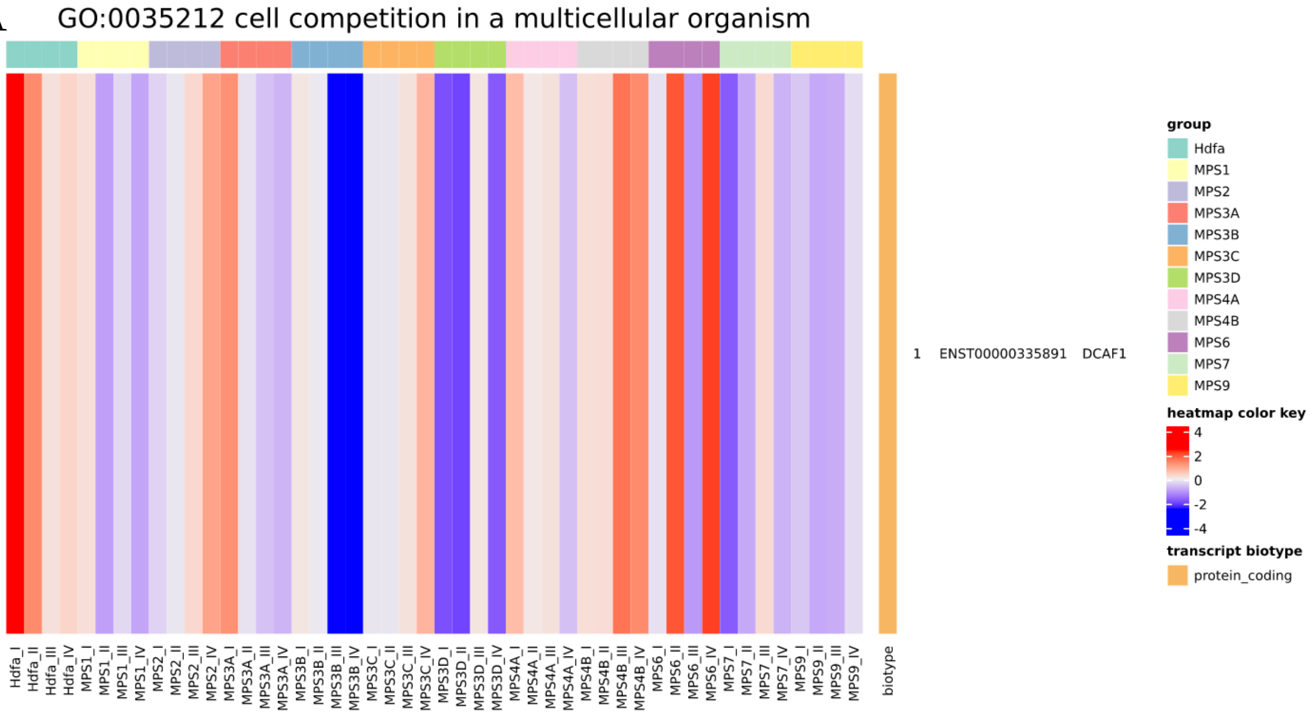

B

Transcripts in **Cell competition in a multicellular organism** process	Significant changes in particular MPS type vs HDFa line										
	I	II	IIIA	IIIB	IIIC	IIID	IVA	IVB	VI	VII	IX
Up-regulated (FDR<0.1)	0	0	0	0	0	0	0	0	0	0	0
Up-regulated (FDR<0.000001)	0	0	0	0	0	0	0	0	0	0	0
Down-regulated (FDR<0.1)	0	0	0	0	0	0	0	0	0	0	0
Down-regulated (FDR<0.000001)	0	0	0	0	0	0	0	0	0	0	0

Fig. S2-30

A

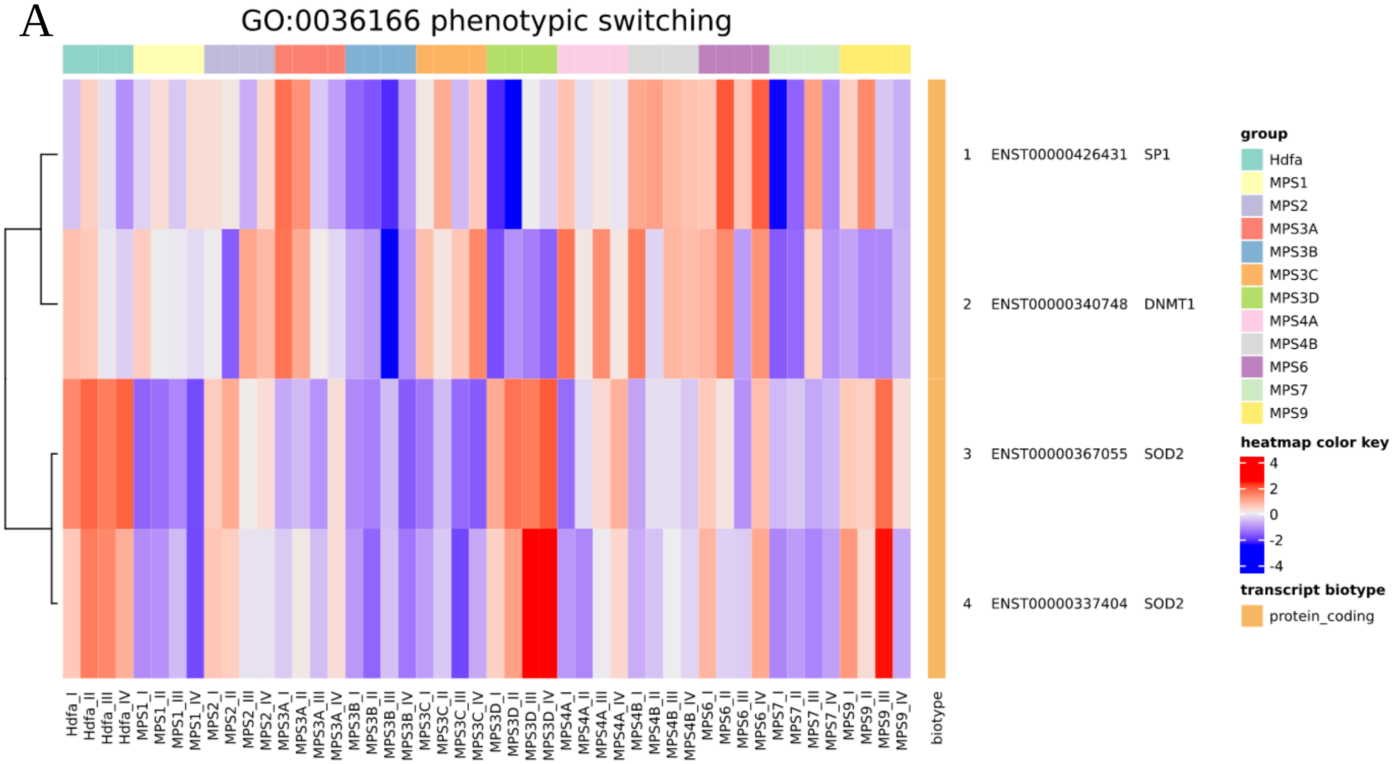

B

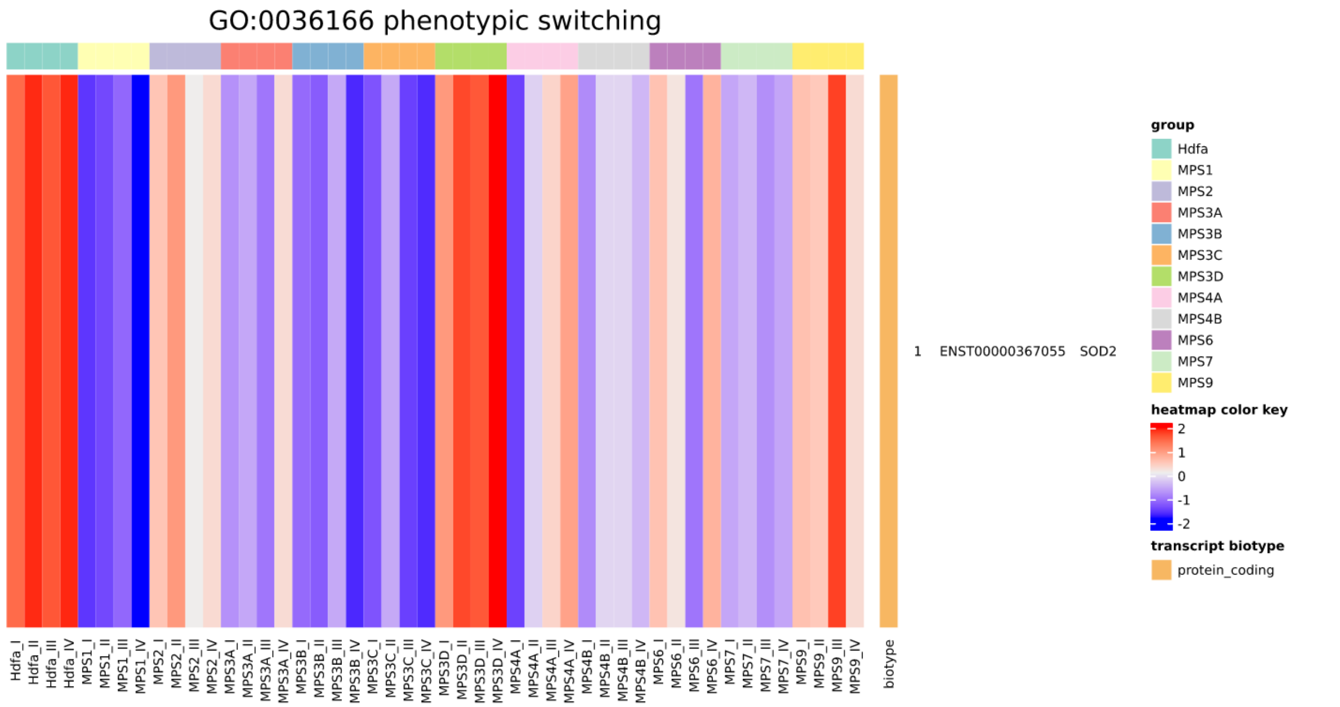

C

Transcripts in **Phenotypic switching** process	Significant changes in particular MPS type vs HDFa line										
	I	II	IIIA	IIIB	IIIC	IIID	IVA	IVB	VI	VII	IX
Up-regulated (FDR<0.1)	0	0	0	0	0	0	0	0	0	0	0
Up-regulated (FDR<0.000001)	0	0	0	0	0	0	0	0	0	0	0
Down-regulated (FDR<0.1)	2	0	0	2	1	0	0	1	0	2	0
Down-regulated (FDR<0.000001)	1	0	0	1	1	0	0	1	0	1	0

Fig. S2-31

A

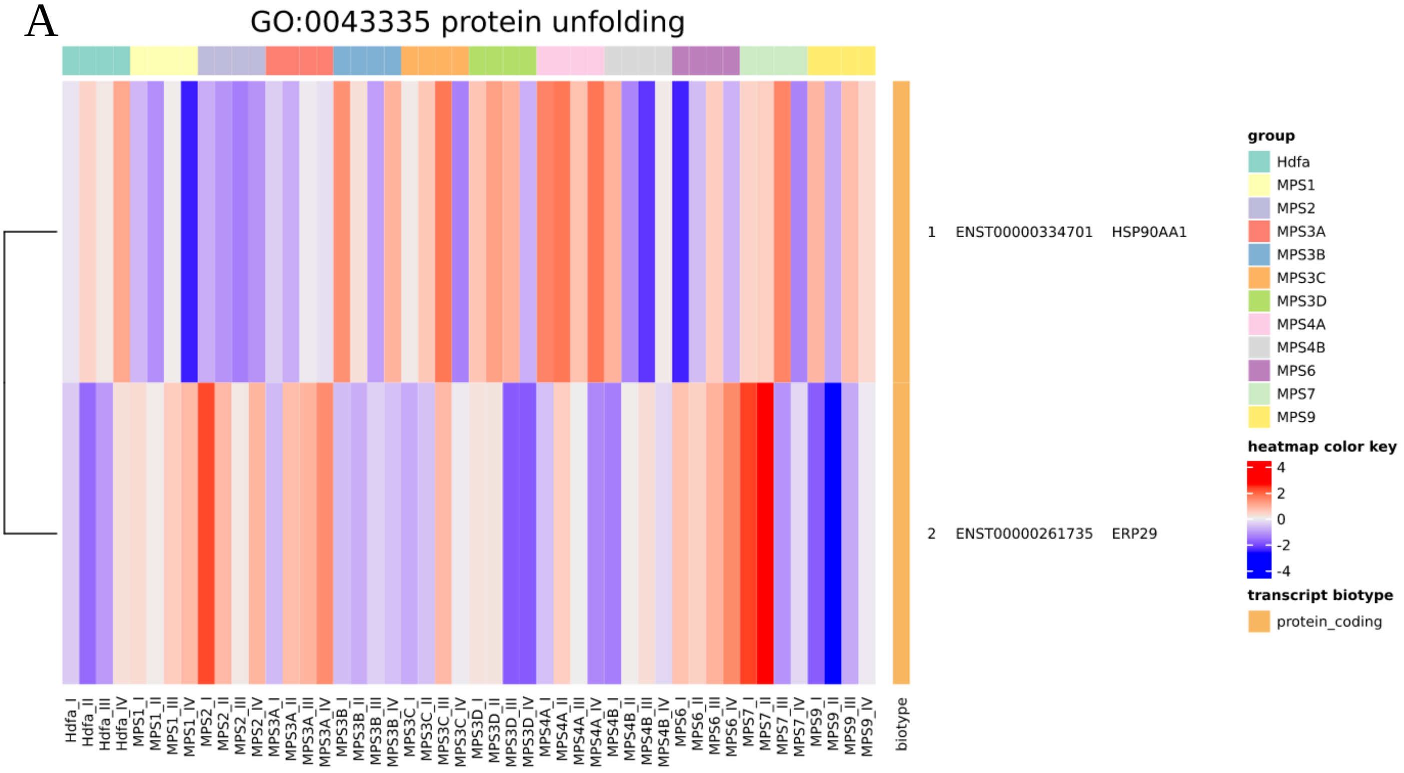

B

Transcripts in **Protein unfolding** process	Significant changes in particular MPS type vs HDFa line										
	I	II	IIIA	IIIB	IIIC	IIID	IVA	IVB	VI	VII	IX
Up-regulated (FDR<0.1)	0	0	0	0	0	0	0	0	0	0	0
Up-regulated (FDR<0.000001)	0	0	0	0	0	0	0	0	0	0	0
Down-regulated (FDR<0.1)	0	0	0	0	0	0	0	0	0	0	0
Down-regulated (FDR<0.000001)	0	0	0	0	0	0	0	0	0	0	0

Fig. S2-32

A

GO:0044237 cellular metabolic process

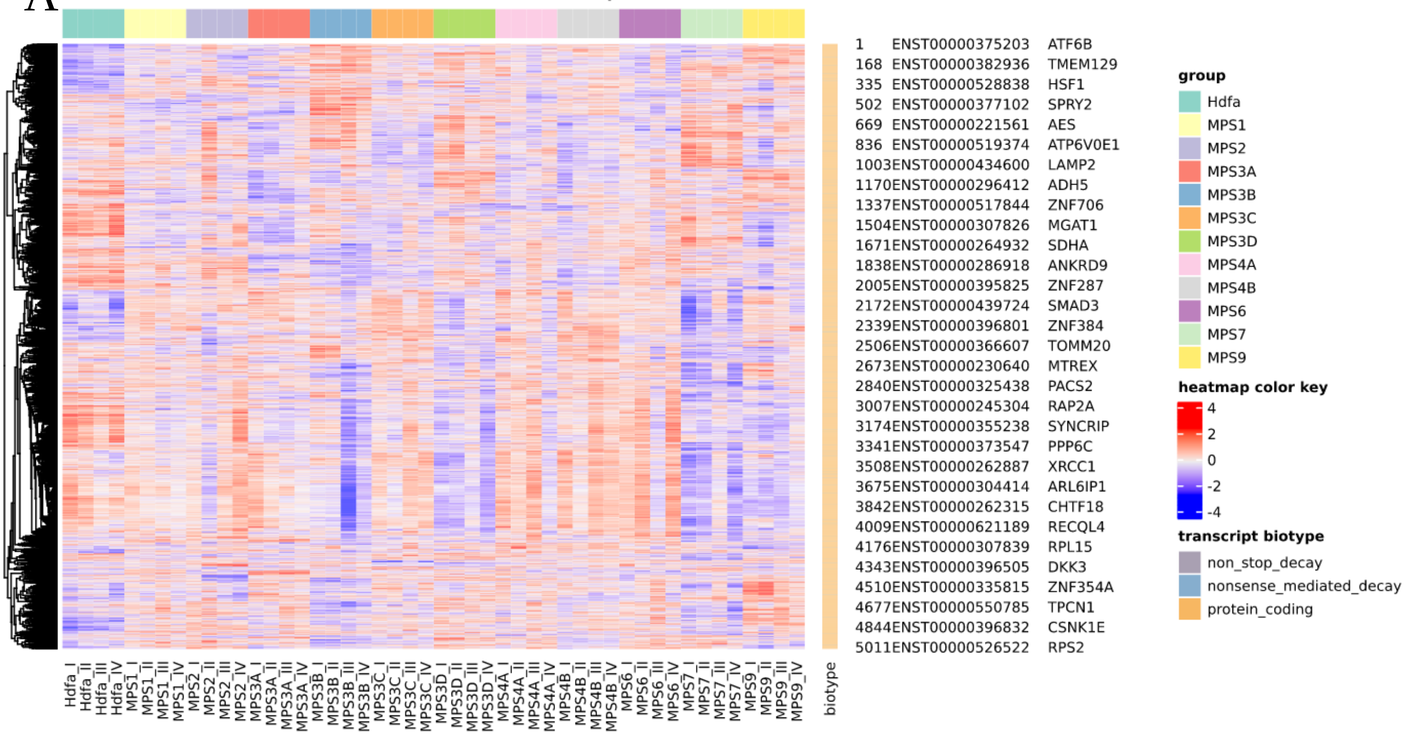

B

GO:0044237 cellular metabolic process

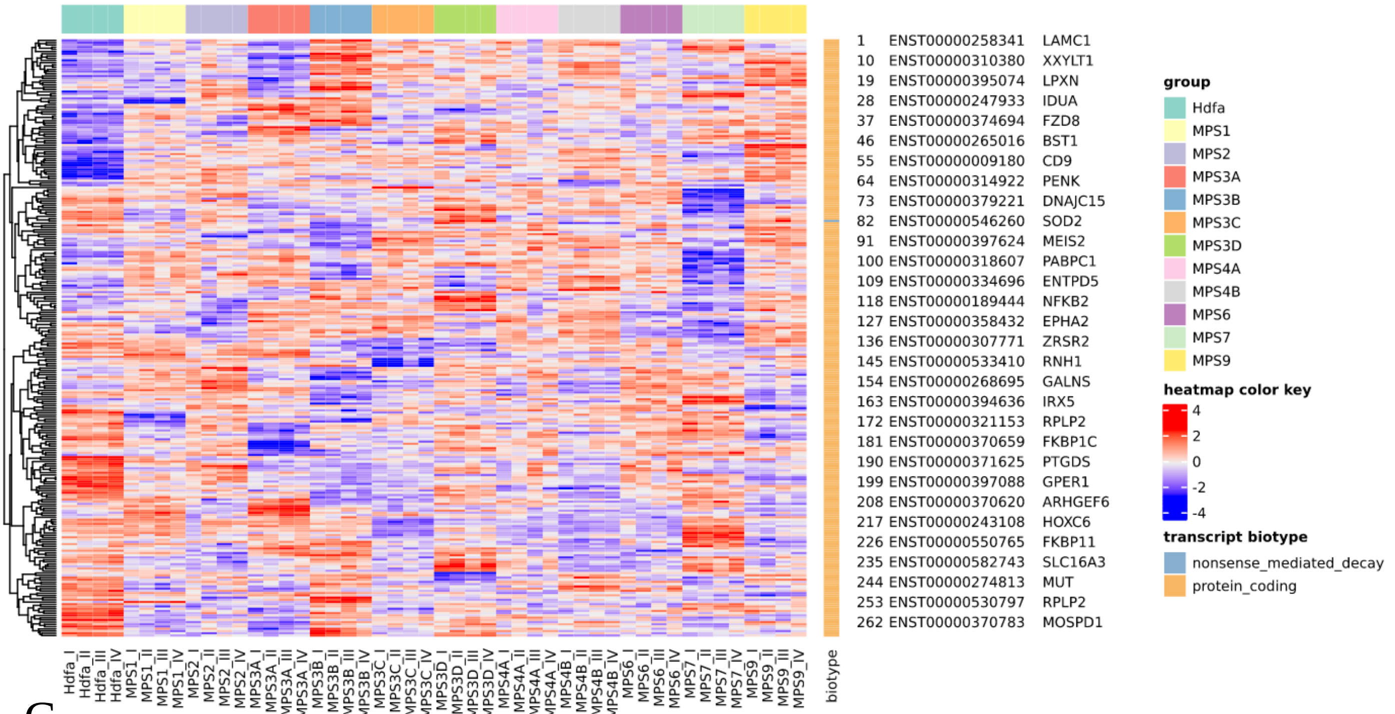

C

Transcripts in **Cellular metabolic process**	Significant changes in particular MPS type vs HDFa line										
	I	II	IIIA	IIIB	IIIC	IIID	IVA	IVB	VI	VII	IX
Up-regulated (FDR<0.1)	132	96	148	185	148	126	61	205	74	158	179
Up-regulated (FDR<0.000001)	37	30	43	50	37	34	16	50	20	39	52
Down-regulated (FDR<0.1)	213	67	264	226	208	217	89	221	71	250	285
Down-regulated (FDR<0.000001)	44	18	49	50	56	38	21	54	21	56	47

Fig. S2-33

A

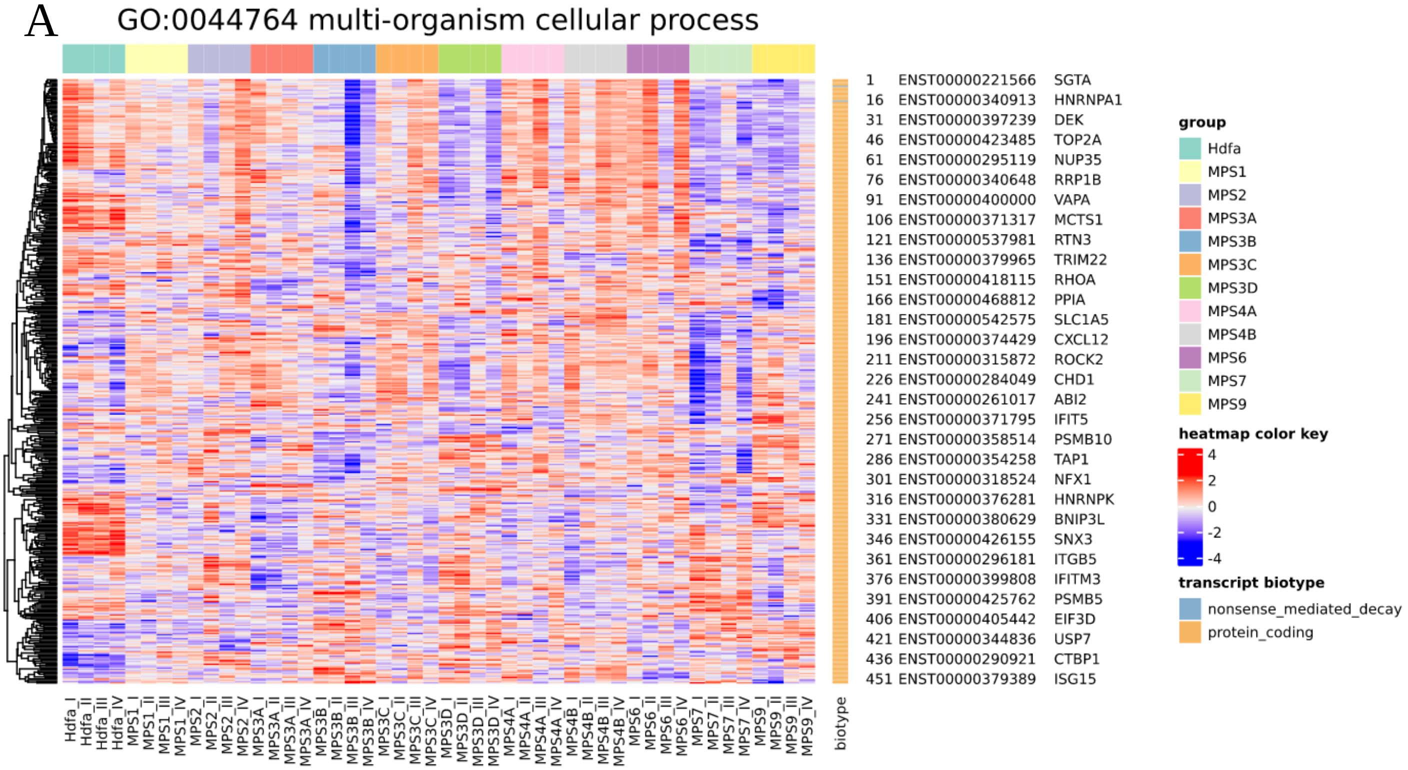

B

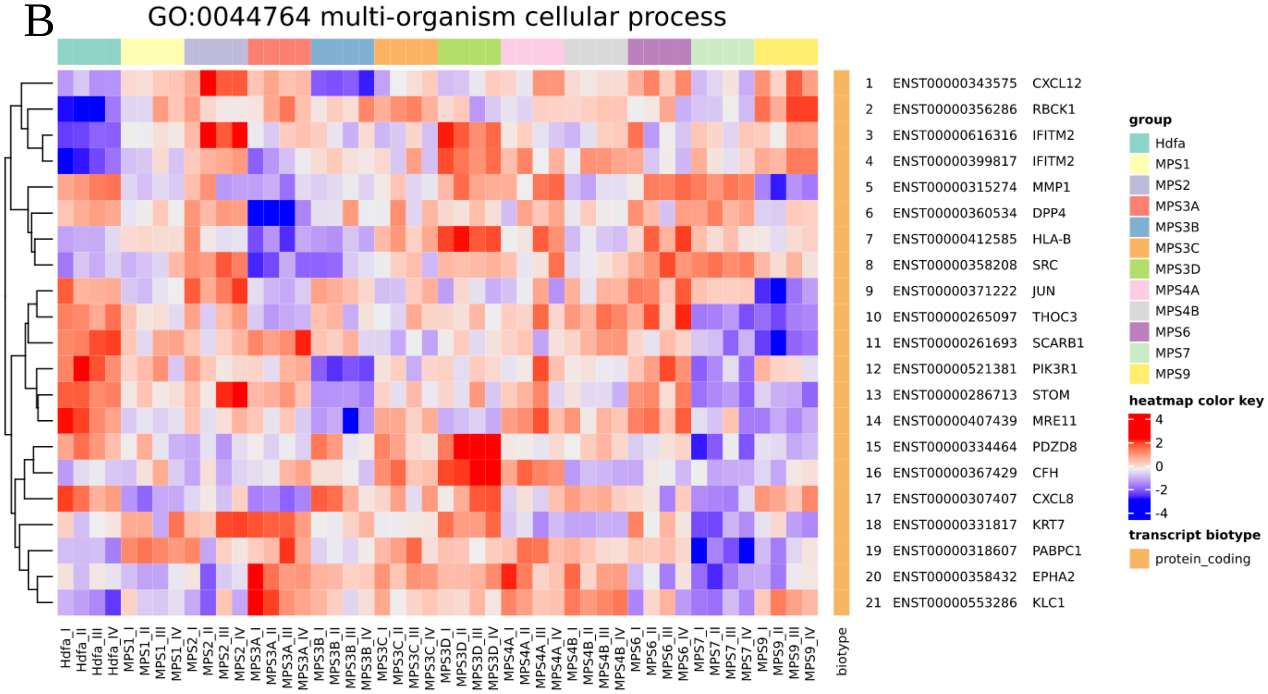

C

Transcripts in **Multi-organism cellular process**	Significant changes in particular MPS type vs HDFa line										
	I	II	IIIA	IIIB	IIIC	IIID	IVA	IVB	VI	VII	IX
Up-regulated (FDR<0.1)	12	8	15	16	15	12	9	18	6	14	17
Up-regulated (FDR<0.000001)	3	3	4	2	2	4	3	1	2	4	4
Down-regulated (FDR<0.1)	21	9	25	22	13	28	10	24	8	27	26
Down-regulated (FDR<0.000001)	3	1	3	4	1	1	0	2	1	5	6

Fig. S2-34

A

GO:0045103 intermediate filament-based process

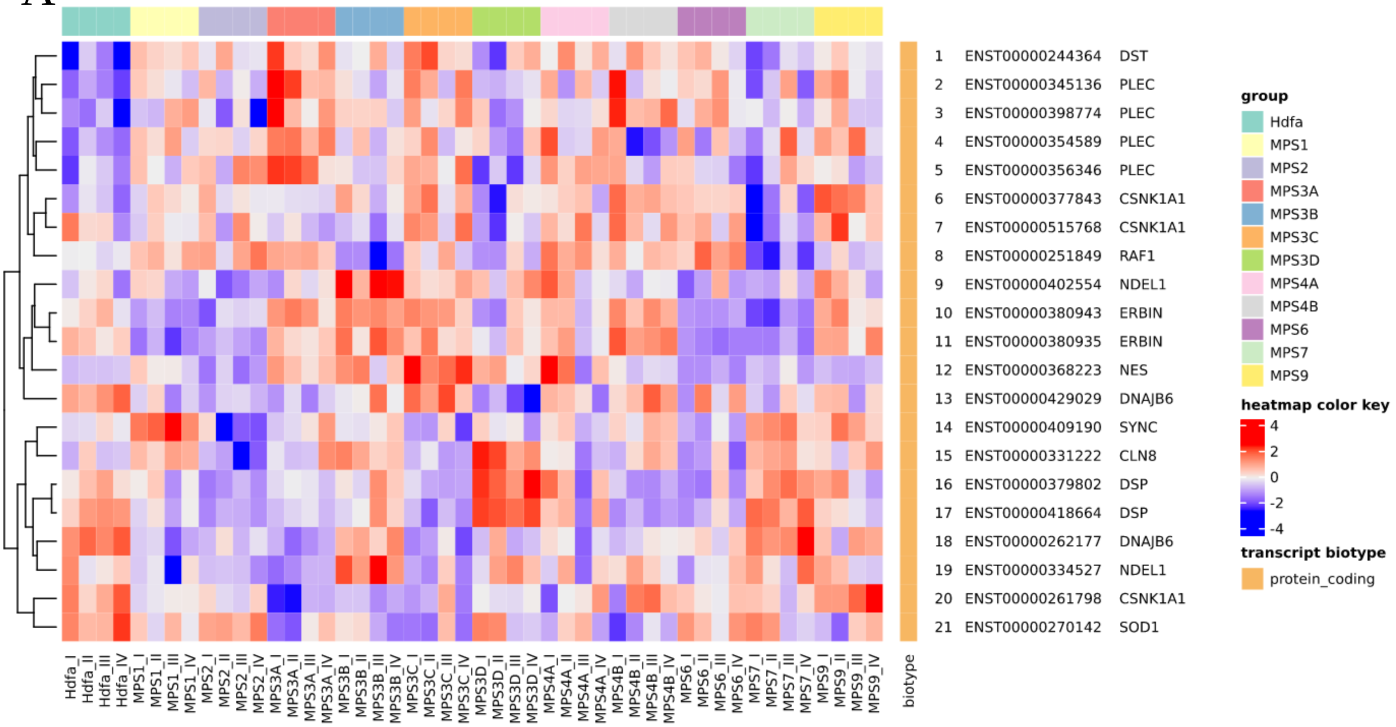

B

GO:0045103 intermediate filament-based process

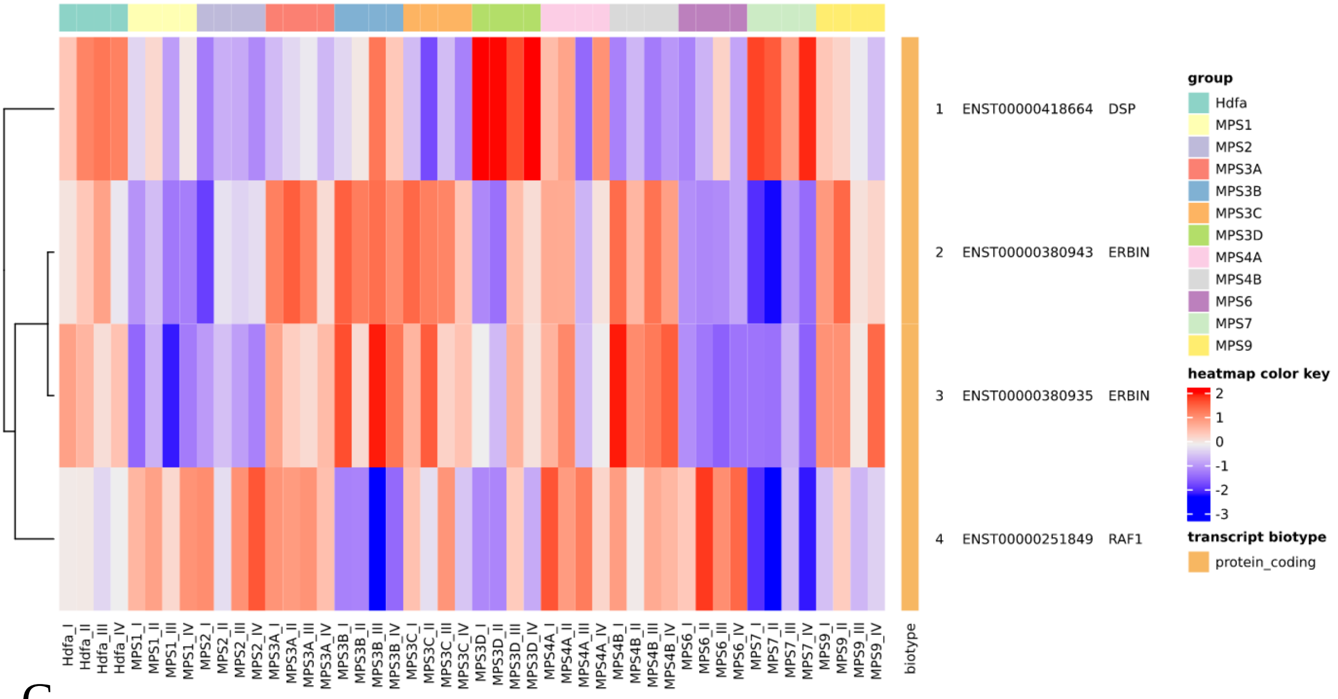

C

Transcripts in **Intermediate filament-based process**	Significant changes in particular MPS type vs HDFa line										
	I	II	IIIA	IIIB	IIIC	IIID	IVA	IVB	VI	VII	IX
Up-regulated (FDR<0.1)	1	0	2	0	0	0	0	0	0	0	0
Up-regulated (FDR<0.000001)	0	0	1	0	0	0	0	0	0	0	0
Down-regulated (FDR<0.1)	0	5	2	0	1	0	0	2	1	1	0
Down-regulated (FDR<0.000001)	0	2	0	0	1	0	0	1	1	1	0

Fig. S2-35

A GO:0048522 positive regulation of cellular process

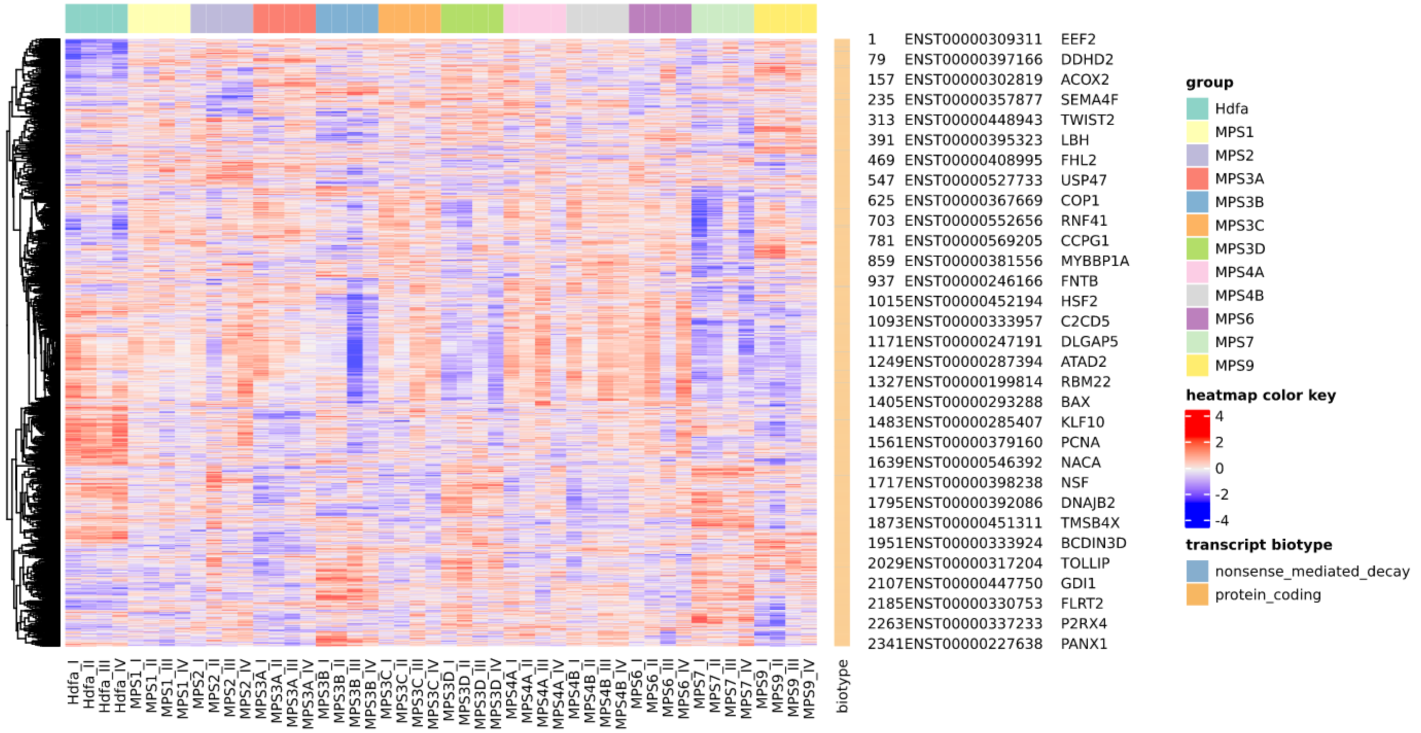

B GO:0048522 positive regulation of cellular process

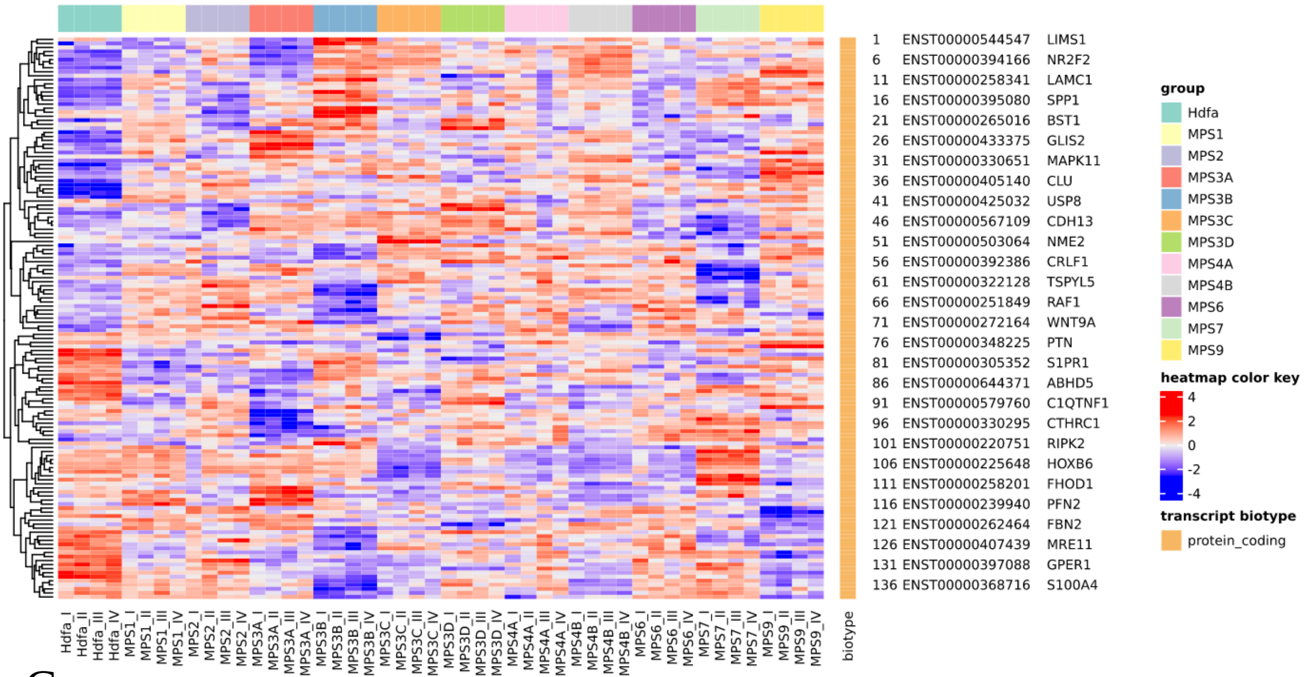

C

Transcripts in **Positive regulation of cellular process**	Significant changes in particular MPS type vs HDFa line										
	I	II	IIIA	IIIB	IIIC	IIID	IVA	IVB	VI	VII	IX
Up-regulated (FDR<0.1)	77	50	94	109	86	85	37	131	37	96	105
Up-regulated (FDR<0.000001)	21	14	32	28	23	23	12	32	12	26	34
Down-regulated (FDR<0.1)	96	26	112	108	100	93	47	130	37	120	134
Down-regulated (FDR<0.000001)	16	6	17	23	26	18	9	29	7	19	25

Fig. S2-36

A
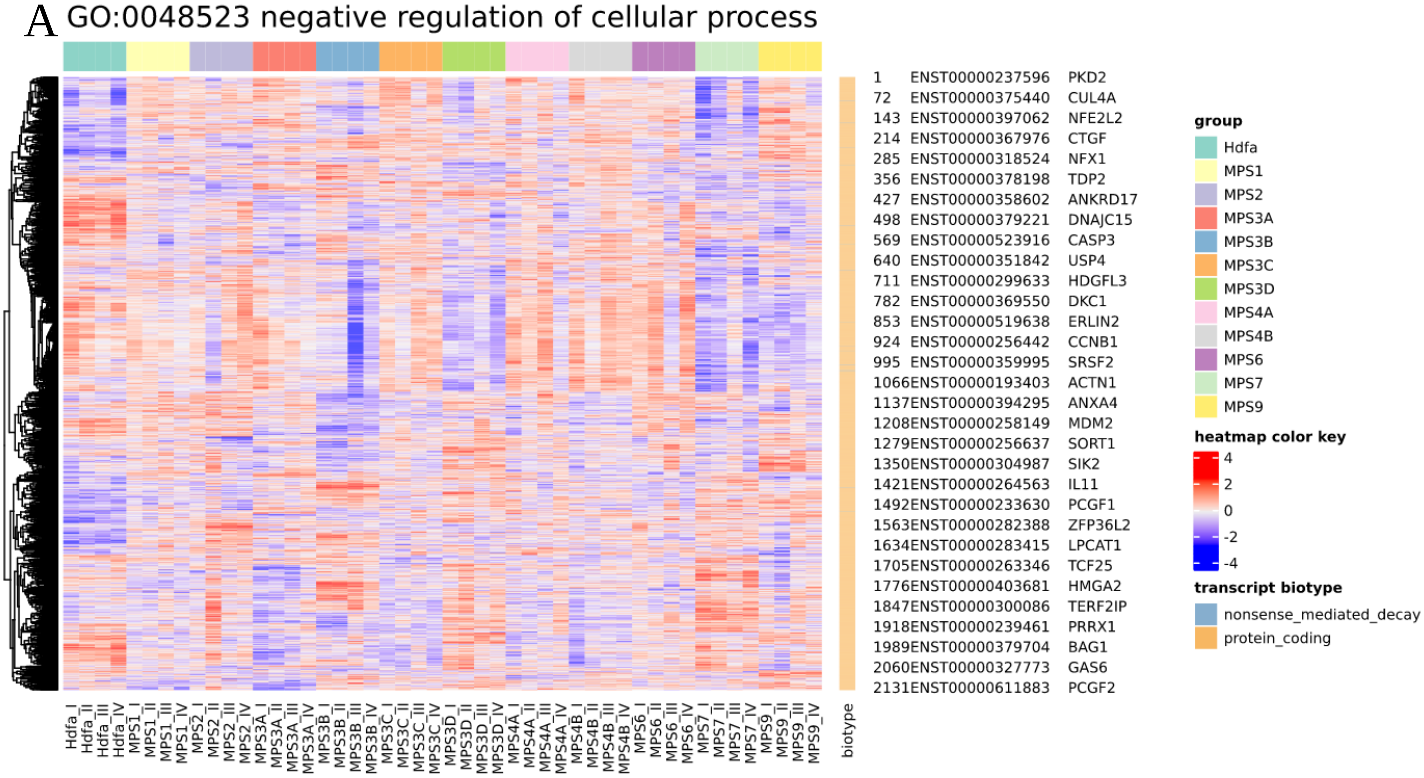

B
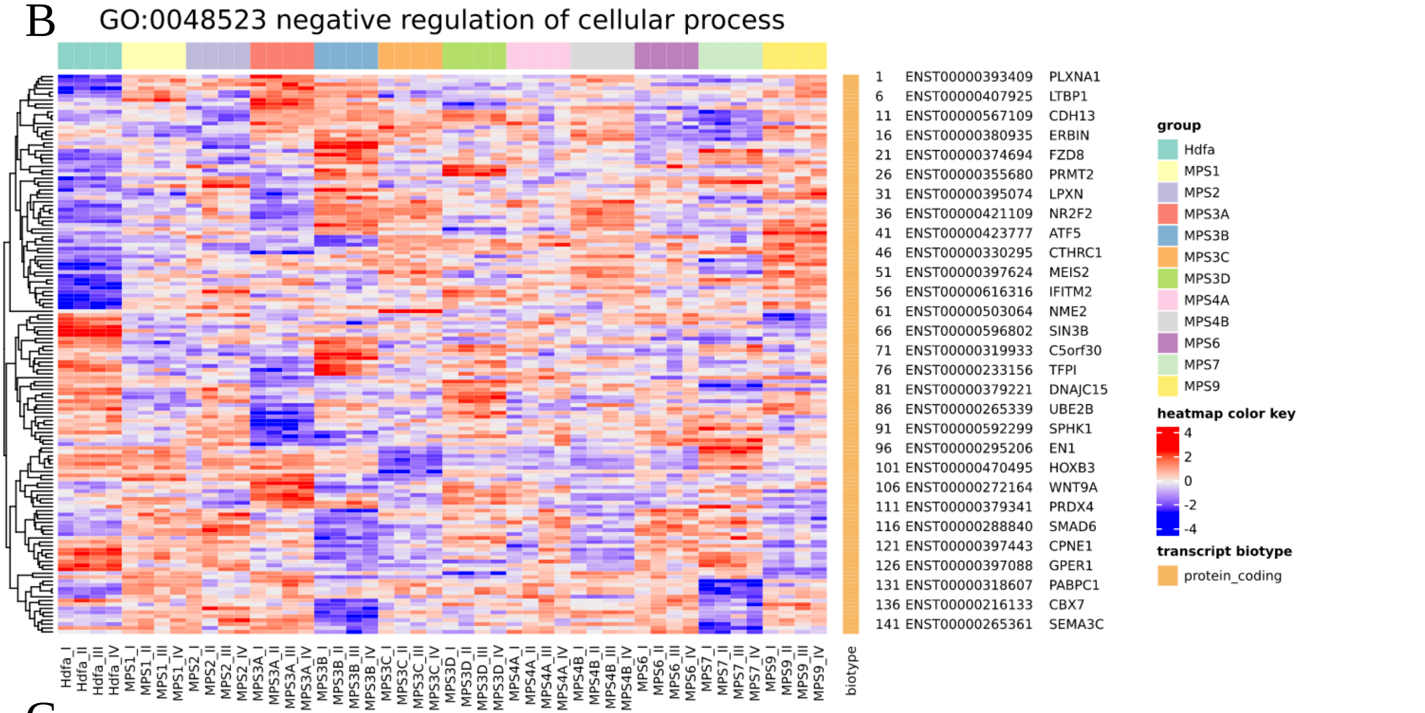

C

Transcripts in **Negative regulation of cellular process**	Significant changes in particular MPS type vs HDFa line										
	I	II	IIIA	IIIB	IIIC	IIID	IVA	IVB	VI	VII	IX
Up-regulated (FDR<0.1)	75	54	84	108	84	81	35	116	45	100	104
Up-regulated (FDR<0.000001)	26	21	34	32	25	24	14	34	18	25	37
Down-regulated (FDR<0.1)	87	31	119	104	81	90	37	110	35	104	117
Down-regulated (FDR<0.000001)	15	8	19	20	23	16	8	25	8	24	19

Fig. S2-37

A

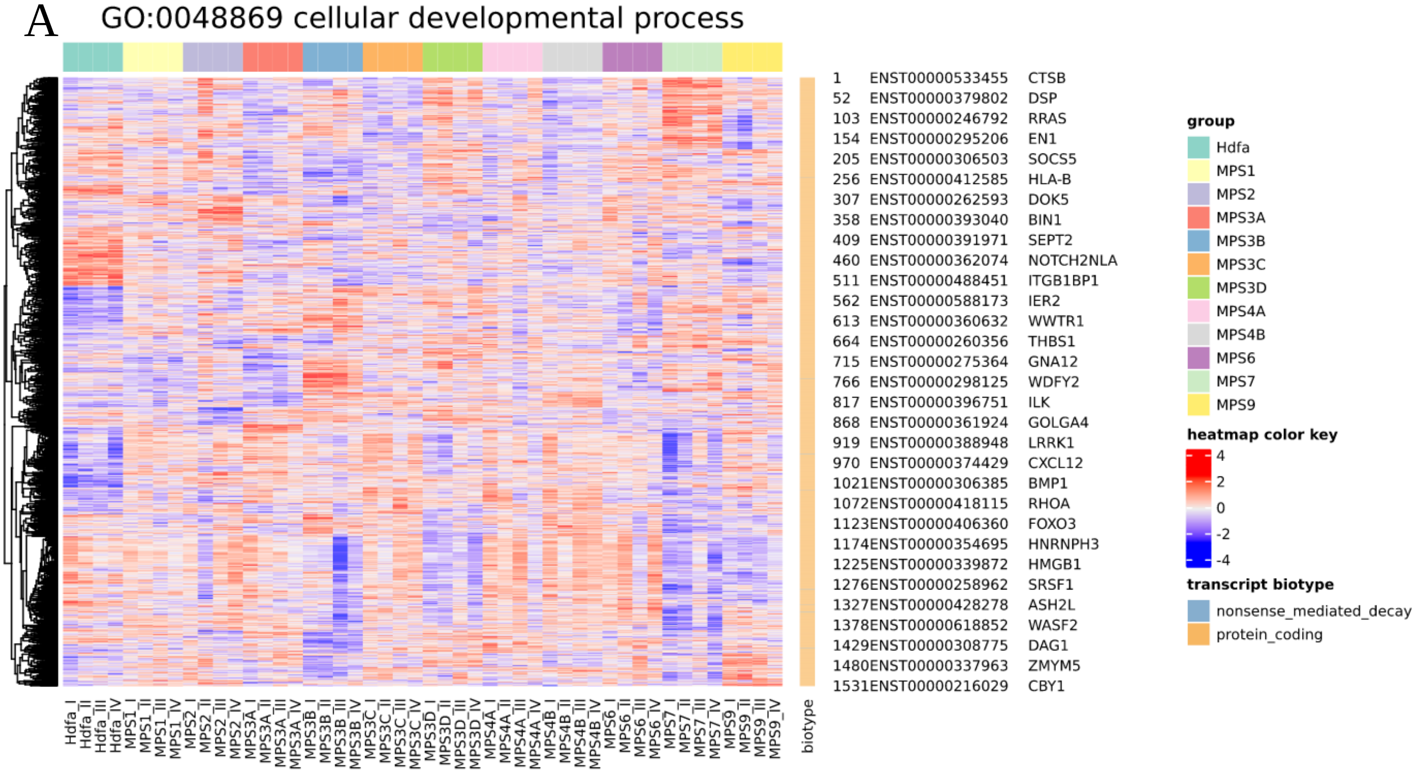

B

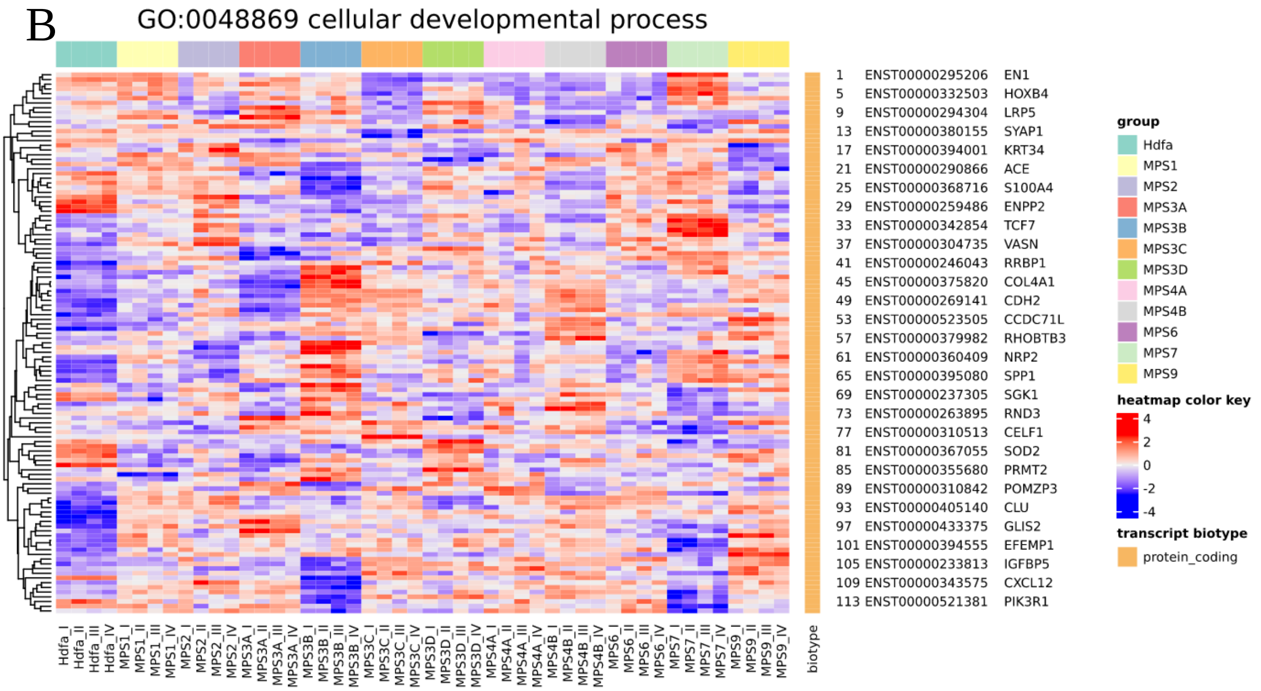

C

Transcripts in **Cellular developmental process**	Significant changes in particular MPS type vs HDFa line										
	I	II	IIIA	IIIB	IIIC	IIID	IVA	IVB	VI	VII	IX
Up-regulated (FDR<0.1)	61	37	77	85	85	54	26	104	32	98	83
Up-regulated (FDR<0.000001)	22	12	25	23	26	17	10	37	14	28	30
Down-regulated (FDR<0.1)	59	23	84	67	67	46	33	82	26	62	77
Down-regulated (FDR<0.000001)	9	4	14	17	18	11	8	15	4	14	12

Fig. S2-38

A

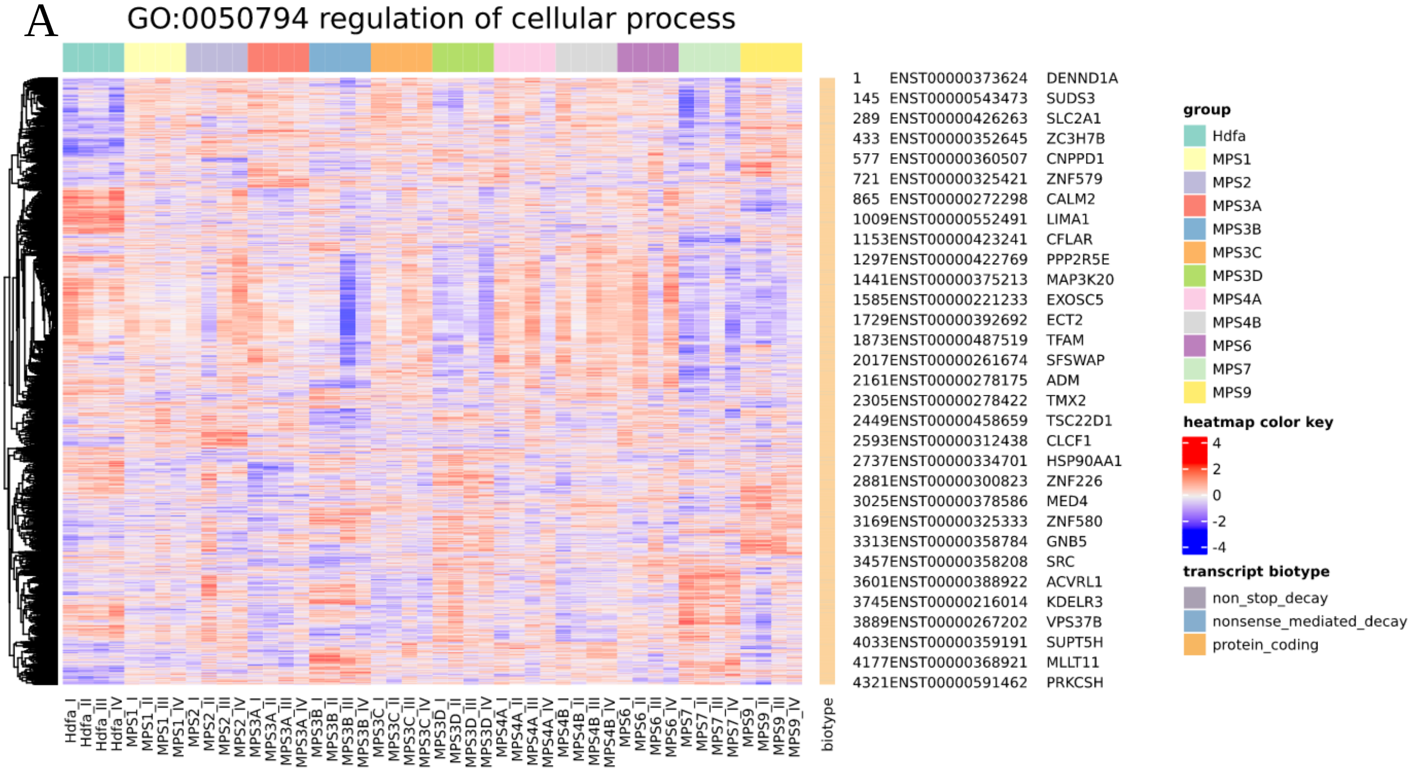

B

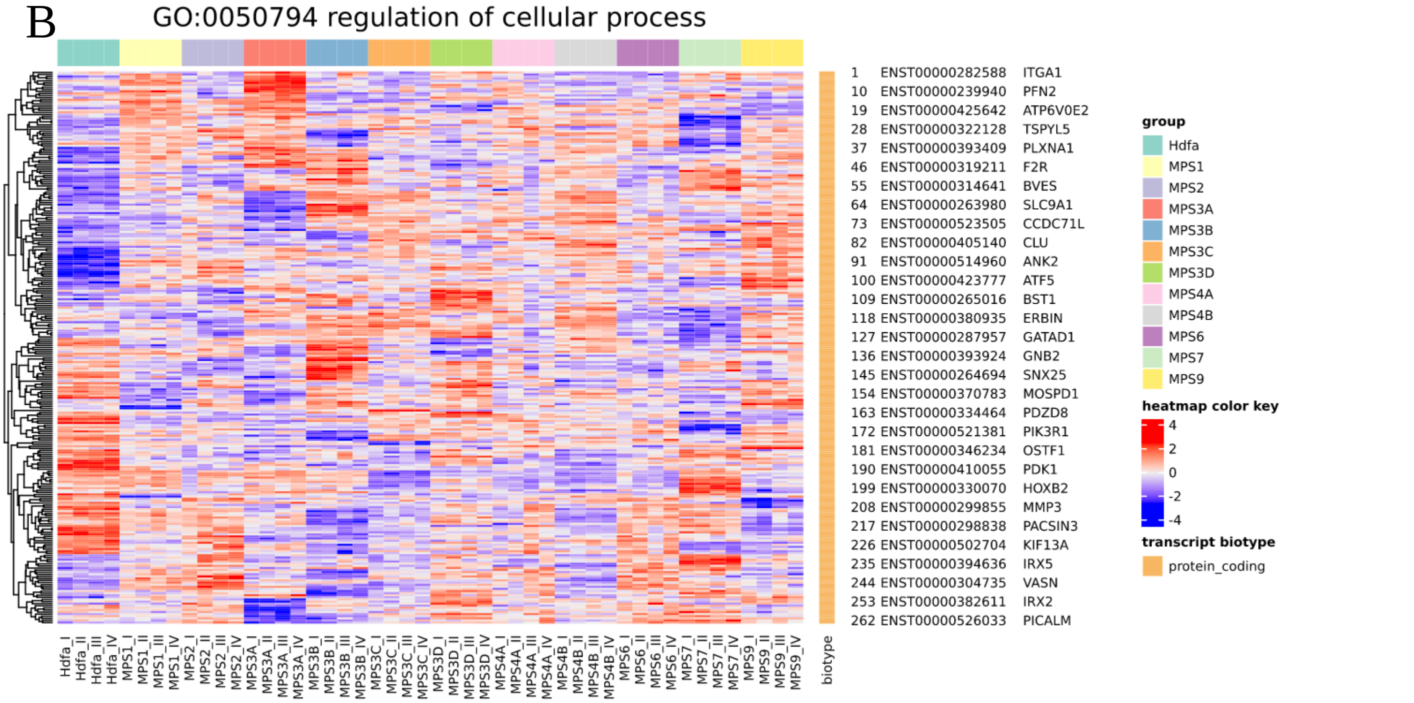

C

Transcripts in **Regulation of cellular process**	Significant changes in particular MPS type vs HDFa line										
	I	II	IIIA	IIIB	IIIC	IIID	IVA	IVB	VI	VII	IX
Up-regulated (FDR<0.1)	140	95	170	206	165	143	75	219	75	185	193
Up-regulated (FDR<0.000001)	48	31	54	51	45	43	22	59	25	52	59
Down-regulated (FDR<0.1)	179	67	241	190	168	161	74	209	60	199	233
Down-regulated (FDR<0.000001)	35	21	44	44	46	31	18	54	16	49	41

Fig. S2-39

A

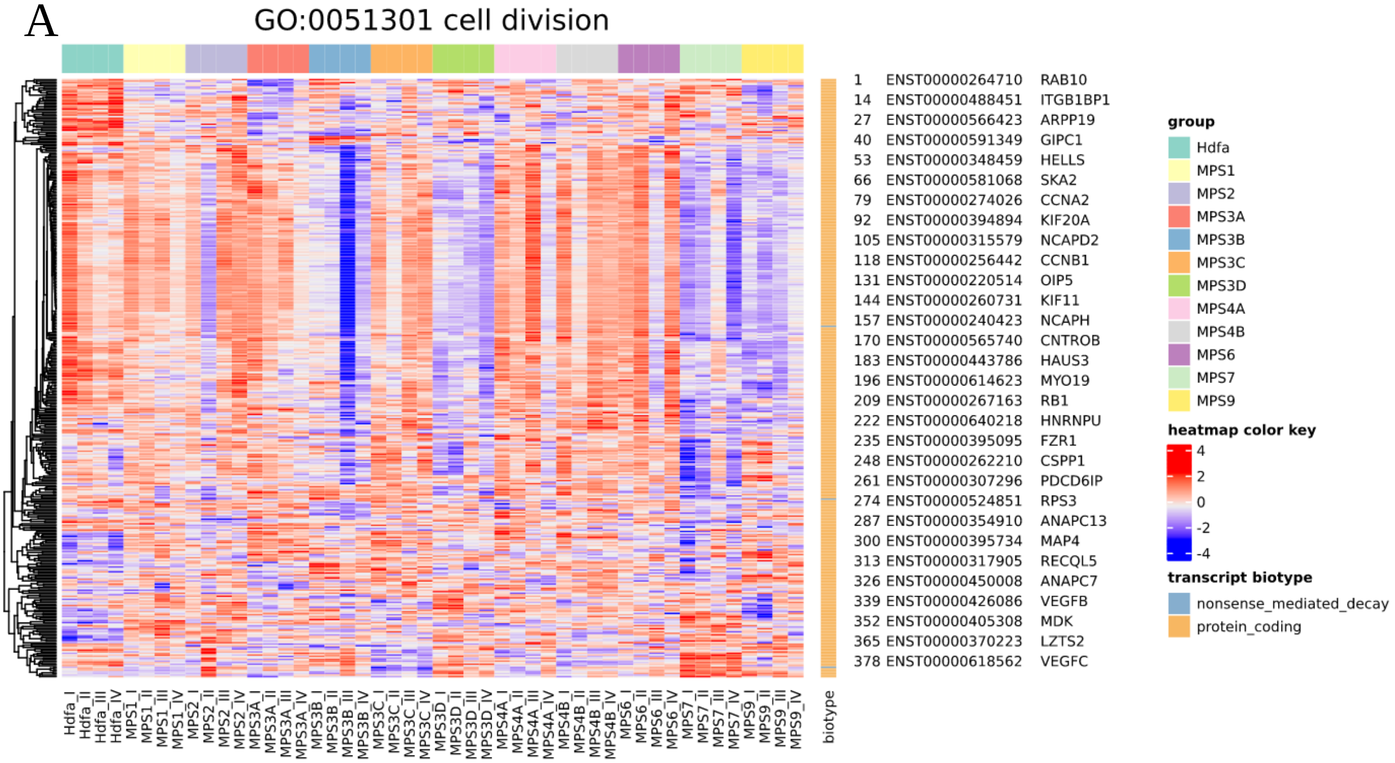

B

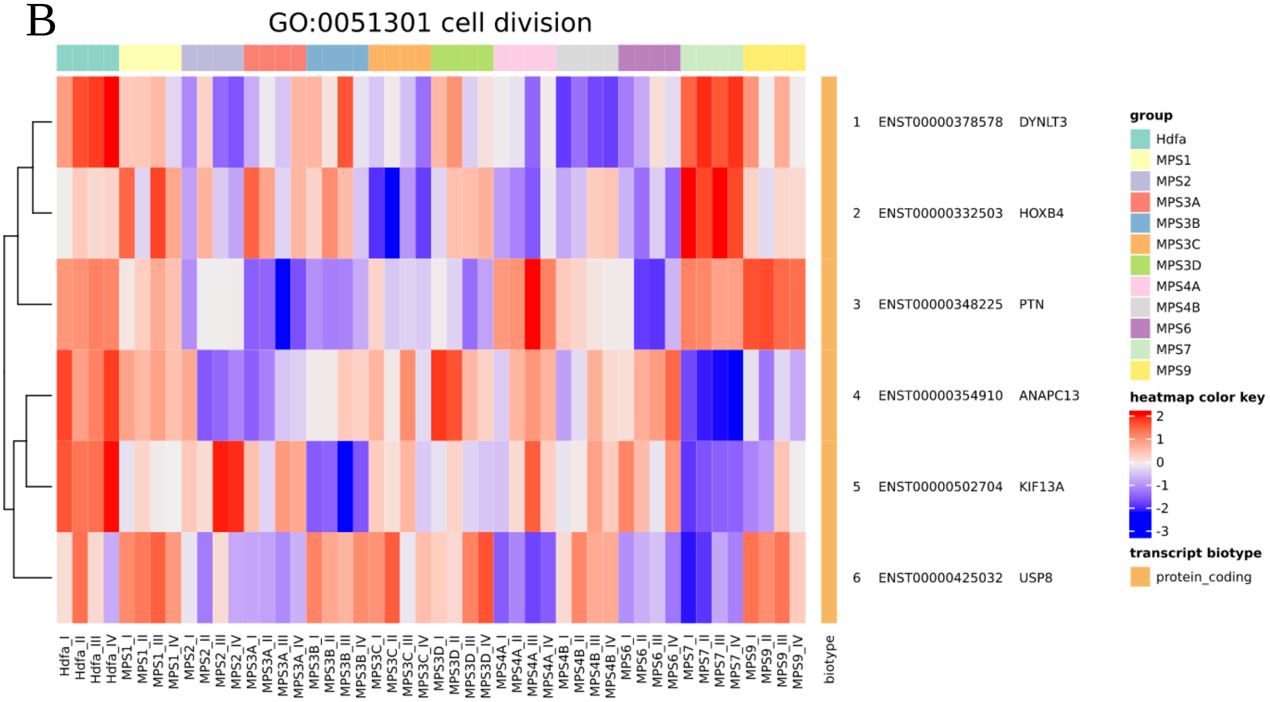

C

Transcripts in **Cell division** process	Significant changes in particular MPS type vs HDFa line										
	I	II	IIIA	IIIB	IIIC	IIID	IVA	IVB	VI	VII	IX
Up-regulated (FDR<0.1)	9	2	7	6	6	5	2	10	1	10	2
Up-regulated (FDR<0.000001)	0	0	0	0	0	0	0	0	0	1	0
Down-regulated (FDR<0.1)	11	6	8	14	11	19	0	10	1	21	24
Down-regulated (FDR<0.000001)	1	0	2	2	1	1	0	2	0	2	1

Fig. S2-40

A

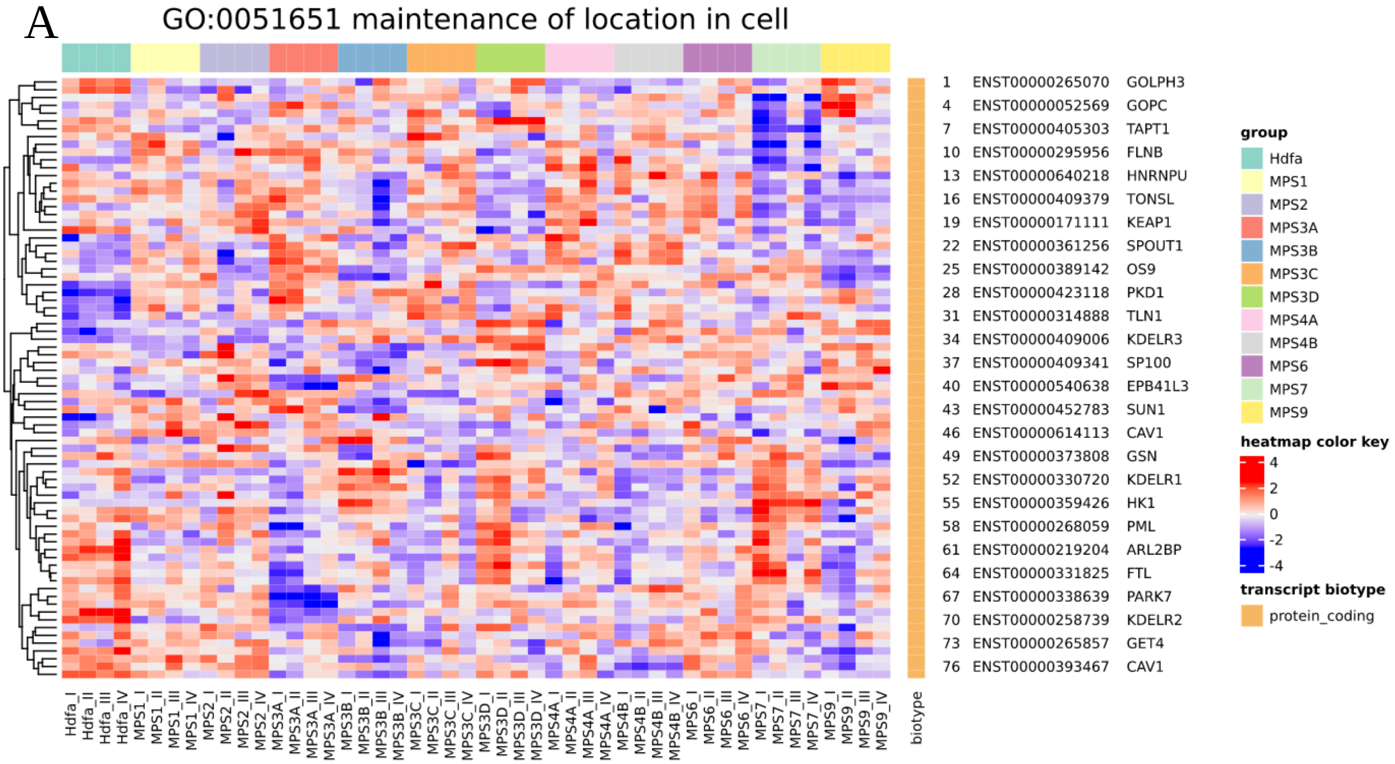

B

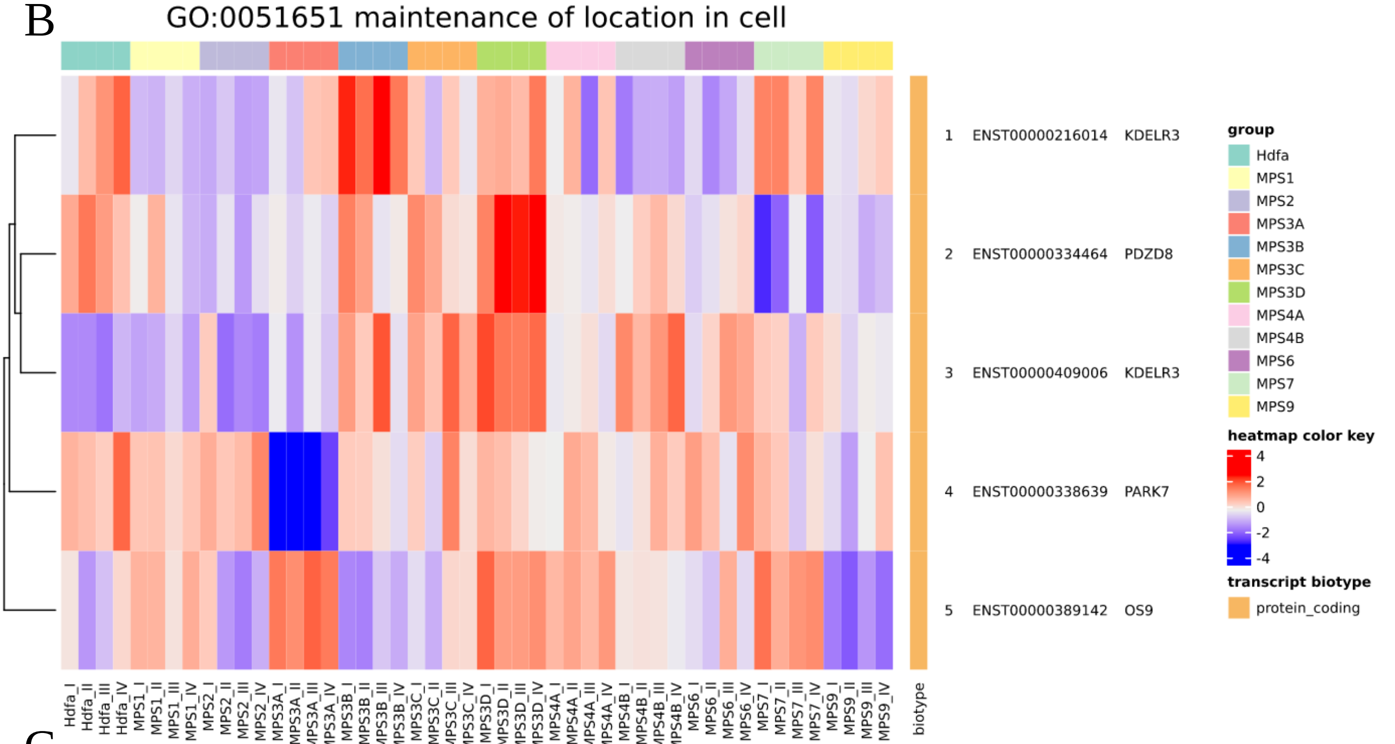

C

Transcripts in **Maintenance of location in cell** process	Significant changes in particular MPS type vs HDFa line										
	I	II	IIIA	IIIB	IIIC	IIID	IVA	IVB	VI	VII	IX
Up-regulated (FDR<0.1)	2	0	0	1	5	2	0	3	0	2	0
Up-regulated (FDR<0.000001)	0	0	0	0	1	1	0	1	0	0	0
Down-regulated (FDR<0.1)	3	2	5	4	1	0	3	7	0	2	4
Down-regulated (FDR<0.000001)	0	1	1	0	0	0	0	0	0	0	1

Fig. S2-41

A

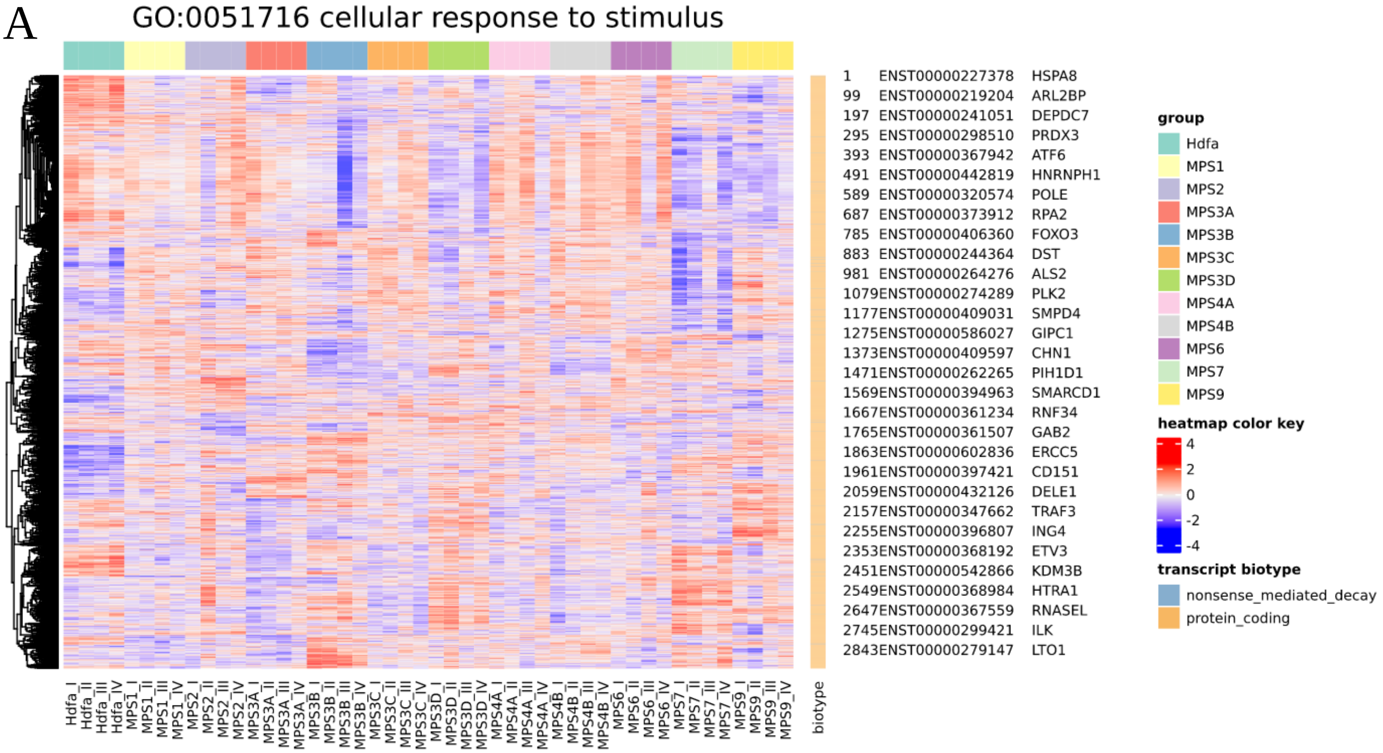

B

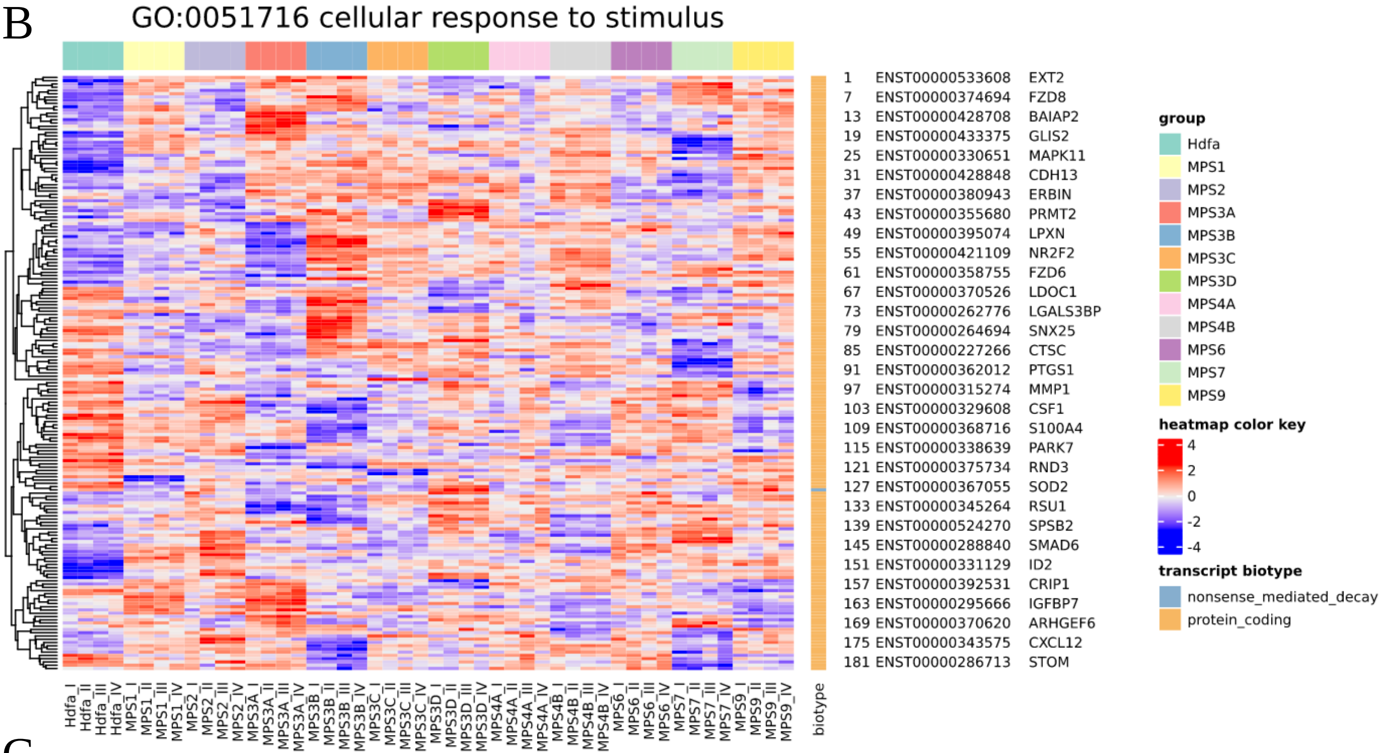

C

Transcripts in **Cellular response to stimulus** process	Significant changes in particular MPS type vs HDFa line										
	I	II	IIIA	IIIB	IIIC	IIID	IVA	IVB	VI	VII	IX
Up-regulated (FDR<0.1)	102	64	127	147	113	107	43	153	55	126	142
Up-regulated (FDR<0.000001)	36	22	44	42	37	36	16	46	23	31	46
Down-regulated (FDR<0.1)	137	39	165	136	118	126	51	148	45	140	162
Down-regulated (FDR<0.000001)	23	11	28	28	22	15	9	27	1	29	22

Fig. S2-42

A

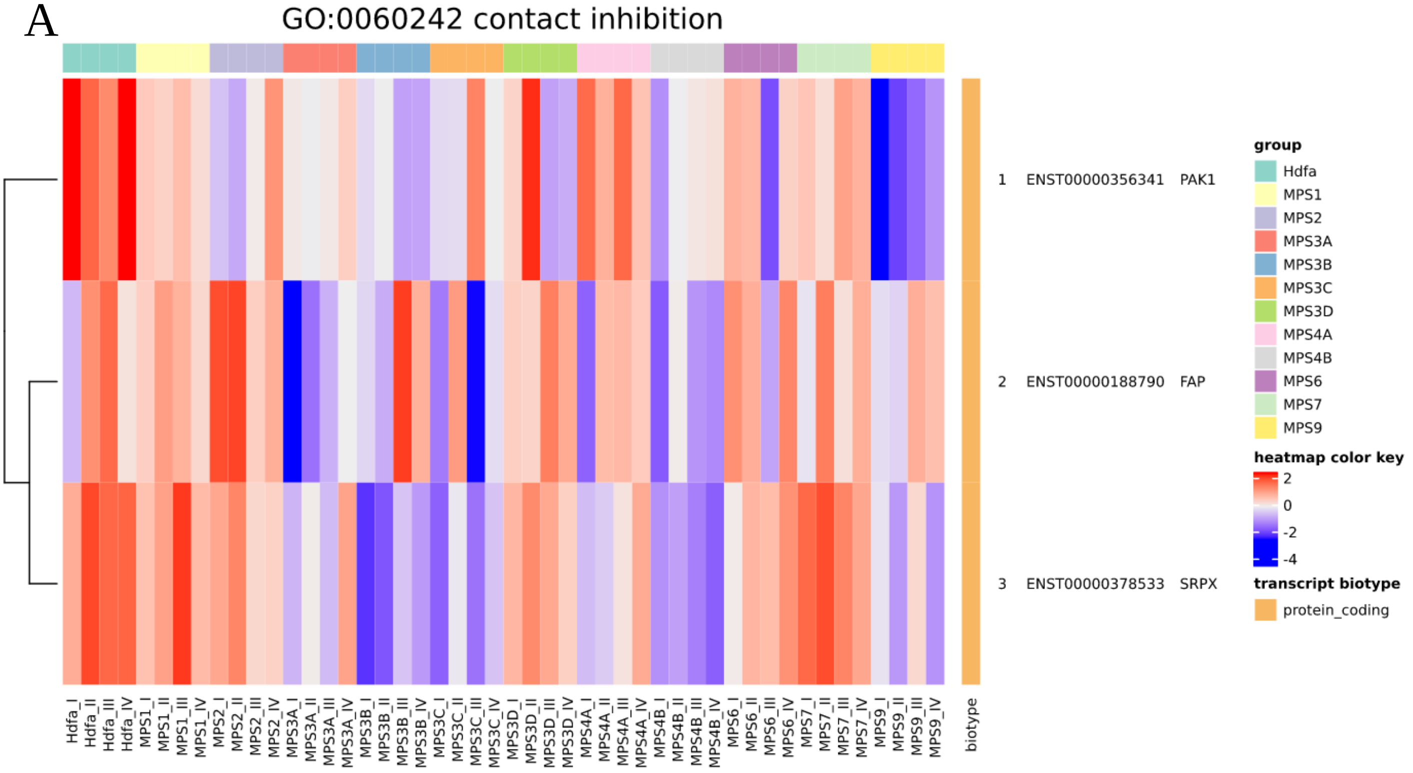

B

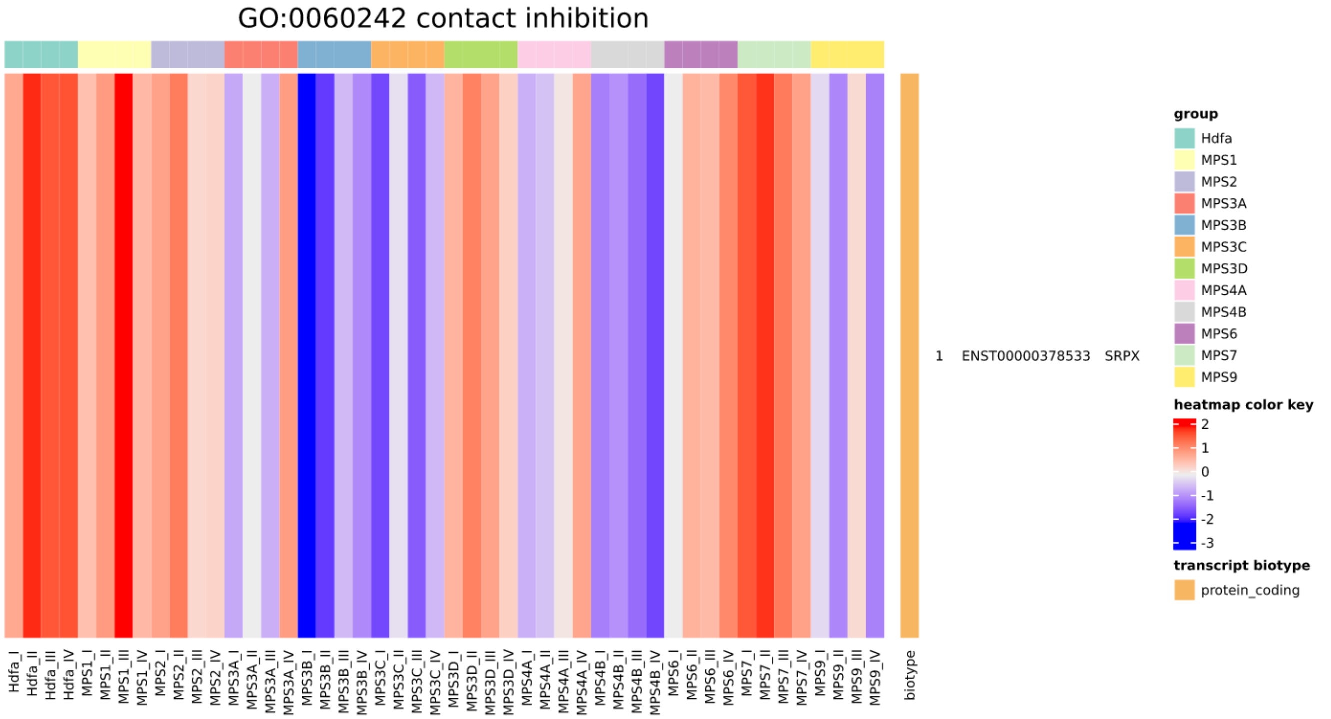

C

Transcripts in **Contact inhibition** process	Significant changes in particular MPS type vs HDFa line										
	I	II	IIIA	IIIB	IIIC	IIID	IVA	IVB	VI	VII	IX
Up-regulated (FDR<0.1)	0	0	0	0	0	0	0	0	0	0	0
Up-regulated (FDR<0.000001)	0	0	0	0	0	0	0	0	0	0	0
Down-regulated (FDR<0.1)	0	0	0	1	0	0	0	1	0	0	1
Down-regulated (FDR<0.000001)	0	0	0	1	0	0	0	1	0	0	0

Fig. S2-43

A

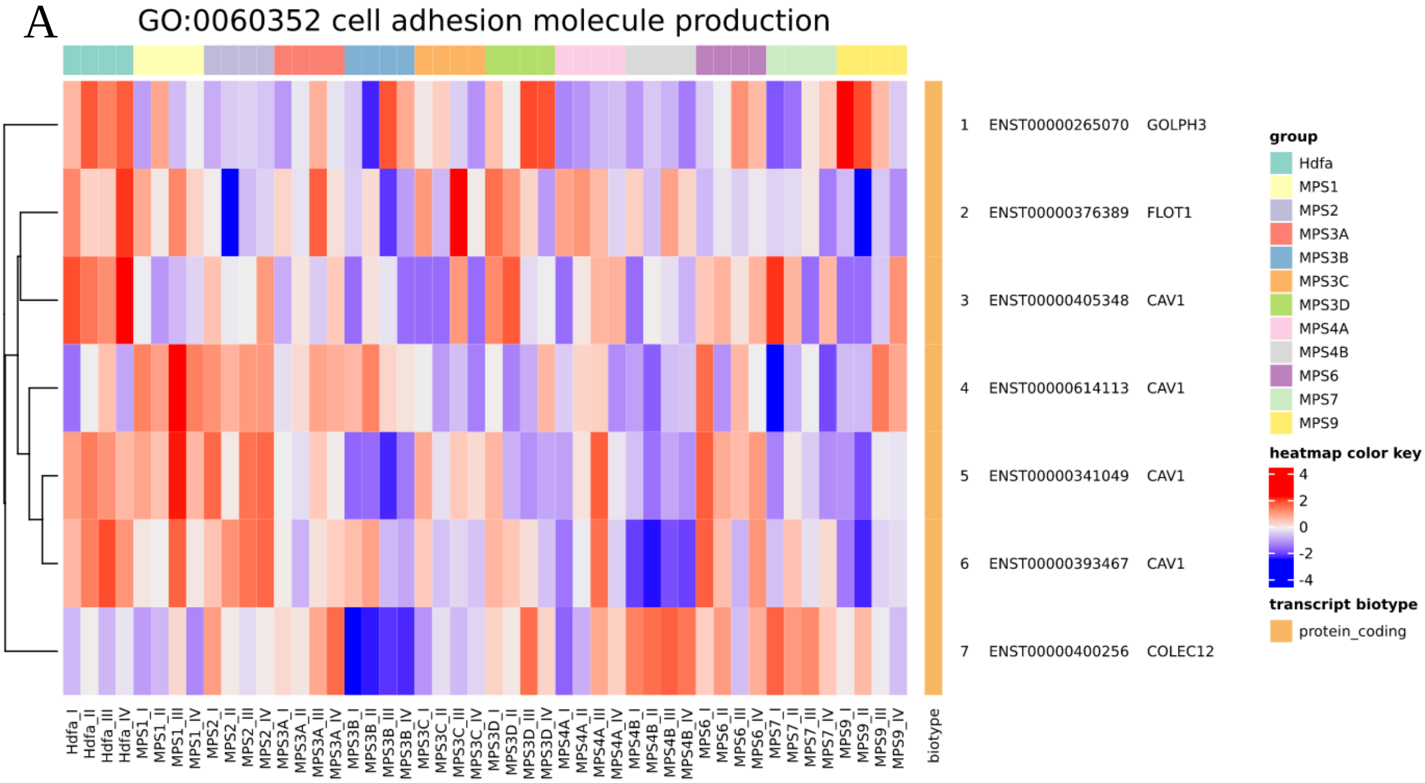

B

Transcripts in **Cell adhesion molecule production** process	Significant changes in particular MPS type vs HDFa line										
	I	II	IIIA	IIIB	IIIC	IIID	IVA	IVB	VI	VII	IX
Up-regulated (FDR<0.1)	0	0	0	0	0	0	0	1	0	0	0
Up-regulated (FDR<0.000001)	0	0	0	0	0	0	0	0	0	0	0
Down-regulated (FDR<0.1)	1	0	0	2	0	0	1	4	0	0	0
Down-regulated (FDR<0.000001)	0	0	0	0	0	0	0	0	0	0	0

Fig. S2-44

A

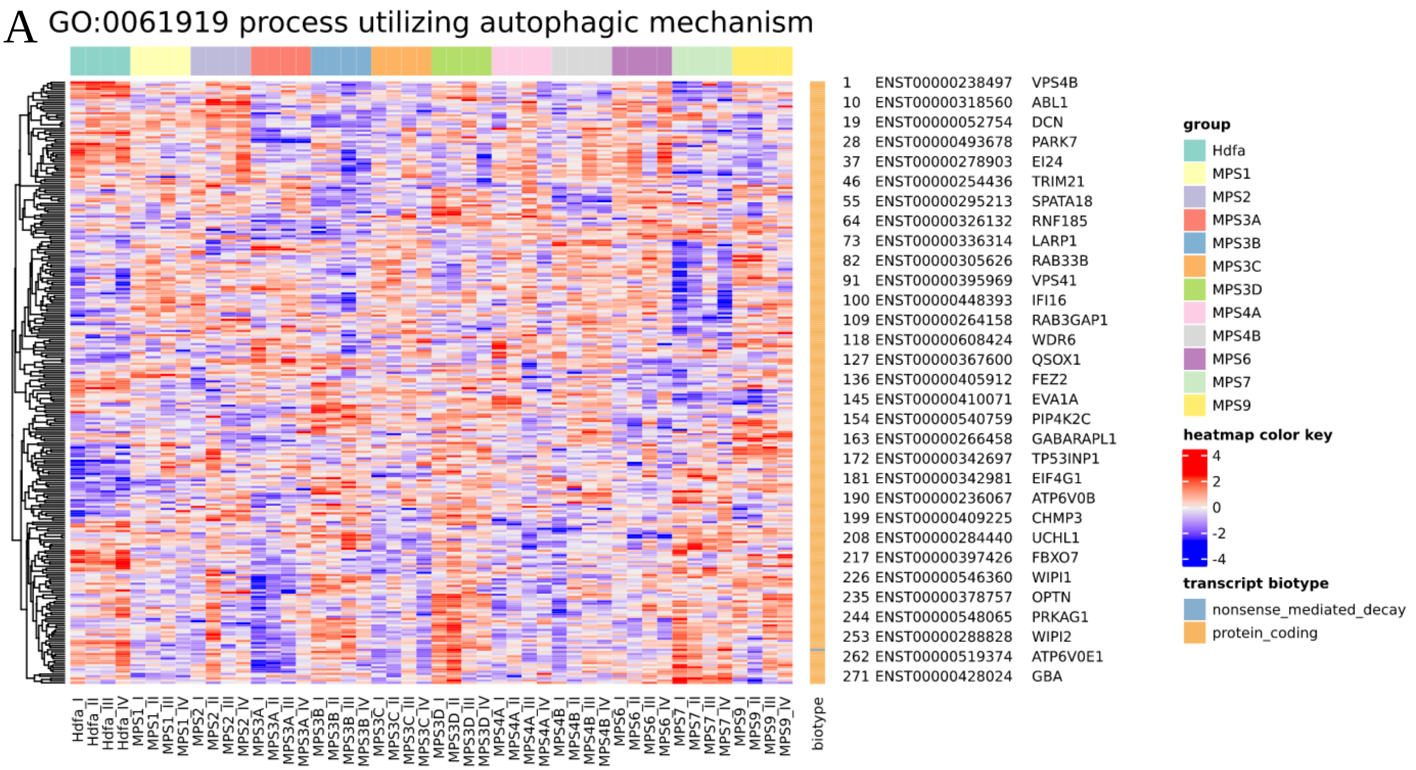

B

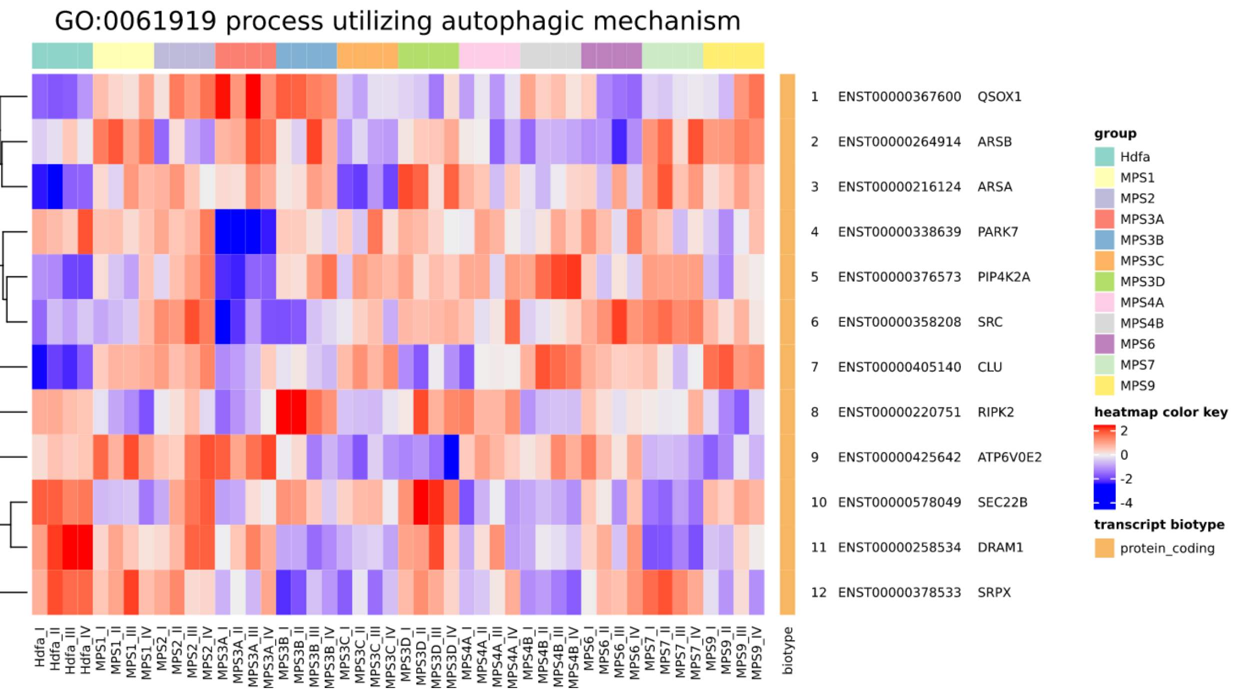

C

Transcripts in **Process utilizing autohagic mechanism**	Significant changes in particular MPS type vs HDFa line										
	I	II	IIIA	IIIB	IIIC	IIID	IVA	IVB	VI	VII	IX
Up-regulated (FDR<0.1)	11	8	5	15	7	11	2	13	6	7	13
Up-regulated (FDR<0.000001)	4	3	2	3	1	2	1	4	2	3	2
Down-regulated (FDR<0.1)	9	3	12	11	9	2	6	10	7	15	8
Down-regulated (FDR<0.000001)	1	0	1	2	1	0	0	3	3	3	1

Fig. S2-45

A

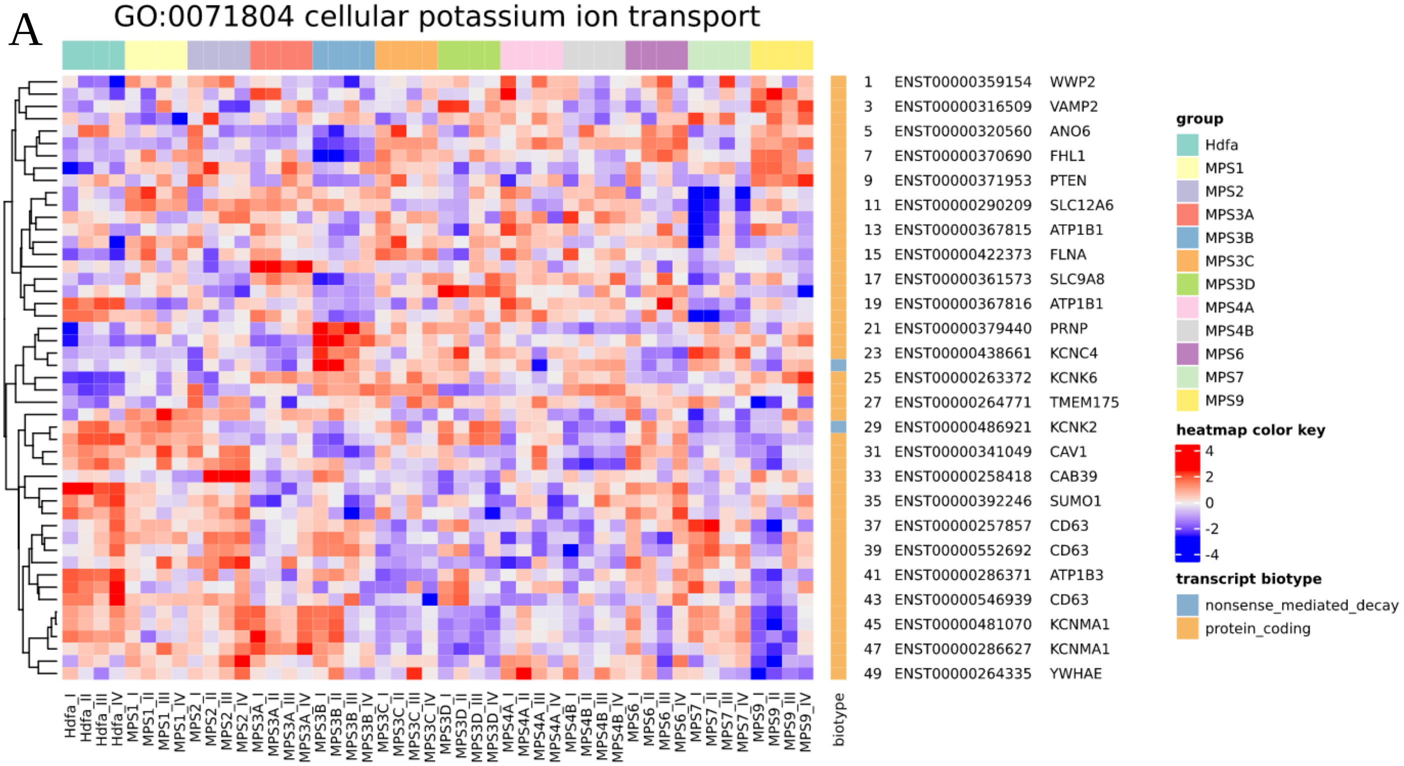

B

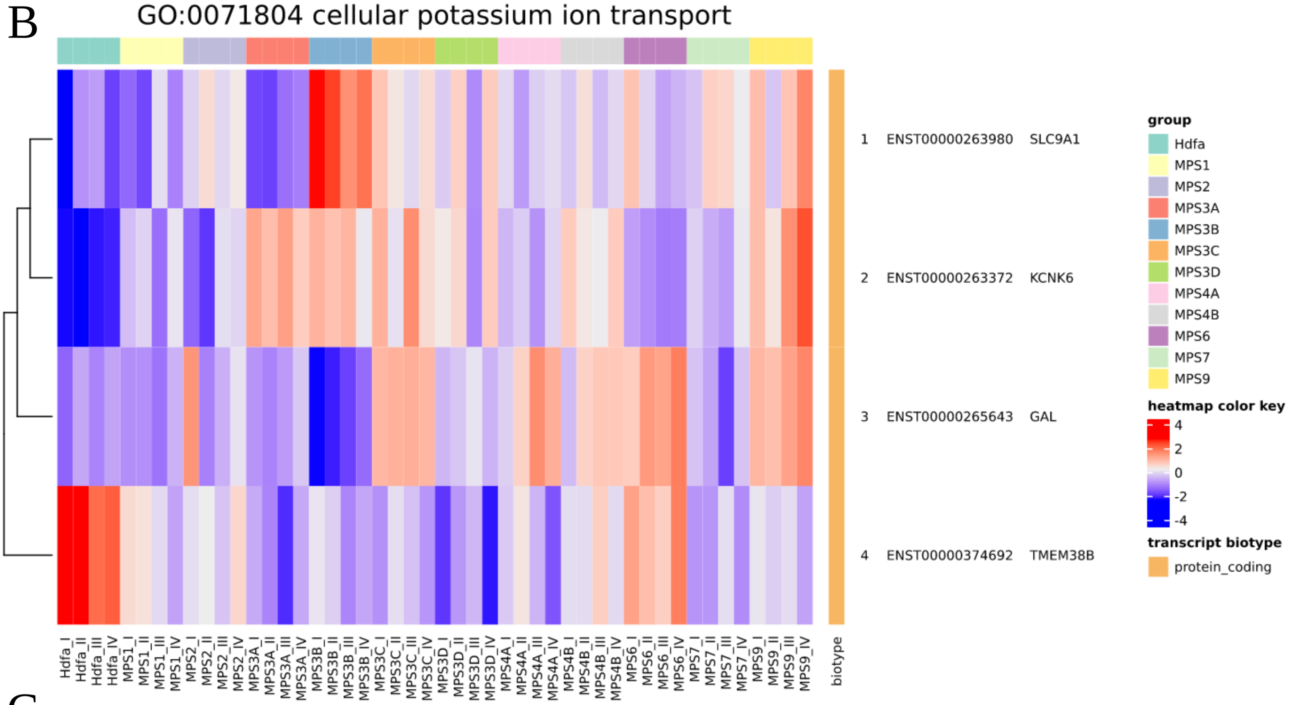

C

Transcripts in **Cellular potassium ion transport** process	Significant changes in particular MPS type vs HDFa line										
	I	II	IIIA	IIIB	IIIC	IIID	IVA	IVB	VI	VII	IX
Up-regulated (FDR<0.1)	0	0	3	3	4	0	1	3	3	1	1
Up-regulated (FDR<0.000001)	0	0	1	2	1	0	1	1	2	1	1
Down-regulated (FDR<0.1)	4	1	3	5	2	5	2	7	0	4	3
Down-regulated (FDR<0.000001)	1	1	1	1	1	1	0	1	4	1	1

Fig. S2-46

A

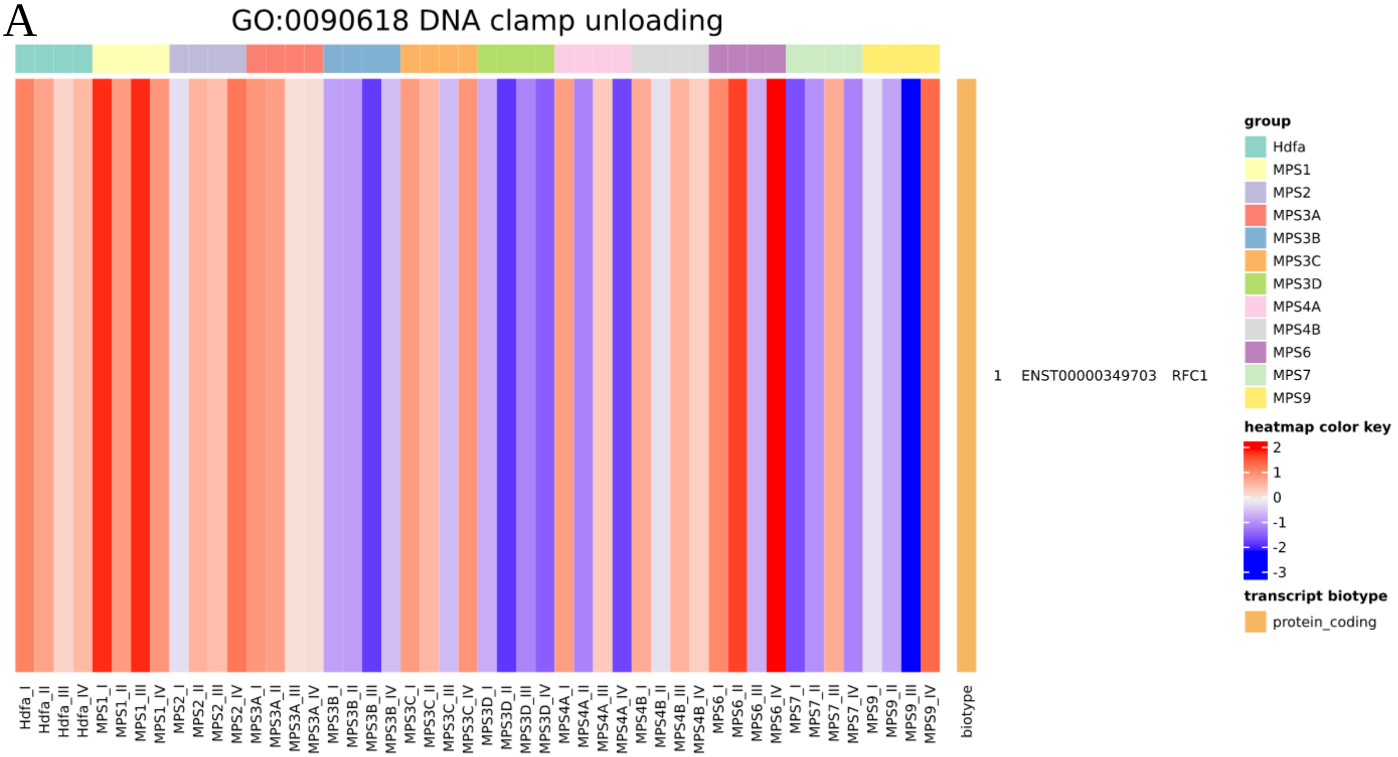

B

Transcripts in **DNA clamp unloading** process	Significant changes in particular MPS type vs HDFa line										
	I	II	IIIA	IIIB	IIIC	IIID	IVA	IVB	VI	VII	IX
Up-regulated (FDR<0.1)	0	0	0	0	0	0	0	0	0	0	0
Up-regulated (FDR<0.000001)	0	0	0	0	0	0	0	0	0	0	0
Down-regulated (FDR<0.1)	0	0	0	0	0	1	0	0	0	0	0
Down-regulated (FDR<0.000001)	0	0	0	0	0	0	0	0	0	0	0

Fig. S2-47

A

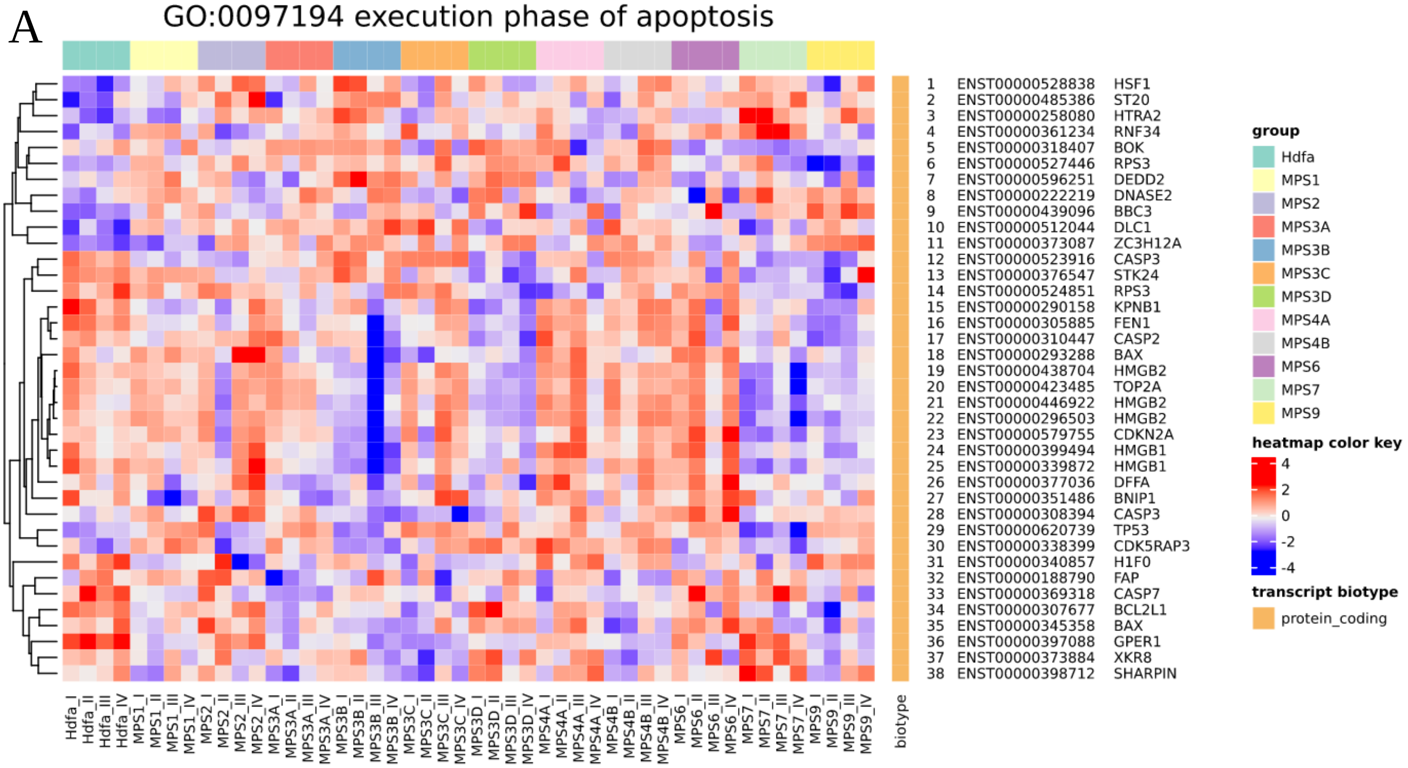

B

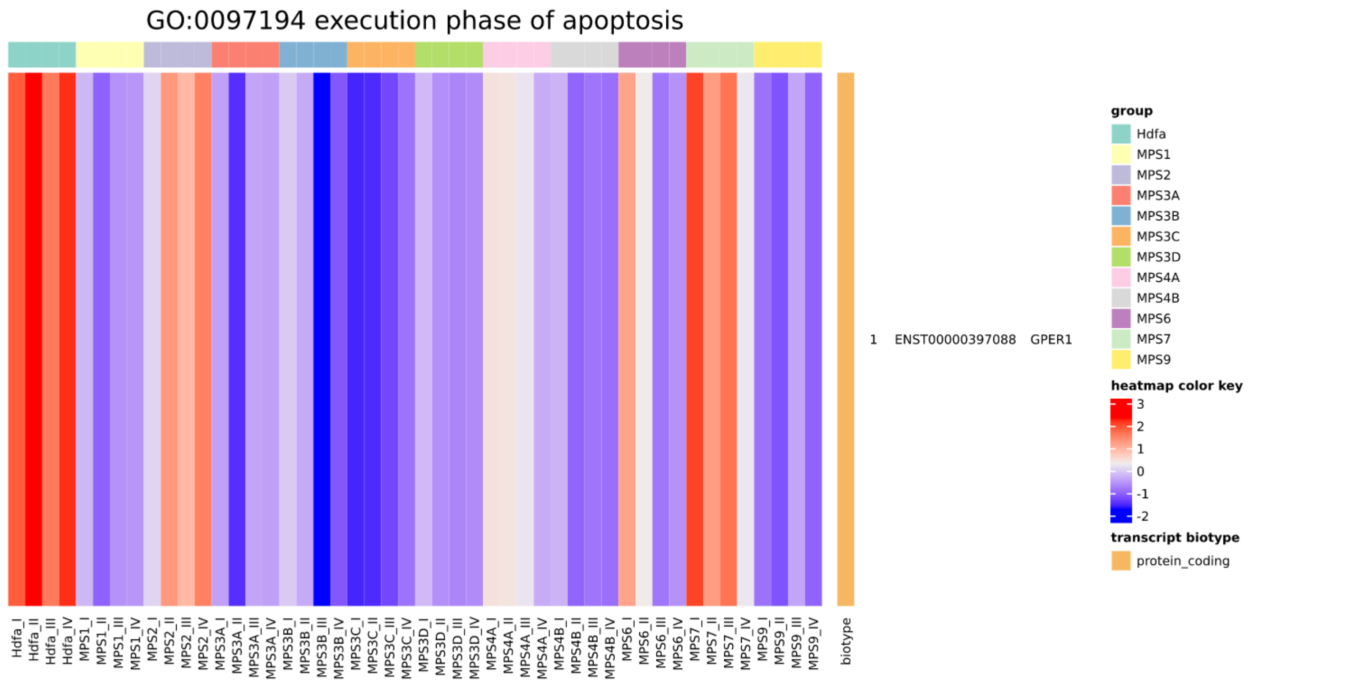

C

Transcripts in **Execution phase of apoptosis** process	Significant changes in particular MPS type vs HDFa line										
	I	II	IIIA	IIIB	IIIC	IIID	IVA	IVB	VI	VII	IX
Up-regulated (FDR<0.1)	0	1	0	2	1	1	0	2	0	1	2
Up-regulated (FDR<0.000001)	0	0	0	0	0	0	0	0	0	0	0
Down-regulated (FDR<0.1)	2	0	1	1	1	2	1	1	0	2	1
Down-regulated (FDR<0.000001)	1	0	1	0	1	1	1	1	5	0	1

Fig. S2-48

A

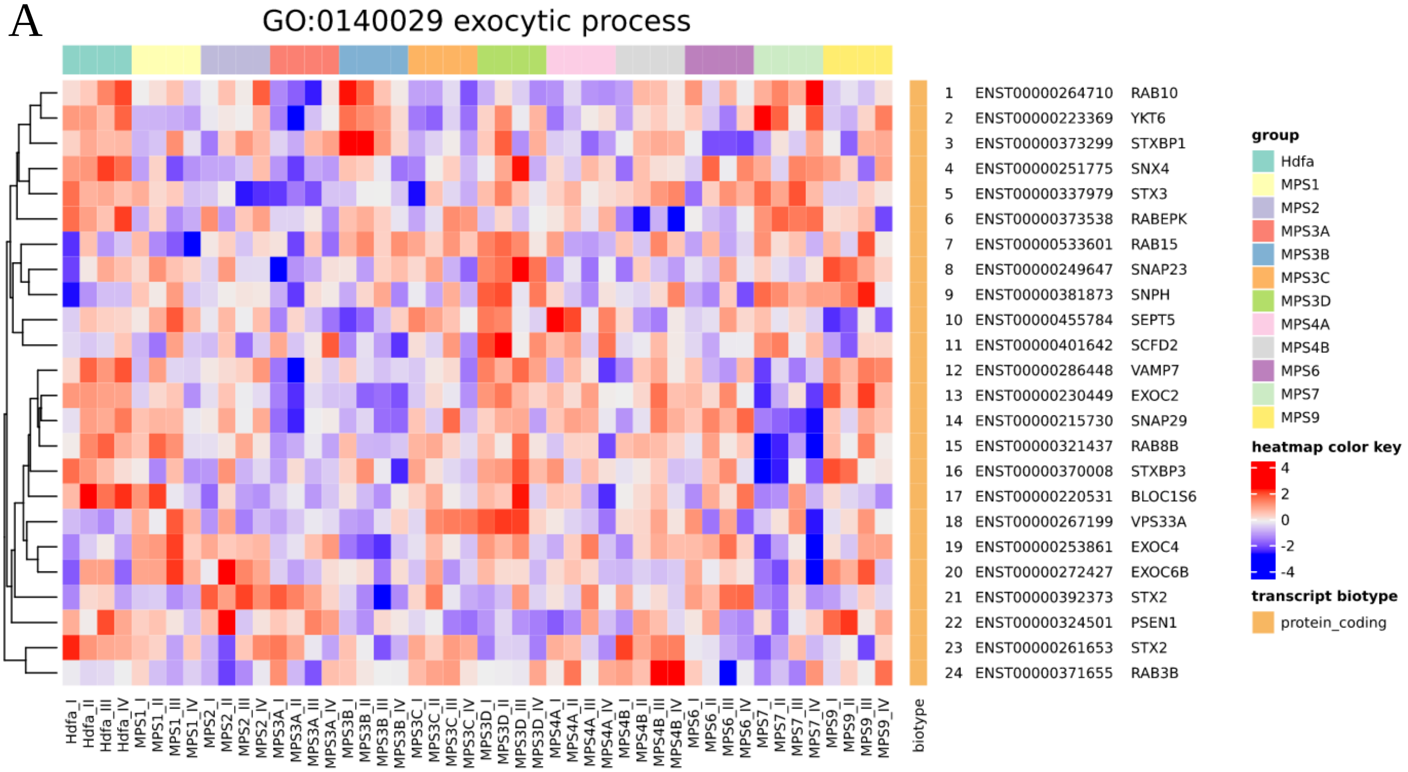

B

Transcripts in **Exocytic process**	Significant changes in particular MPS type vs HDFa line										
	I	II	IIIA	IIIB	IIIC	IIID	IVA	IVB	VI	VII	IX
Up-regulated (FDR<0.1)	0	0	0	0	0	0	0	0	0	0	0
Up-regulated (FDR<0.000001)	0	0	0	0	0	0	0	0	0	0	0
Down-regulated (FDR<0.1)	0	0	1	0	1	0	0	0	0	0	0
Down-regulated (FDR<0.000001)	0	0	0	0	0	0	0	0	0	0	0

Fig. S2-49

A

GO:1990748 cellular detoxification

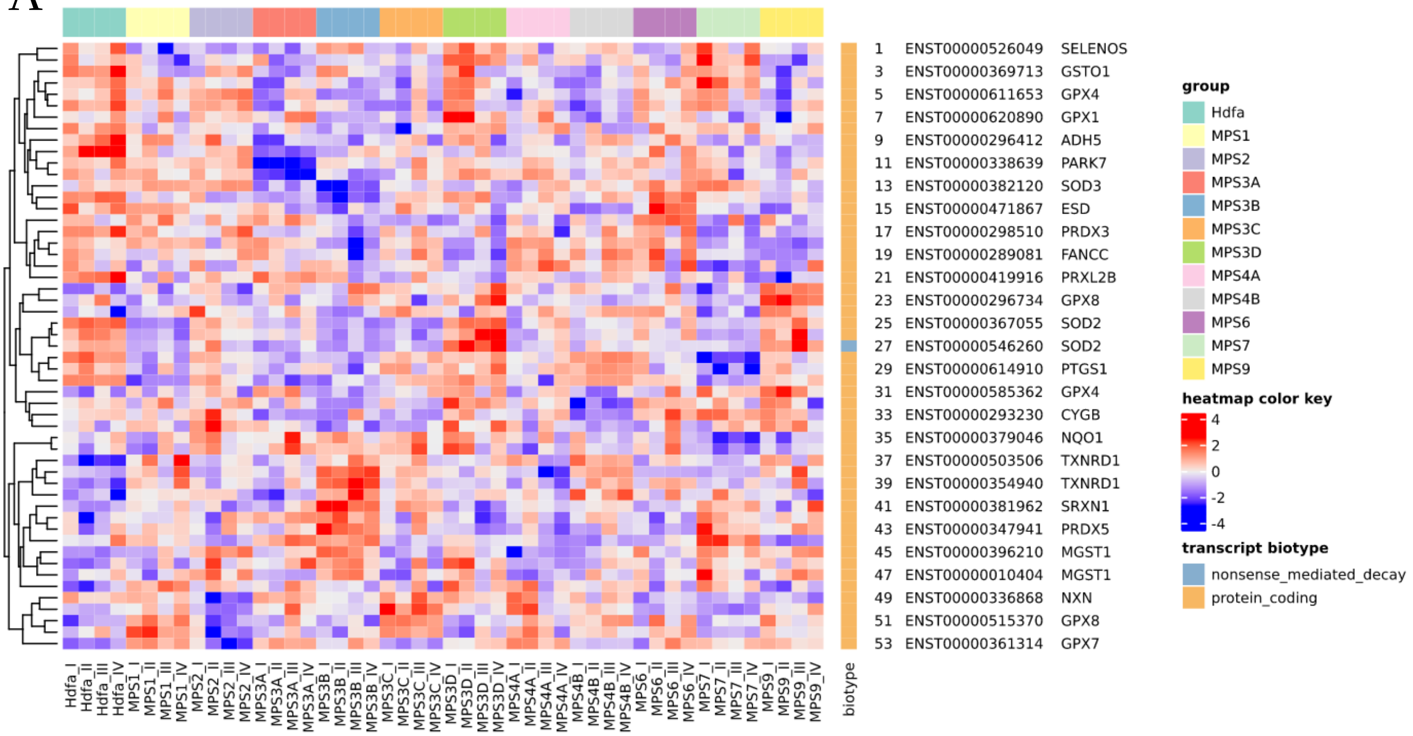

B

GO:1990748 cellular detoxification

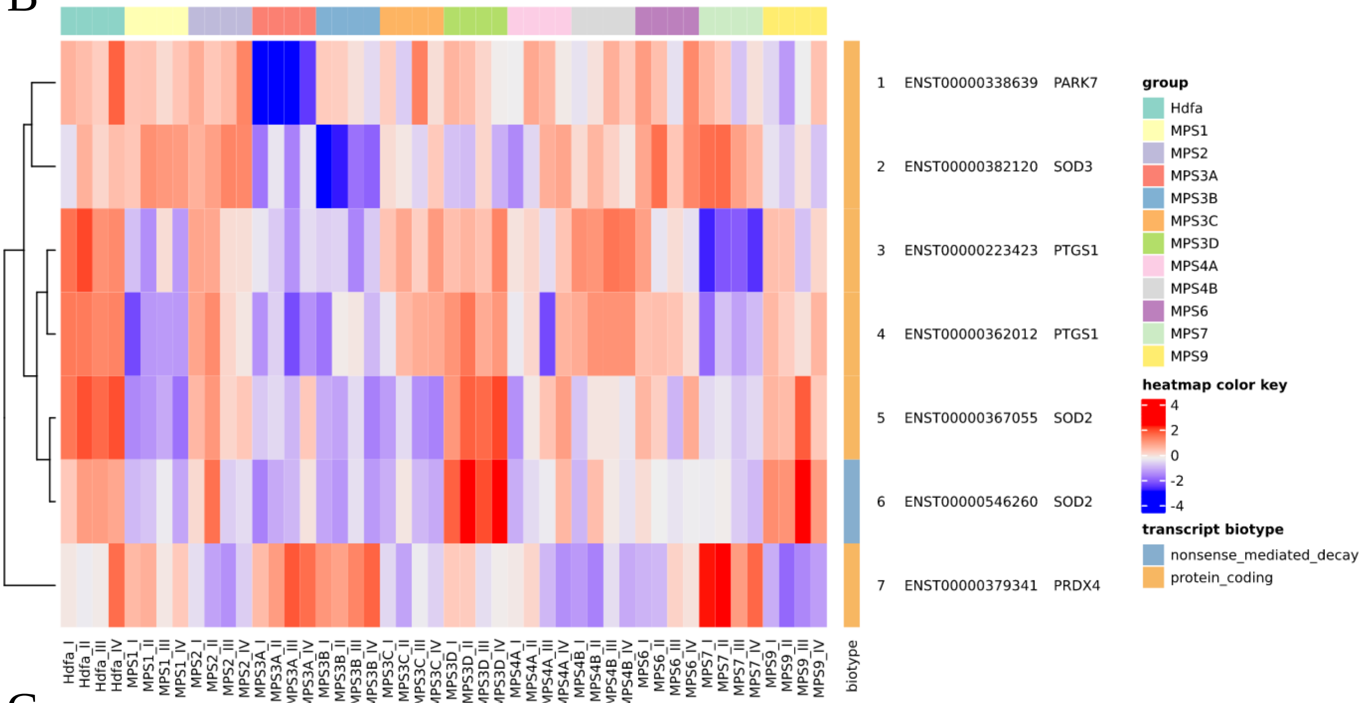

C

Transcripts in **Execution phase of apoptosis** process	Significant changes in particular MPS type vs HDFa line										
	I	II	IIIA	IIIB	IIIC	IIID	IVA	IVB	VI	VII	IX
Up-regulated (FDR<0.1)	1	1	1	4	0	1	1	2	0	2	2
Up-regulated (FDR<0.000001)	0	0	0	0	0	0	0	0	0	0	0
Down-regulated (FDR<0.1)	5	0	6	7	1	1	0	2	1	6	2
Down-regulated (FDR<0.000001)	3	0	2	4	1	0	0	1	6	3	0

Table S2. Statistical analysis of results of gene expression level estimation with log_2 FC>2.5 in MPS I relative to control cells (HDFa).

MPS I		
transcript	log_2 fold change (log_2FC)	p-value
COL8A2	3.19	7.53×10^{-13}
CAPG	2.73	8.05×10^{-15}
CLU	2.82	1.55×10^{-10}
CLU	2.69	4.26×10^{-12}
ADAMTSL1	3.77	4.07×10^{-11}
MFGE8	2.70	4.64×10^{-12}
MFGE8	2.64	4.74×10^{-13}
MN1	2.69	1.29×10^{-11}
LY6K	-3.84	7.67×10^{-15}
LY6K	-3.45	1.56×10^{-13}
SERPINB2	-2.85	6.91×10^{-9}
SNHG5	-2.91	3.08×10^{-13}

Table S3. Statistical analysis of results of gene expression level estimation with log_2 FC>2.5 in MPS II relative to control cells (HDFa).

MPS II		
transcript	log_2 fold change (log_2FC)	p-value
CLU	3.05	1.55×10^{-10}
CLU	2.97	4.26×10^{-12}
MN1	2.78	1.29×10^{-11}
PFN1	3.70	1.04×10^{-10}
LY6K	-2.57	7.67×10^{-15}
SERPINB7	-3.83	1.63×10^{-14}
CLEC2B	-2.86	1.24×10^{-12}

Table S4. Statistical analysis of results of gene expression level estimation with $\log_2$ FC>2.5 in MPS IIIA relative to control cells (HDFa).

MPS IIIA		
transcript	$\log_2$ fold change ($\log_2$FC)	p-value
COL8A2	3.88	7.53×10^{-13}
ADAMTSL1	3.32	4.07×10^{-11}
POSTN	5.27	2.33×10^{-11}
POSTN	5.14	8.84×10^{-12}
MFAP5	6.14	5.18×10^{-17}
MFGE8	3.70	4.64×10^{-12}
MFGE8	3.88	1.26×10^{-8}
MFGE8	3.90	4.74×10^{-13}
FAM167A	2.64	2.75×10^{-12}
PFN1	3.51	1.04×10^{-10}
ENPP2	-2.64	2.40×10^{-13}
CLEC2B	-2.66	1.23×10^{-12}
CTSC	-2.90	7.57×10^{-11}
PTGDS	-6.16	1.29×10^{-8}
PTGS1	-2.57	1.89×10^{-16}
COL18A1	-2.89	2.70×10^{-11}

Table S5. Statistical analysis of results of gene expression level estimation with log_2 FC>2.5 in MPS IIIB relative to control cells (HDFa).

MPS IIIB		
transcript	log_2 fold change (log_2FC)	p-value
ADAMTSL1	3.64	4.08×10^{-11}
POSTN	7.74	8.84×10^{-12}
POSTN	7.25	2.33×10^{-11}
MFAP5	4.99	5.18×10^{-17}
PCOLCE2	3.21	5.71×10^{-11}
MFGE8	3.05	4.75×10^{-13}
MFGE8	2.72	4.65×10^{-12}
FAM167A	3.29	2.75×10^{-12}
NR2F2	3.13	1.92×10^{-15}
NR2F2	2.89	1.10×10^{-14}
CDH2	3.05	1.39×10^{-12}
OXTR	5.20	4.75×10^{-11}
MT1X	3.32	5.24×10^{-10}
AC004556.1	2.96	8.86×10^{-12}
ENPP2	-2.79	2.00×10^{-13}
ENPP2	-3.23	2.41×10^{-13}
SNHG5	-2.68	9.21×10^{-11}
PTGDS	-4.78	1.29×10^{-8}
TRPV2	-3.86	5.78×10^{-14}
KREMEN1	-3.05	2.00×10^{-14}
WISP2	-7.23	7.63×10^{-10}
TNFRSF11B	-2.84	3.10×10^{-16}

Table S6. Statistical analysis of results of gene expression level estimation with $\log_2$ FC>2.5 in MPS IIIC relative to control cells (HDFa).

	MPS IIIC	
transcript	$\log_2$ fold change ($\log_2$FC)	p-value
COL8A2	4.13	7.53×10^{-13}
CAPG	4.27	8.05×10^{-15}
CLU	2.95	4.26×10^{-12}
ADAMTSL1	3.31	4.07×10^{-11}
POSTN	5.41	2.33×10^{-11}
POSTN	5.63	8.84×10^{-12}
MFAP5	4.51	5.18×10^{-17}
FAM167A	2.87	2.75×10^{-12}
NR2F2	2.69	1.10×10^{-14}
CDH2	2.55	1.39×10^{-12}
TENM3	2.55	1.99×10^{-11}
PFN1	3.72	1.04×10^{-10}
OXTR	6.42	4.75×10^{-11}
LY6K	-3.61	7.67×10^{-15}
ENPP2	-2.59	2.40×10^{-13}
EPDR1	-3.40	2.97×10^{-12}
HOXB6	-3.96	1.71×10^{-16}
HOXB5	-2.94	5.76×10^{-15}

Table S7. Statistical analysis of results of gene expression level estimation with log_2 FC>2.5 in MPS IIID relative to control cells (HDFa).

MPS IIID		
transcript	log_2 fold change (log_2FC)	p-value
COL8A2	4.36	7.53×10^{-13}
POSTN	4.97	8.84×10^{-12}
POSTN	5.00	2.33×10^{-11}
PCOLCE2	2.90	5.71×10^{-11}
AC004556.1	3.14	8.86×10^{-12}
LY6K	-4.79	1.56×10^{-13}
LY6K	-5.34	7.67×10^{-15}
SERPINB7	-2.54	1.63×10^{-14}
RPL10	-2.92	9.84×10^{-15}
RPL10	-4.61	9.95×10^{-12}

Table S8. Statistical analysis of results of gene expression level estimation with log_2 FC>2.5 in MPS IVA relative to control cells (HDFa).

MPS IVA		
transcript	log_2 fold change (log_2FC)	p-value
COL8A2	3.26	7.54E-13
CAPG	4.17	8.05E-15

Table S9. Statistical analysis of results of gene expression level estimation with log_2 FC>2.5 in MPS IVB relative to control cells (HDFa).

MPS IVB		
transcript	log_2 fold change (log_2FC)	p-value
CAPG	4.14	8.05×10^{-15}
CLU	3.45	4.27×10^{-12}
CLU	3.42	1.55×10^{-10}
CLU	3.83	2.39×10^{-11}
POSTN	5.83	8.84×10^{-12}
POSTN	5.68	2.33×10^{-11}
MFAP5	4.10	5.18×10^{-17}
PCOLCE2	3.21	5.71×10^{-11}
MFGE8	2.77	4.74×10^{-13}
FAM167A	3.77	2.75×10^{-12}
NR2F2	3.19	1.93×10^{-15}
NR2F2	2.98	1.10×10^{-14}
CDH2	2.94	1.39×10^{-12}
TENM3	3.16	1.99×10^{-11}
OXTR	6.07	4.75×10^{-11}
MT1X	3.49	5.24×10^{-10}
LY6K	-4.28	7.67×10^{-15}
LY6K	-3.94	1.56×10^{-13}
SERPINB7	-2.72	1.63×10^{-14}
ENPP2	-2.54	2.00×10^{-13}
ENPP2	-2.86	2.41×10^{-13}
CLEC2B	-3.08	1.23×10^{-12}
SERPINB2	-3.55	6.91×10^{-9}
KREMEN1	-2.54	2.00×10^{-14}
WISP2	-2.83	7.63×10^{-10}
HOXB6	-3.35	1.71×10^{-16}
HOXB5	-3.21	5.76×10^{-15}

Table S10. Statistical analysis of results of gene expression level estimation with $\log_2$ FC>2.5 in MPS VI relative to control cells (HDFa).

MPS VI		
transcript	$\log_2$ fold change ($\log_2$FC)	p-value
COL8A2	3.60	7.53×10^{-13}
CAPG	3.82	8.05×10^{-15}
CLU	2.58	4.26×10^{-12}
CLU	2.68	1.55×10^{-10}
ADAMTSL1	2.69	4.07×10^{-11}
LY6K	-2.69	7.67×10^{-15}
SERPINB7	-3.09	1.63×10^{-14}

Table S11. Statistical analysis of results of gene expression level estimation with $\log_2$ FC>2.5 in MPS VII relative to control cells (HDFa).

MPS VII		
transcript	$\log_2$ fold change ($\log_2$FC)	p-value
CAPG	4.27	8.05×10^{-15}
MFAP5	4.19	5.18×10^{-17}
PCOLCE2	3.99	5.71×10^{-11}
TENM3	3.72	1.99×10^{-11}
NOTCH3	3.32	7.27×10^{-9}
LY6K	-3.84	7.67×10^{-15}
LY6K	-2.90	1.56×10^{-13}
CLEC2B	-2.83	1.23×10^{-12}
SNHG5	-2.82	3.08×10^{-13}
CTSC	-3.02	7.57×10^{-11}
PTGS1	-5.21	1.89×10^{-16}
PTGS1	-4.31	1.19×10^{-12}
TNFRSF11B	-5.51	3.10×10^{-16}
RPL10	-3.90	9.84×10^{-15}

Table S12. Statistical analysis of results of gene expression level estimation with $\log_2$ FC>2.5 in MPS IX relative to control cells (HDFa).

	MPS IX	
transcript	$\log_2$ fold change ($\log_2$FC)	p-value
COL8A2	3.21	7.53×10^{-13}
CLU	3.46	3.56×10^{-10}
CLU	3.43	4.26×10^{-12}
CLU	3.56	2.39×10^{-11}
ADAMTSL1	2.89	4.07×10^{-11}
POSTN	5.83	2.33×10^{-11}
POSTN	5.70	8.84×10^{-12}
MFAP5	4.40	5.18×10^{-17}
PCOLCE2	3.93	5.71×10^{-11}
NR2F2	2.51	1.92×10^{-15}
CDH2	2.98	1.39×10^{-12}
TENM3	3.69	1.99×10^{-11}
MN1	3.31	1.29×10^{-11}
NOTCH3	2.70	7.27×10^{-9}
LY6K	-3.24	1.56×10^{-13}
LY6K	-4.01	7.67×10^{-15}
SERPINB7	-3.84	1.63×10^{-14}
ENPP2	-3.52	2.00×10^{-13}
ENPP2	-3.70	2.41×10^{-13}
COL18A1	-2.53	2.70×10^{-11}
TRPV2	-3.40	5.78×10^{-14}
EPDR1	-2.77	2.97×10^{-12}
